# A Comprehensive Review of the Structure Elucidation and Biological Activity of Triterpenoids from *Ganoderma* spp.

**DOI:** 10.3390/molecules191117478

**Published:** 2014-10-30

**Authors:** Qing Xia, Huazheng Zhang, Xuefei Sun, Haijuan Zhao, Lingfang Wu, Dan Zhu, Guanghui Yang, Yanyan Shao, Xiaoxue Zhang, Xin Mao, Lanzhen Zhang, Gaimei She

**Affiliations:** 1 School of Chinese Materia Medica, Beijing University of Chinese Medicine, Beijing 100102, China; E-Mails: sdxq1021@163.com (Q.X.); sunxuefei.2008@163.com (X.S.); zhaohaijuan2010@163.com (H.Z.); fanglingwu@163.com (L.W.); hbbdzhudan@163.com (D.Z.); yghui1990@163.com (G.Y.); sunshine4003@126.com (Y.S.); zhangxiaoxue122@163.com (X.Z.); mxmaoxin@126.com (X.M.); 2 School of Basic Medical Sciences, Beijing University of Chinese Medicine, Beijing 100029, China; E-Mail: zhanghuazheng1109@163.com

**Keywords:** *Ganoderma*, triterpenes, chemical structure, ^13^C-NMR data, bioactivity

## Abstract

*Ganoderma* triterpenes (GTs) are the major secondary metabolites of *Ganoderma lucidum*, a traditional Chinese medicine, popularly used for complementary cancer therapy. GTs are lanostane-tetracyclic triterpenes. They have been reported to possess anti-tumor, anti-inflammation, antioxidant, antimicrobial and blood fat reducing effects. To date, 316 GTs have been found and their similar chemical structures have proved difficult to elucidate. This paper compiles 316 naturally occurring triterpenes from *Ganoderma* based on the literature published through January 2013 along with their structures, physiological activities and ^13^C-NMR spectral data.

## 1. Introduction

*Ganoderma lucidum* (Leyss. ex Fr.) Karst, a medicinal fungus called “Lingzhi” in China, is one of the most highly regarded medicinal fungi in the world. It is ranked as rare and precious in the ancient Chinese medical encyclopedias “Shen Nong’s Ben Cao Jing” and “Ben Cao Gang Mu”. The main Lingzhi-producing regions are East China, Southwest China and the provinces of Hebei, and Guangxi. It can be used in the prevention and treatment of various types of disease, such as cancer, hepatopathy, arthritis, hypertension, neurasthenia, debility, *etc.* Its the most attractive characteristics are its immunomodulatory and antitumor activities [1,2,3,4,5,6,7,8]. *Ganoderma* contains many bioactive natural components, including triterpenes (GTs), polysaccharides, proteins, and unsaturated fatty acids. The triterpenes and polysaccharides are deemed to be the primary bioactive compounds of *Ganoderma*.

Kubota isolated ganoderic acid A and ganoderic acid B from *Ganoderma lucidum* (FR.) KARST in 1982 [9]. Since then, more than 316 triterpenes have been isolated from the fruiting bodies, spores, gills, and mycelia of many *Ganoderma* mushrooms. This total was derived from our investigation of the references. As reported, the majority of GTs exhibit a wide range of biological activities, including antitumor, anti-HIV-1, antihypertensive, antiangiogenic, immunomodulatory, antiandrogenic, antihepatitis B, antioxidant, anticomplement, and antimicrobial activities [10,11,12,13]. All GTs are tetracyclic triterpenes. Their chemical structures are more complex than those of other lanostanes, owing to their highly oxidized state. Generally, GTs contain 30 or 27 carbon atoms, and some have 24. The numbers of substituents as well as the positions increase the structural complexity. In this paper, all 316 triterpenes are listed. In accordance with the number of carbon atoms and their molecular features, they can be divided into different structural groups. The ^13^C-NMR data of those triterpenes, elucidation of the compounds’ structures and their bioactivities are discussed. We aim at providing a useful and fast way for identifying GTs. Finally, possible trends and perspectives for future investigation of these mushrooms are also included.

## 2. Ganoderma Triterpenes

**Figure 1:**
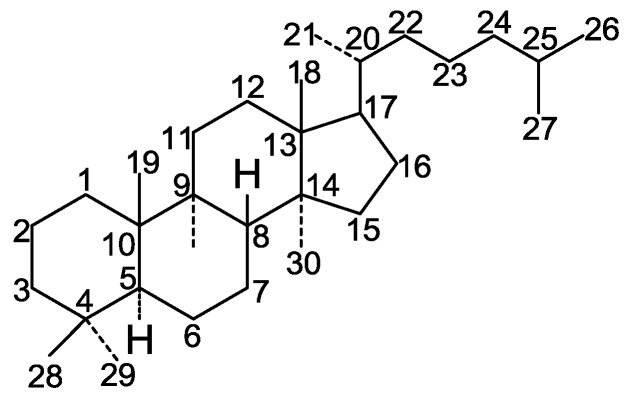
A prototypical lanostane triterpenoid skeleton.

Triterpenes are widely distributed in traditional Chinese medicines. Their structures are considered to be derived from the acyclic precursor squalene. More than 20,000 triterpenes have been isolated and identified from Nature, including squalene, lanostane, dammarane, lupine, oleanane, ursane, and hopane structure types [14,15]. The Ganoderma triterpenes belong to the lanostane triterpenes (Figure 1).

Most of them contain 30 or 27 carbon atoms. A few have 24 carbon atoms. These compounds possesses the same skeleton, namely a *trans* configuration of rings A/B, B/C, C/D and and 10β, 13β, 14α, 17β substituents. Moreover, substituents are always found at the C-3, 7, 11, 12, 15, 22, 23, 24 and 25 positions of the parent nucleus.

**Figure 2:**
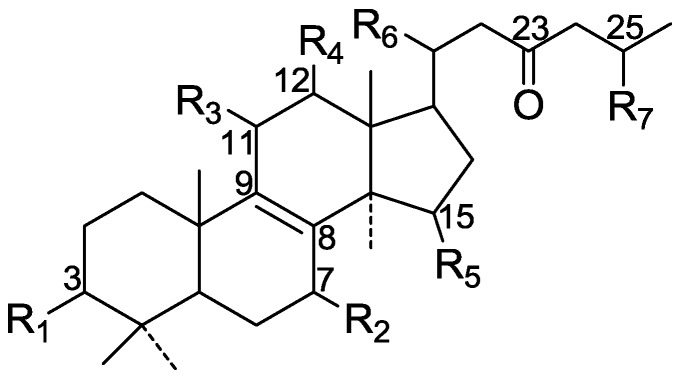
Structures of compounds **1**–**37**.

**Figure 3:**
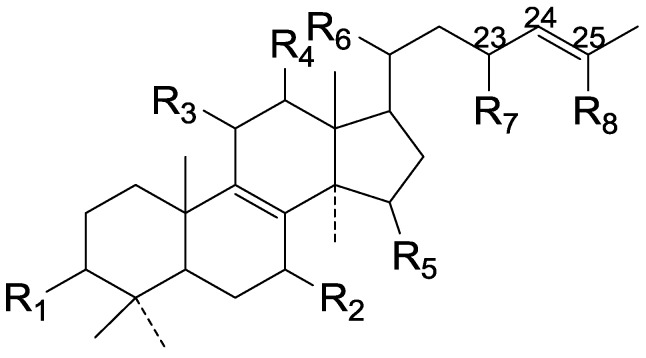
Structures of compounds **38**–**70**.

**Figure 4:**
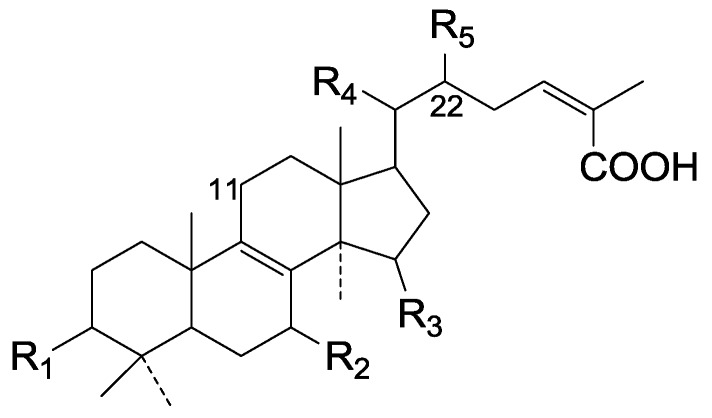
Structures of compounds **71**–**84**.

**Figure 5:**
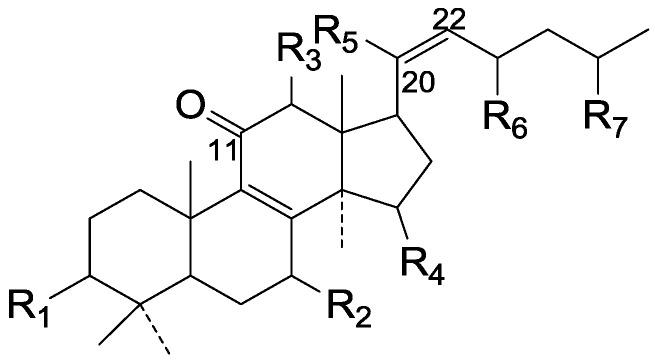
Structures of compounds **85**–**98**.

**Figure 6:**
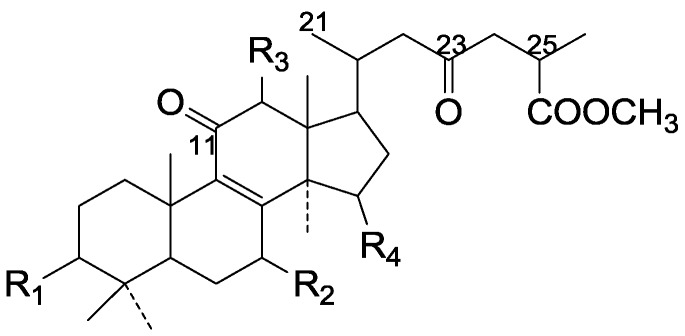
Structures of compounds **99**–**105**.

**Figure 7:**
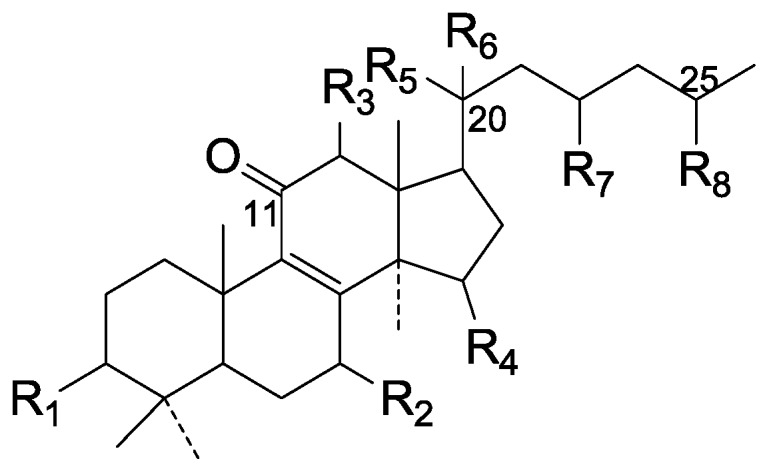
Structures of compounds **106**‒**110**

**Figure 8:**
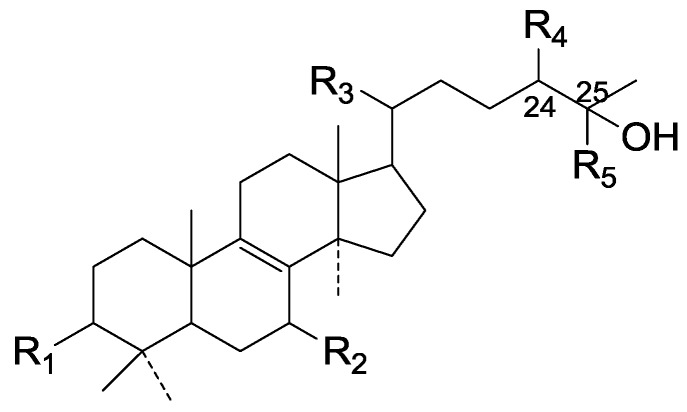
Structures of compounds **111**‒**116**

**Figure 9:**
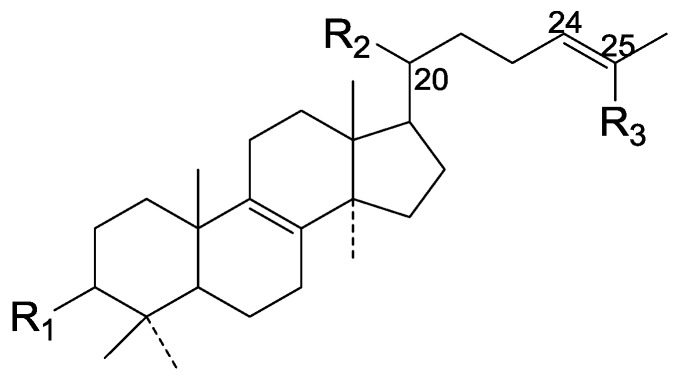
Structures of compounds **117**‒**123**.

**Figure 10:**
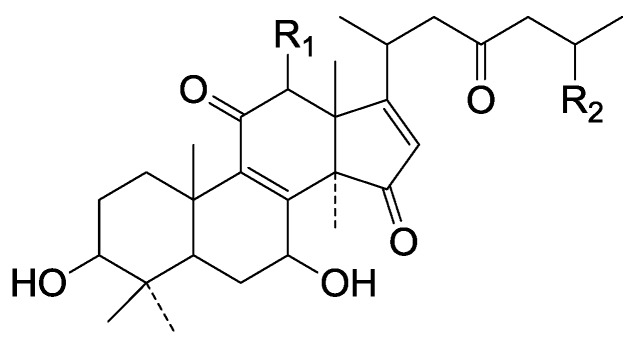
Structures of compounds **124**–**126**.

**Figure 11:**
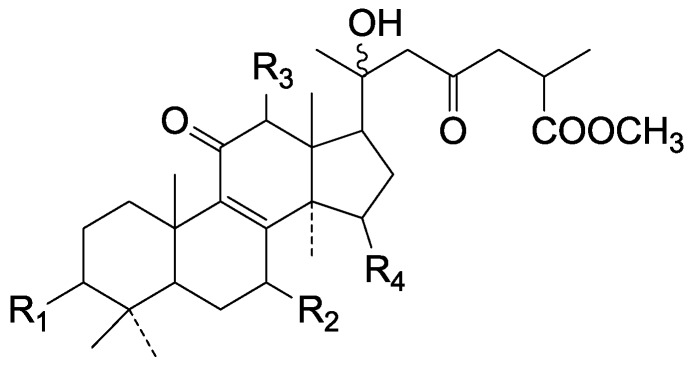
Structures of compounds **127**–**130**.

**Figure 12:**
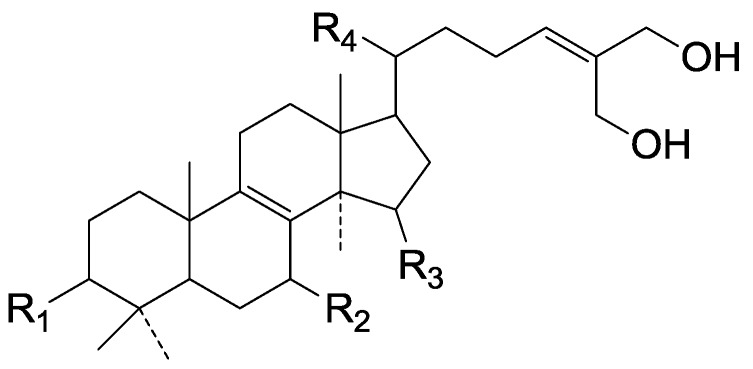
Structures of compounds **131**‒**133**.

**Figure 13:**
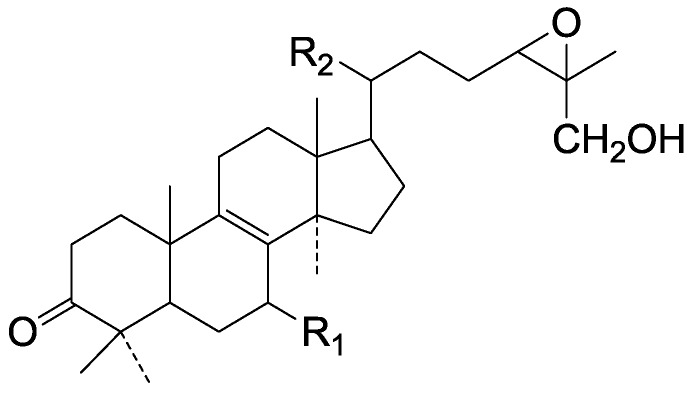
Structures of compounds **134** and **135**.

**Figure 14:**
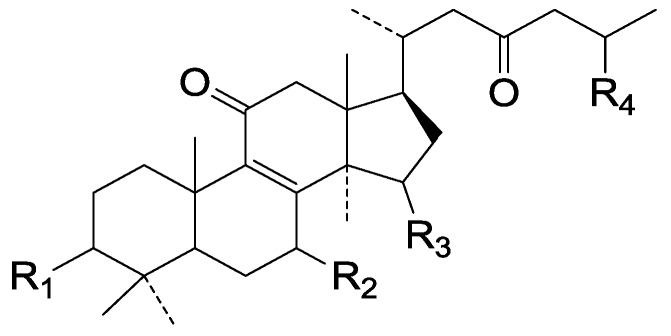
Structures of compounds **136**‒**139**.

**Figure 15:**
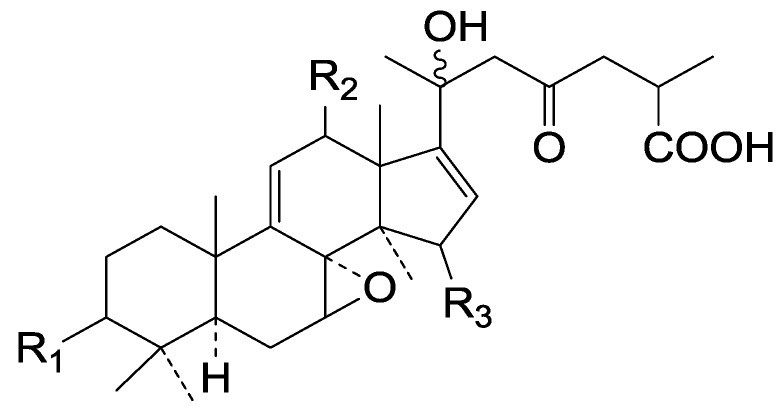
Structures of compounds **140**‒**141**.

**Figure 16:**
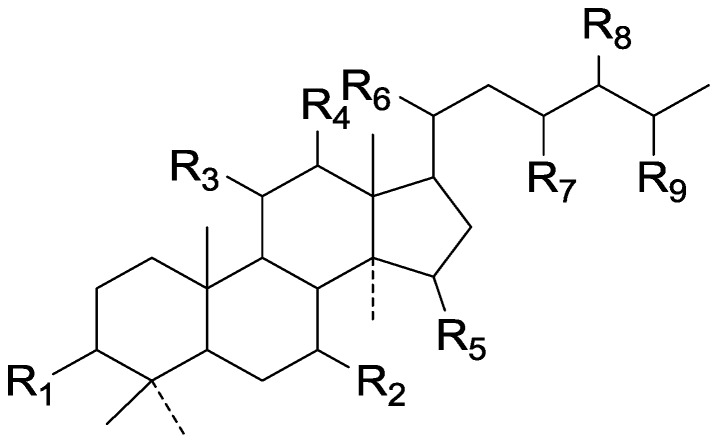
Structures of compounds **142**‒**145**.

**Figure 17:**
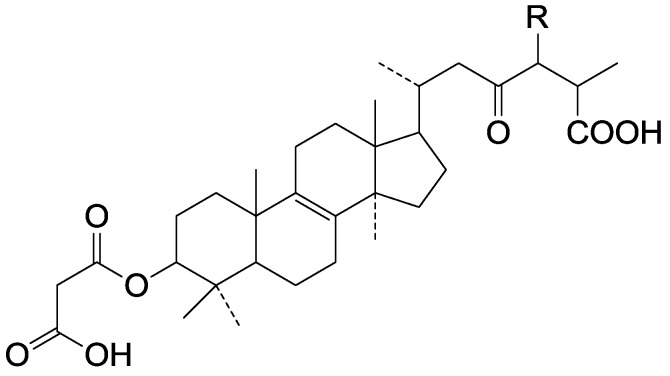
Structures of compounds **146**‒**147**.

**Figure 18:**
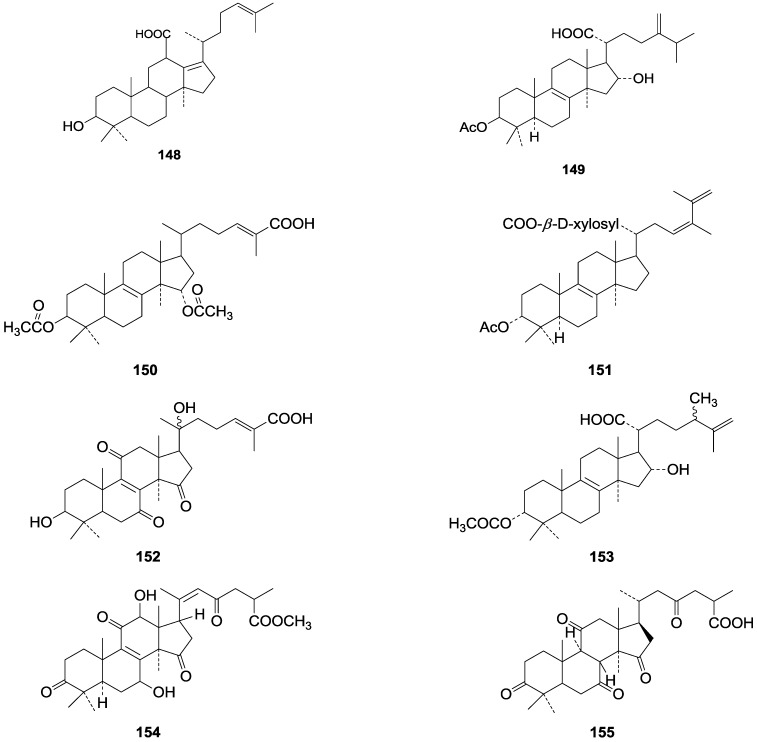
Structures of compounds **148**‒**155**.

**Figure 19:**
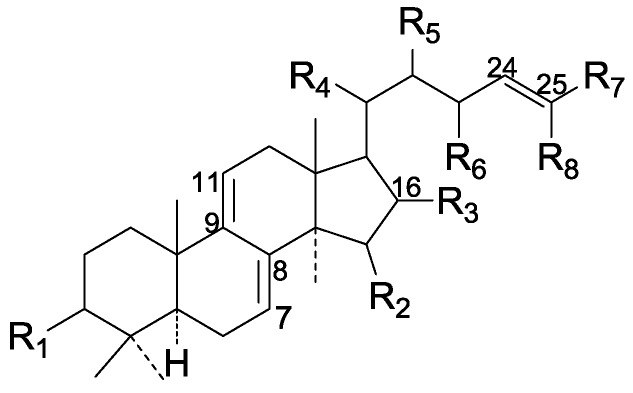
Structures of compounds **156**–**196**.

**Figure 20:**
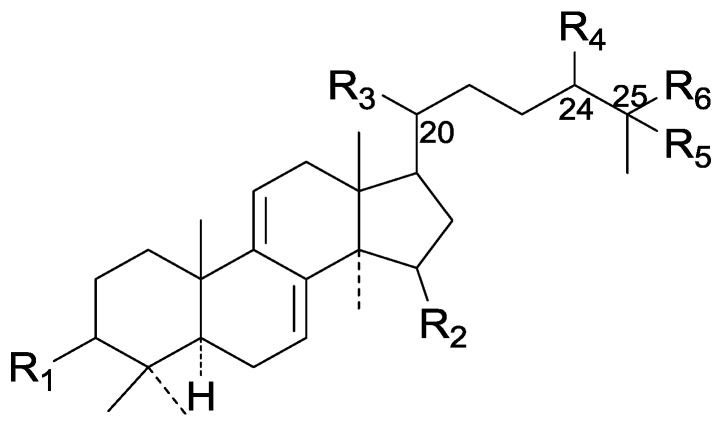
Structures of compounds **197**–**213**.

**Figure 21:**
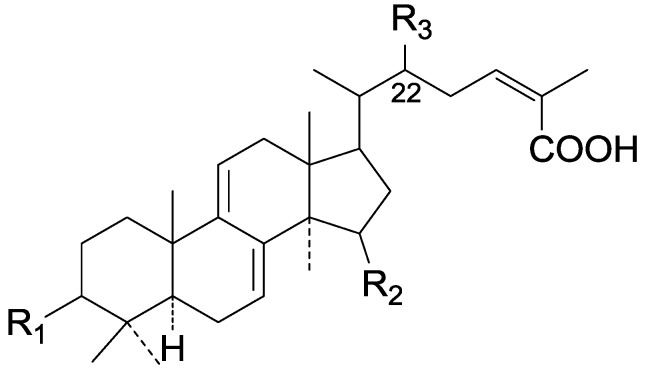
Structures of compounds **214**–**219**.

On the basis of the substituent groups and double bonds in the same position, they are classified into different types. Compounds **1**–**221** (Figure 2, Figure 3, Figure 4, Figure 5, Figure 6, Figure 7, Figure 8, Figure 9, Figure 10, Figure 11, Figure 12, Figure 13, Figure 14, Figure 15, Figure 16, Figure 17, Figure 18, Figure 19, Figure 20 and Figure 21) possess 30 carbon atoms. Among them, **1**–**37** (Figure 2) contain double bonds between C-8 and C-9, a keto group at C-23, and substituent groups at C-3, 7, 11, 12, 15, 25. In this figure, compounds **1**, **3**, **4**, **7**, **8**, **11**–**14**, **17**, **18**, **20**, **25**, **26**, **28**, **31**, **32**, **34**, and **35** possess β-hydroxy groups at C-3, and the others possess a keto group, except 3β-oxo-formyl-7β, 12β-dihydroxy-5α-lanost-11,15,23-trioxo-8-en(*E*)-26-oic acid (**21**) with a formyl located at the C-3 position. Compounds **2**, **3**, **9**–**17**, **19**–**23**, **25**, **27**, **31**, **34**–**36** have hydroxy groups at C-7, and furthermore, **19**, **20**, **22** have α-configurations. What’s more, compounds **1**, **4**, **18**, **24**, **26**, **28**–**30**, **32**, **33**, and **37** have a carbonyl at C-7. In this group, C-11 mainly has a carbonyl substituent except in ganoderic acid Df (**27**) with a β-hydroxyl at this position. The majority of these compounds do not have any substituents at C-12, while compounds **1**, **4**, **24**, **25**, **28**, **29**, **31** possess β-acetyloxy and compounds **21**, **35**–**37** possess β-hydroxyls. All of these compounds display carbonyls or β-hydroxyls at C-15. As to other configurations, both α- and β-C-21, 17β (compounds **5**–**16**, **21**, **28**–**30**, **35**) and 20 α-configurations can be found in this group. Carboxyl, formyl, ethanoyl or butyryl moieties can be found at C-25, most commonly carboxyl. These compounds have extensive biological activities.

**Figure 22:**
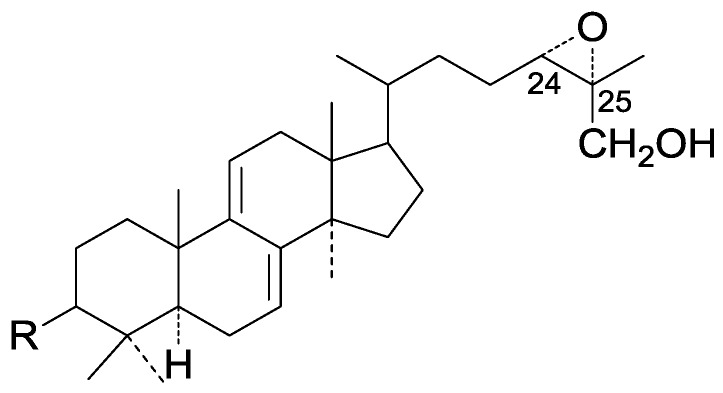
Structure of compounds **220**–**221**.

**Figure 23:**
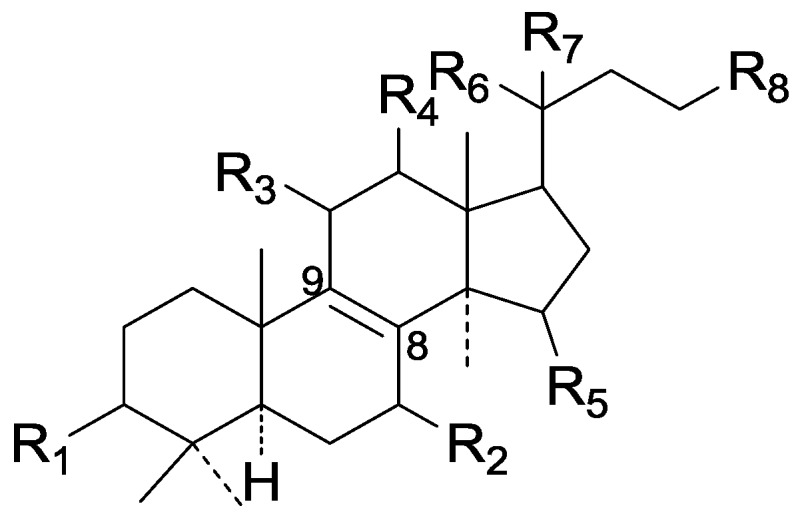
Structures of compounds **222**–**260**.

**Figure 24:**
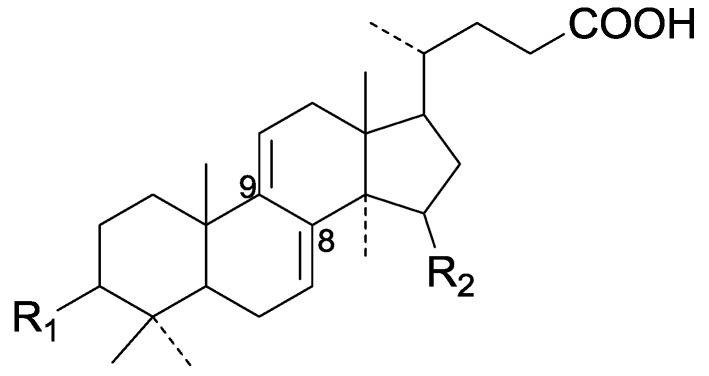
Structures of compounds **261**–**262**.

**Figure 25:**
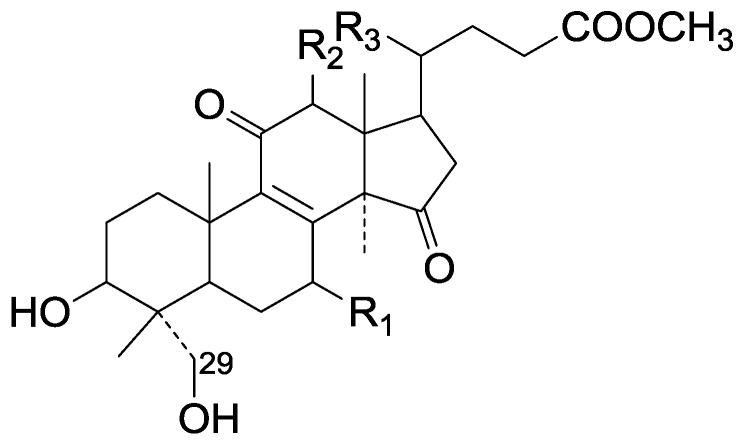
Structures of compounds **263**–**266**.

Compared with the compounds in Figure 2, compounds **38**–**70** in Figure 3 possess double bonds between C-24 and C-25, and have a hydroxy or no substituent at C-23, instead of a carbonyl. Some other substituents are also found at C-3, 7, 11, 12, 15, 23, 25. In this group, lucialdehyde C (**46**) displays strong antitumor activity and ganoderic acid β (**53**) reveals great anti-HIV-1 protease activity. Compounds **71**–**84** (Figure 4) get an acetate substituent at C-22 and no substituent at C-11. Meanwhile, compounds **85**–**98** (Figure 5) have double bonds at C-20(22) and keto groups at C-11. From all the listed structures, we can clearly identify compounds **99**–**105** in Figure 6 by the carboxymethyl substitution at C-25, carbonyl substituent at C-11, a keto group at C-23, and β-configuration of C-21. Compounds **106**–**110** are assigned to the same group owing to the methyl at C-20, carbonyl substituent at C-11, and carboxyl at C-25. Lucidumol A (**111**), ganoderiol C-H (**112**–**115**), and ganoderitriol M (**116**) differ from the others on account of the hydroxy at C-24 and C-25. As is shown in Figure 9, Figure 10, Figure 11, Figure 12, Figure 13, Figure 14, Figure 15, Figure 16 and Figure 17, compounds **117**–**123**, **124**–**126**, **127**–**130**, **131**–**133**, **134**–**135**, **136**–**139**, **140**–**141**, **142**–**145** and **146**–**147** possess extremely similar skeletons. Because of their distinctive skeletons, **148**–**155** are listed independently. There are no double bonds between C-8 and C-9 in compounds **156**–**221**, and two double bonds at C-7(8) and C-9(11), respectively. Among the compounds above, ganoderic acid Jc (**187**), ganoderiol F (**190**), and 15α,26-dihydroxy-5α-lanosta-7,9,24(*E*)-trien-3-one (**212**) showed remarkable antitumor activity. Significant anti-HIV-1 protease activity has been expressed in ganoderic acid S1 (**159**) and ganodermic acid T-Q (**183**). Compounds **156**–**196** (Figure 19) have the same skeleton with substituents at C-3, 15, 16, 20, 22, 23, 25 and double bonds at C-24(25). In this group, 3α, 16α-dihydroxylanosta-7,9(11),24-trien-21-oic acid (**157**), 3α, 16α, 26-trihydroxylanosta-7,9(11), 24-trien-21-oic acid (**158**) and 16α-hydroxy-3-oxolanosta-7,9(11),24-trien-21-oic acid (**196**) possess a hydroxyl at C-16 and carboxyl at C-20. Compounds **197**–**213** (Figure 20) have the same position of substituents. They possess an α- or β-configuration at C-21. The majority have double bonds between C-24 and C-25, except some with hydroxyl, acetoxyl or no substituents at C-24 and C-25. Compounds **214**–**219** (Figure 21) have a hydroxy or acetoxyl at C-22, while epoxyganoderiol B (**220**) and C (**221**) (Figure 22) possess an epoxy at C-24(25). Compounds **222**–**266** have the basic skeleton of 27 carbon atoms. Furthermore, they are also subdivided into different groups due to the difference of substituents and position of double bonds. The C-8(9) double bonds are the same in compounds **222**–**260** (Figure 23). 4,4,14α-Trimethyl-5α-chol-7,9(11)-dien-3-oxo-24-oic acid (**261**) and ganoderic acid Jd (**262**) (Figure 24) get two double bonds at C-7(8) and C-9(11), respectively. Compared with the compounds in Figure 22, compounds **263**–**266** in Figure 25 have hydroxy substituents at C-29. Compounds **267**–**287** are divided into different groups on account of their characteristic skeletons. We list the structures of compounds **288**–**307** successively, in consideration of the number of substituents and the substituents’ complicated positions. Fornicatin B(**308**), G(**309**), A(**310**), H(**311**) and australic acid (**312**) are 3,4-*seco*-trinorlanostane triterpenoids. In addition, compounds **313**–**316** only have 24 carbon atoms. The names, corresponding plant resources and references of the compounds are compiled in Table 1, Table 2, Table 3, Table 4, Table 5, Table 6, Table 7, Table 8, Table 9, Table 10 and Table 11.

**Table 1:** *Ganoderma* triterpenes **1**–**37** in Figure 2.

No.	Compound Name	Source	Ref.
**1**	*n*-Butyl ganoderate H (*n*-butyl 12β-acetoxy-3β-hydroxy-7,11,15,23-tetraoxo-5α-lanost-8-en-26-oate)	*G. lucidum* (fruit bodies)	[16]
**2**	Butyl ganoderate A	*G. lucidum* (fruit bodies)	[17]
**3**	Butyl ganoderate B	*G. lucidum* (fruit bodies)	[17]
**4**	Ganoderic acid α (12β-acetoxy-3β, 15β-dihydroxy-7,11,23-trioxo-5α-lanosta-8-en-26-oic acid)	*G. lucidum* (fruit bodies)	[18]
**5**	Ganolucidic acid A	*G. lucidum* (gill surface)	[19]
**6**	Methyl ganolucidate A (methyl 15α-hydroxy-3,11,23-trioxo-5α-lanost-8-en-26-oate)	*G. lucidum* (gill surface)	[19,20]
**7**	Ganolucidic acid B	*G. lucidum* (gill surface)	[19]
**8**	Methyl ganolucidate B	*G. lucidum* (gill surface)	[19,20]
**9**	Ganoderic acid A (7β, 15α*-*dihydroxy-3,11,23-trioxo-5α-lanost-8-en-26-oic acid)	*G. lucidum*	[9,21]
**10**	Methyl ganoderate A (methyl 7β, 15α-dihydroxy-3,11,23-trioxo-5α-lanost-8-en-26-oate)	*G. lucidum*	[21]
**11**	Ganoderic acid B (3β, 7β-dihydroxy-11,15,23-trioxo-5α-lanost-8-en-26-oic acid)	*G. lucidum*	[21]
**12**	Methyl ganoderate B (methyl 3β, 7β-dihydroxy-11,15,23-trioxo-5α-lanost-8-en-26-oate)	*G. lucidum*	[9,21]
**13**	Ganoderic acid C (3β, 7β, 15α-trihydroxy-11,23-dioxo-5α-lanost-8-en-26-oic acid)	*G. lucidum*	[21]
**14**	Methyl ganoderate C	*G. lucidum*	[21]
**15**	Ganoderic acid D (7β-hydroxy-3,11,15,23-tetraoxo-5α-lanost-8-en-26-oic acid)	*G. lucidum*	[21]
**16**	Methyl ganoderate D	*G. lucidum*	[21]
**17**	Methyl ganoderate C_2_ (methyl 3β, 7β, 15α-trihydroxy-11,23-dioxo-5α-lanost-8-en-26-oate)	*G. lucidum* (gills)	[22]
**18**	Methyl ganoderate K	*G. lucidum* (gills)	[22,23]
**19**	Compound B_8_	*G. lucidum* (gills)	[22]
**20**	Compound B_9_	*G. lucidum* (gills)	[22]
**21**	3β-Oxo-formyl-7β, 12β-dihydroxy-5α-lanost-11,15,23-trioxo-8-en(*E*)-26-oic acid	*G. lucidum* (fruit bodies)	[24]
**22**	Ganoderic acid B_8_	*G. lucidum* (fruit bodies)	[25]
**23**	Ganoderic acid C_1_	*G. lucidum* (fruit bodies)	[25]
**24**	12β-Acetoxy-3,7,11,15,23-pentaoxo-5α-lanosta-8-en-26-oic acid ethyl ester	*G. lucidum*	[26]
**25**	3β, 7β-Dihydroxy-12β-acetoxy-11,15,23-trioxo-5α-lanosta-8-en-26-oic acid methyl ester	*G. lucidum*	[27]
**26**	3β-Hydroxy-7,11,12,15,23-pentaoxolanost-8-en-26-oic acid	*G. lucidum* (fruit bodies)	[28]
**27**	Ganoderic acid Df (7β, 11β-dihydroxy-3,15,23-trioxo-5α*-*lanosta-8-en-26-oic acid)	*G. lucidum*	[29]
**28**	Ganoderic acid H	*G. lucidum* (gill surface)	[30]
**29**	Ganoderic acid F (12β-acetoxy-3,7,11,15-pentaoxo-5α-lanost-8-en-26-oic acid)	*G. lucidum* (dried fruit bodies)	[31]
**30**	Ganoderic acid E (3,7,11,15,23-pentaoxo-5α*-*lanost-8-en-26-oic acid)	*G. lucidum* (dried fruit bodies)	[31]
**31**	Ganoderic acid K	*G. lucidum* (fruit bodies)	[32]
**32**	Ganoderic acid AM_1_	*G. lucidum* (fruit bodies)	[32]
**33**	Ganoderic acid J	*G. lucidum* (fruit bodies)	[32]
**34**	Ganoderic acid C_2_ (3β, 7β, 15α-trihydroxy-11,23-dioxo-5α-lanosta-8-en-26-oic acid)	*G. lucidum* (gills)	[22]
**35**	Ganoderic acid G (3β, 7β, 15β-trihydroxy-11,15,23-trioxo-5α-lanosta-8-en-26-oic acid)	*G. lucidum* (dried fruit bodies)	[31]
**36**	7β, 12β-Dihydroxy-3,11,15,23-tetraoxo-5α-lanosta-8-en-26-oic acid	*G. lucidum*	[26]
**37**	12β-Hydroxy-3,7,11,15,23-pentaoxo-5α-lanosta-8-en-26-oic acid	*G. lucidum*	[26]

**Table 2:** *Ganoderma* triterpenes (**38**–**70**) in Figure 3.

No.	Compound Name	Source	Ref.
**38**	Ganoderic acid GS-1 (7β-hydroxy-3,11,15-trioxolanosta-8,24(*E*)-dien-26-oic acid)	*G. sinense* (fruit bodies)	[33]
**39**	Ganoderic acid GS-2 (7β, 15α-dihydroxy-3,11-dioxolanosta-8,24(*E*)-dien-26-oic acid)	*G. sinense* (fruit bodies)	[33]
**40**	Ganoderic acid GS-3 (12β-acetoxy-3β, 7β-dihydroxy-11,15-dioxo-lanosta-8,24(*E*)-dien-26-oic acid)	*G. sinense* (fruit bodies)	[33]
**41**	Ganoderic acid AP_2_ (12β, 15α*-*diacetoxy-3β-hydroxy-11-oxolanost-8,24(*E*)-dien-26-oic acid)	*G.* *applanatum* (fruit bodies)	[34]
**42**	23*S*-Hydroxy-3,7,11,15-tetraoxolanost-8,24*E*-diene-26-oic acid	*G. lucidum* (fruit bodies)	[32]
**43**	7-Oxoganoderic acid Z (3β*-*hydroxy-7-oxo-5α-lanosta-8,24(*E*)-dien-26-oic acid)	*G. lucidum* (fruit bodies)	[35]
**44**	Ganoderic acid LM_2_ ((23*S*) 7β,-dihydroxy-3,11,15-trioxo-5α-lanosta-8,24-dien-26-oic acid)	*G. lucidum* (fruit bodies)	[36]
**45**	Lucialdehyde B ((24*E*)-3,7-dioxo-5α-lanosta-8,24-dien-26-al)	*G. lucidum* (fruit bodies)	[25]
**46**	Lucialdehyde C ((24*E*)-3β-hydroxy-7-oxo-5α-lanosta-8,24-dien-26-al)	*G. lucidum* (fruit bodies)	[25]
**47**	Ganoderic acid γ ((23*S*)-7β, 15α, 23-trihydroxy-3,11-dioxolanosta-8,24(*E*)-diene-26-oic acid)	*G. lucidum* (spores)	[37]
**48**	Ganoderic acid *δ* ((23*S*)-7α, 15α, 23-trihydroxy-3,11-dioxolanosta-8,24(*E*)-diene-26-oic acid)	*G. lucidum* (spores)	[37]
**49**	Ganoderic acid *ε* ((23*S*)-3β, 7β, 23-trihydroxy-11,15-dioxolanosta-8,24(*E*)-diene-26-oic acid)	*G. lucidum* (spores)	[37]
**50**	Ganoderic acid *ζ* ((23*S*)-3β, 23-dihydroxy-7,11,15-trioxolanosta-8,24(*E*)-diene-26-oic acid)	*G. lucidum* (spores)	[37]
**51**	Ganoderic acid *η* ((23*S*)-3β, 7β, 12β, 23-tetrahydroxy-11,15-dioxolanosta-8,24(*E*)-diene-26-oic acid)	*G. lucidum* (spores)	[37]
**52**	Ganoderic acid *θ* ((23*S*)-3β, 12β, 23-trihydroxy-7,11,15-trioxolanosta-8,24(*E*)-diene-26-oic acid)	*G. lucidum* (spores)	[37]
**53**	Ganoderic acid β (3β, 7β-dihydroxy-11,15-dioxolanosta-8,24(*E*)-dien-26-oic acid)	*G. lucidum* (spores)	[38]
**54**	Ganolucidic acid E (15α-hydroxy-3,11-dioxo-5α-lanosta-8,24*E*-dien-26-oic acid)	*G. lucidum* (fruit bodies)	[39]
**55**	Ganoderal B (7α-hydroxy-3-oxo-5α-lanosta-8,24*E*-dien-26-al)	*G. lucidum*	[40]
**56**	Ganoderic acid Ma (3α, 7α-diacetoxy-15α-hydroxy-5α-lanost-8,24*E*-dien-26-oic acid)	*G. lucidum* (fruit bodies)	[41]
**57**	Lucialdehyde D (3,7,11-trioxo-5α-lanosta-8,24-diene-26-al)	*G.* *pfeifferi* (fruit bodies)	[42]
**58**	Ganoderone A (5α-lanosta-8,24-diene-26-hydroxy-3,7-dione)	*G.* *pfeifferi* (fruit bodies)	[42]
**59**	ganoderic acid Mi (3α-acetoxy-15α-hydroxy-7α-methoxy-5α-lanost-8,24*E*-dien-26-oic acid)	*G. lucidum* (mycelial mat)	[43]
**60**	11α-Hydroxy-3,7-dioxo-5α-lanosta-8,24(*E*)-dien-26-oic acid	*G. lucidum*	[26]
**61**	11β-Hydroxy-3,7-dioxo-5α-lanosta-8,24(*E*)-dien-26-oic acid	*G. lucidum*	[26]
**62**	Lucidadiol (5α-lanosta-8,24-dien-3β, 26-dihydroxy-7-one)	*G. lucidum*	[44]
**63**	Lucidal (5α-lanosta-8,24*E*-dien-3β-hydroxy-7-on-26-al)	*G. lucidum*	[44]
**64**	Ganoderic acid DM (3,7-dioxo-8,24(*E*)-dien-lanosta-26-oic acid)	*G. lucidum* (cultured fruit bodies)	[45]
**65**	Ganoderic acid V	*G.* *orbiforme*	[46]
**66**	Ganolucidic acid γa (3β, 7β, 15α, 23-tetrahydroxy-11-oxo-5α-lanosta-8,24-dien-26-oic acid)	*G. sinense* (fruit bodies)	[47]
**67**	Ganolucidate F (3β, 15α, 23-trihydroxy-11-oxo-5α-lanosta-8,24-dien-26-oic acid)	*G. sinense* (fruit bodies)	[47]
**68**	Lucialdehyde E (7β, 15α-dihydroxy-3,11-dioxo-5α-lanosta-8,24-dien-26-al)	*G.* *lucidum* (spores)	[48]
**69**	Ganolucidic acid D	*G.* *lucidum* (spores)	[37]
**70**	Ganoderic acid W	*G.* *lucidum* (fruit bodies)	[41]

**Table:** 

Cpd	R1	R2	R3	R4	R5	R6	R7
**1**	β-OH	=O	=O	β-*O*-Ac	=O	α-CH_3_	COOBu
**2**	=O	β-OH	=O	H	α-OH	α-CH_3_	COOBu
**3**	β-OH	β-OH	=O	H	=O	α-CH_3_	COOBu
**4**	β-OH	=O	=O	β-*O*-Ac	α-OH	α-CH_3_	COOH
**5**	=O	H	=O	H	α-OH	β-CH_3_	COOH
**6**	=O	H	=O	H	α-OH	β-CH_3_	COOCH_3_
**7**	β-OH	H	=O	H	α-OH	β-CH_3_	COOH
**8**	β-OH	H	=O	H	α-OH	β-CH_3_	COOCH_3_
**9**	=O	β-OH	=O	H	α-OH	β-CH_3_	COOH
**10**	=O	β-OH	=O	H	α-OH	β-CH_3_	COOCH_3_
**11**	β-OH	β-OH	=O	H	=O	β-CH_3_	COOH
**12**	β-OH	β-OH	=O	H	=O	β-CH_3_	COOCH_3_
**13**	β-OH	β-OH	=O	H	α-OH	β-CH_3_	COOH
**14**	β-OH	β-OH	=O	H	α-OH	β-CH_3_	COOCH_3_
**15**	=O	β-OH	=O	H	=O	β-CH_3_	COOH
**16**	=O	β-OH	=O	H	=O	β-CH_3_	COOCH_3_
**17**	β-OH	β-OH	=O	H	α-OH	α-CH_3_	COOCH_3_
**18**	β-OH	=O	=O	H	α-OH	α-CH_3_	COOCH_3_
**19**	=O	α-OH	=O	H	α-OH	α-CH_3_	COOCH_3_
**20**	β-OH	α-OH	=O	H	α-OH	α-CH_3_	COOCH_3_
**21**	*O*-CHO	β-OH	=O	β-OH	=O	β-CH_3_	COOH
**22**	=O	α-OH	=O	H	α-OH	α-CH_3_	COOH
**23**	=O	β-OH	=O	H	=O	α-CH_3_	COOH
**24**	=O	=O	=O	β-*O*-COCH_3_	=O	α-CH_3_	COOEt
**25**	β-OH	β-OH	=O	β-*O*-COCH_3_	=O	α-CH_3_	COOCH_3_
**26**	β-OH	=O	=O	=O	=O	α-CH_3_	COOH
**27**	=O	β-OH	β-OH	H	=O	α-CH_3_	COOH
**28**	β-OH	=O	=O	β-*O*-Ac	=O	β-CH_3_	COOH
**29**	=O	=O	=O	β-*O*-Ac	=O	β-CH_3_	COOH
**30**	=O	=O	=O	H	=O	β-CH_3_	COOH
**31**	β-OH	β-OH	=O	β-*O*-Ac	=O	α-CH_3_	COOH
**32**	β-OH	=O	=O	H	=O	α-CH_3_	COOH
**33**	=O	=O	=O	H	α-OH	α-CH_3_	COOH
**34**	β-OH	β-OH	=O	H	α-OH	α-CH_3_	COOH
**35**	β-OH	β-OH	=O	β-OH	=O	β-CH_3_	COOH
**36**	=O	β-OH	=O	β-OH	=O	α-CH_3_	COOH
**37**	=O	=O	=O	β-OH	=O	α-CH_3_	COOH

**Table:** 

Cpd	R1	R2	R3	R4	R5	R6	R7	R8
**38**	=O	β-OH	=O	H	=O	α-CH_3_	H	COOH
**39**	=O	β-OH	=O	H	α-OH	α-CH_3_	H	COOH
**40**	β-OH	β-OH	=O	β-*O*-Ac	=O	α-CH_3_	H	COOH
**41**	β-OH	H	=O	β-*O*-Ac	α-*O*-Ac	α-CH_3_	H	COOH
**42**	=O	=O	=O	H	=O	α-CH_3_	β-OH	COOH
**43**	β-OH	=O	H	H	H	β-CH_3_	H	COOH
**44**	=O	OH	=O	H	=O	α-CH_3_	OH	COOH
**45**	=O	=O	H	H	H	α-CH_3_	H	CHO
**46**	β-OH	=O	H	H	H	α-CH_3_	H	CHO
**47**	=O	β-OH	=O	H	α-OH	α-CH_3_	β-OH	COOH
**48**	=O	α-OH	=O	H	α-OH	α-CH_3_	β-OH	COOH
**49**	β-OH	β-OH	=O	H	=O	α-CH_3_	β-OH	COOH
**50**	β-OH	=O	=O	H	=O	α-CH_3_	β-OH	COOH
**51**	β-OH	β-OH	=O	β-OH	=O	α-CH_3_	β-OH	COOH
**52**	β-OH	=O	=O	β-OH	=O	α-CH_3_	β-OH	COOH
**53**	β-OH	β-OH	=O	H	=O	α-CH_3_	H	COOH
**54**	=O	H	=O	H	α-OH	β-CH_3_	H	COOH
**55**	=O	α-OH	H	H	H	β-CH_3_	H	CHO
**56**	α-*O*-Ac	α-*O*-Ac	H	H	α-OH	β-CH_3_	H	COOH
**57**	=O	=O	=O	H	H	α-CH_3_	H	CHO
**58**	=O	=O	H	H	H	α-CH_3_	H	CH_2_OH
**59**	α-*O*-Ac	α-*O*-CH_3_	H	H	α-OH	β-CH_3_	H	COOH
**60**	=O	=O	α-OH	H	H	α-CH_3_	H	COOH
**61**	=O	=O	β-OH	H	H	α-CH_3_	H	COOH
**62**	β-OH	=O	H	H	H	α-CH_3_	H	CH_2_OH
**63**	β-OH	=O	H	H	H	α-CH_3_	H	CHO
**64**	=O	=O	H	H	H	α-CH_3_	H	COOH
**65**	=O	α-OH	H	H	α-*O*-Ac	α-CH_3_	H	COOH
**66**	β-OH	β-OH	=O	H	α-OH	α-CH_3_	β-OH	COOH
**67**	β-OH	H	=O	H	α-OH	α-CH_3_	β-OH	COOH
**68**	=O	β-OH	=O	H	α-OH	α-CH_3_	H	CHO
**69**	=O	H	=O	H	α-OH	α-CH_3_	β-OH	COOH
**70**	α-*O*-Ac	α-OH	H	H	α-*O*-Ac	β-CH_3_	H	COOH

**Table 3:** *Ganoderma* triterpenes **71**–**98** in Figure 4 and Figure 5.

No.	Compound Name	Source	Ref.
**71**	Ganoderic acid Mb (3α, 15α, 22-triacetoxy-7α-hydroxy-5α-lanost-8,24*E*-dien-26-oic acid)	*G.* *lucidum* (fruit bodies)	[41]
**72**	Ganoderic acid Mc (3α, 7α, 22-triacetoxy-15α-hydroxy-5α-lanost-8,24*E*-dien-26-oic acid)	*G.* *lucidum* (fruit bodies)	[41]
**73**	Ganoderic acid Md (3α, 22-diacetoxy-7α-methoxy-5α-lanost-8,24*E*-dien-26-oic acid)	*G.* *lucidum* (fruit bodies)	[41]
**74**	Ganoderic acid Mg (3α, 22-diacetoxy-15α-hydroxy-7α-methoxy-5α-lanost-8,24*E*-dien-26-oic acid)	*G.* *lucidum* (mycelial mat)	[43]
**75**	Ganoderic acid Mh (3α, 22-diacetoxy-7α, 15α-dihydroxy-5α-lanost-8,24*E*-dien-26-oic acid)	*G.* *lucidum* (mycelial mat)	[43]
**76**	Ganoderic acid Mj (22-acetoxy-3α-hydroxy-7α-methoxy-5α-lanost-8,24*E*-dien-26-oic acid)	*G.* *lucidum* (mycelial mat)	[43]
**77**	3α, 22β-Diacetoxy-7α-hydroxyl-5α-lanost-8,24*E*-dien-26-oic acid	*G.* *lucidum* (mycelial mat)	[49]
**78**	Ganorbiformin B	*G. orbiforme*	[46]
**79**	Ganorbiformin C	*G. orbiforme*	[46]
**80**	Ganorbiformin D	*G. orbiforme*	[46]
**81**	Ganorbiformin E	*G. orbiforme*	[46]
**82**	Ganorbiformin F	*G. orbiforme*	[46]
**83**	Ganoderic acid O ((22*S*, 24*E*)-3α, l5α, 22-triacetoxy-7α-hydroxy-5α-lanosta-7,24-dien-26-oic acid)	*G.* *lucidum* (cultured mycelium)	[50]
**84**	7-*O*-Methylganoderic acid O ((22*S*, 24*E*)-3α, l5α, 22-triacetoxy-7α-methoxy-5α-lanosta-8,24-dien-26-oic acid)	*G.* *Lucidum* (cultured mycelium)	[50]
**85**	12β-Acetoxy-3β-hydroxy-7,11,15,23-tetraoxo-lanost-8,20*E*-diene-26-oic acid	*G.* *lucidum* (fruit bodies)	[32]
**86**	23-Dihydroganoderenic acid D (7β, 23ξ-dihydroxy-3,11,15-trioxolanosta-8,20*E*(22)-dien-26-oic acid)	*G.* *applanatum* (fruit bodies)	[51]
**87**	Methyl ganoderenate D (7β-hydroxy-3,11,15,23-tetraoxolanosta-8,20*E*(22)-dien-26-oic acid methyl ester)	*G.* *applanatum* (fruit bodies)	[51]
**88**	Ganoderenic acid A ((20*E*)-7β, 15α-dihydroxy-3,11,23-trioxo-5α-lanost-8,20-dien-26-oic acid)	*G.* *lucidum* (dried fruit bodies)	[31]
**89**	Ganoderenic acid B ((20*E*)-3β, 7β-dihydroxy-11,15,23-trioxo-5α-lanost-8,20-dien-26-oic acid)	*G.* *lucidum* (dried fruit bodies)	[31]
**90**	Ganoderenic acid C ((20*E*)-3β, 7β, 15α-trihydroxy-11,23-dioxo-5α-lanost-8,20-dien-26-oic acid)	*G.* *lucidum* (dried fruit bodies)	[31]
**91**	Ganoderenic acid D ((20*E*)-7β-hydroxy-3,11,15,23-tetraoxo-5α-lanost-8,20-dien-26-oic acid)	*G.* *lucidum* (dried fruit bodies)	[31]
**92**	12β-Acetoxy-7β-hydroxy-3,11,15,23-tetraoxo-5α-lanosta-8,20-dien-26-oic acid	*G.* *lucidum*	[26]
**93**	Ganoderenic acid F (3,7,11,15,23-pentaoxo-5α-lanosta-8,20*E*-dien-26-oic acid)	*G. applanatum* (fruit bodies)	[52]
**94**	Ganoderenic acid G (15α-hydroxy-3,7,11,23-tetraoxo-5α-lanosta-8,20*E*-dien-26-oic acid)	*G.* *applanatum* (fruit bodies)	[52]
**95**	Methy ganoderenate H (methyl 3β-hydroxy-7,11,15,23-tetraoxo-5α-lanosta-8,20*E*-dien-26-oate)	*G.* *applanatum* (fruit bodies)	[52]
**96**	Methyl ganoderenate I (3β, 15α-dihydroxy-7,11,23-trioxo-5α-lanosta-8,20*E*-dien-26-oate)	*G.* *applanatum* (fruit bodies)	[52]
**97**	Ganoderenic acid H	*G.* *lucidum* (fruit bodies)	[32]
**98**	12β-Acetoxy-3β, 7β-dihydroxy-11,15,23-trioxo-5α-lanosta-8,20-dien-26-oic acid	*G.* *lucidum*	[26]

**Table:** 

Cpd	R1	R2	R3	R4	R5
**71**	α-*O*-Ac	α-OH	α-*O*-Ac	β-CH_3_	ξ-*O*-Ac
**72**	α-*O*-Ac	α-*O*-Ac	α-OH	β-CH_3_	ξ-*O*-Ac
**73**	α-*O*-Ac	α-*O*-CH_3_	H	β-CH_3_	ξ-*O*-Ac
**74**	α-*O*-Ac	α-*O*-CH_3_	α-OH	β-CH_3_	ξ-*O*-Ac
**75**	α-*O*-Ac	α-OH	α-OH	β-CH_3_	ξ-*O*-Ac
**76**	α-OH	α-*O*-CH_3_	H	β-CH_3_	ξ-*O*-Ac
**77**	α-*O*-Ac	α-OH	H	α-CH_3_	β-*O*-Ac
**78**	β-*O*-Ac	=O	H	α-CH_3_	β-*O*-Ac
**79**	β-OH	=O	H	α-CH_3_	β-*O*-Ac
**80**	=O	α-OH	α-*O*-Ac	α-CH_3_	β-*O*-Ac
**81**	=O	α-OH	H	α-CH_3_	β-*O*-Ac
**82**	=O	α-*O*-CH_3_	H	α-CH_3_	β-*O*-Ac
**83**	α-*O*-Ac	α-OH	α-*O*-Ac	α-CH_3_	β-*O*-Ac
**84**	α-*O*-Ac	α-*O*-CH_3_	α-*O*-Ac	α-CH_3_	β-*O*-Ac

**Table:** 

Cpd	R1	R2	R3	R4	R5	R6	R7
**85**	β-OH	=O	β-*O*-Ac	=O	β-CH_3_	=O	COOH
**86**	=O	β-OH	H	=O	β-CH_3_	ξ-OH	ξ-COOH
**87**	=O	β-OH	H	=O	β-CH_3_	=O	ξ-COOCH_3_
**88**	=O	β-OH	H	α-OH	β-CH_3_	=O	COOH
**89**	β-OH	β-OH	H	=O	β-CH_3_	=O	COOH
**90**	β-OH	β-OH	H	α-OH	β-CH_3_	=O	COOH
**91**	=O	β-OH	H	=O	β-CH_3_	=O	COOH
**92**	=O	β-OH	β-*O*-Ac	=O	β-CH_3_	=O	COOH
**93**	=O	=O	H	=O	β-CH_3_	=O	COOH
**94**	=O	=O	H	α-OH	β-CH_3_	=O	COOH
**95**	β-OH	=O	H	=O	β-CH_3_	=O	COCH_3_
**96**	β-OH	=O	H	α-OH	β-CH_3_	=O	COCH_3_
**97**	β-OH	=O	H	=O	β-CH_3_	=O	COOH
**98**	β-OH	β-OH	β-*O*-Ac	=O	α-CH_3_	=O	COOH

**Table 4:** *Ganoderma* triterpenes **99**–**123** in Figure 6, Figure 7, Figure 8 and Figure 9.

No.	Compound Name	Source	Ref.
**99**	Methyl ganoderate D (methyl 3β, 7β, 15α-trihydroxy-11,23-dioxo-5α-lanost-8-en-26-oate)	*G.* *lucidum* (fruit bodies)	[53,54]
**100**	Methyl ganoderate E (methyl 3β, 7β, 15α-trihydroxy-11,23-dioxo-5α-lanost-8-en-26-oate)	*G.* *lucidum* (gills)	[54,55]
**101**	Methyl ganoderate F (methyl 12β-acetoxy-3,7,11,15,23-pentaoxo-5α-lanost-8-en-26-oate)	*G.* *lucidum* (gills)	[56]
**102**	Methyl ganoderate H (methyl 3β-hydroxy-12β-acetoxy-7,11,15,23-tetraoxo-5α-lanost-8-en-26-oate)	*G.* *lucidum* (gills)	[30,56]
**103**	Methyl ganoderate G (methyl 3β, 7β, 12β-trihydroxy-11,15,23-trioxo-5α-lanost-8-en-26-oate)	*G.* *lucidum*	[20]
**104**	Compound C_5_	*G.* *lucidum* (gill surface)	[30]
**105**	Compound C_6_	*G.* *lucidum* (gill surface)	[30]
**106**	Ganoderic acid AP_3_ (15α, 20ξ-dihydroxy-3,7,11,23-tetraoxolanost-8-en-26-oic acid)	*G.* *applanatum* (fruit bodies)	[34]
**107**	23-Dihydroganoderic acid I (3β, 7β, 20,23ξ-tetrahydroxy-11,15-dioxolanosta-8-en-26-oic acid)	*G. applanatum* (fruit bodies)	[51]
**108**	23-Dihydroganoderic acid N (7β, 20,23ξ-trihydroxy-3,11,15-trioxolanosta-8-en-26-oic acid)	*G.* *applanatum* (fruit bodies)	[51]
**109**	20-Hydroxylganoderic acid G	*G.* *lucidum* (fruit bodies)	[57]
**110**	Ganoderic acid I	*G.* *lucidum* (gills)	[22]
**111**	Lucidumol A ((24*S*)-24,25-dihydroxylanost-8-ene-3,7-dione)	*G.* *lucidum* (spores)	[38]
**112**	Ganoderiol C (7α-ethoxy-24,25,26-trihydroxy-5α-lanost-8-en-3-one)	*G.* *lucidum* (fruit bodies)	[39]
**113**	Ganoderiol D (24,25,26-trihydroxy-5α-lanost-8-en-3,7-dione)	*G.* *lucidum* (fruit bodies)	[39]
**114**	Ganoderiol G (24,25,26-trihydroxy-7α-methoxy-5α-lanost-8-en-3-one)	*G.* *lucidum* (fruit bodies)	[39]
**115**	Ganoderiol H (3β, 24,25,26-tetrahydroxy-5α-lanost-8-en-7-one)	*G. lucidum (fruit bodies)*	[39]
**116**	Ganoderitriol M ((24*S*)-lanosta-7-oxo-8-en-3β, 24,25-triol)	*G.* *lucidum* (fruit bodies)	[58]
**117**	Sinensoic acid (3,26-dihydroxy-5-lanosta-8,24*E*-dien-21-oic acid)	*G.* *sinense* (fruit bodies)	[59]
**118**	Tsugarioside B (3α-acetoxy-5α-lanosta-8,24-diene-21-*O*-β-d-xyloside)	*G.* *tsugae* (fruit bodies)	[60]
**119**	Tsugaric acid A (3α-acetoxy-5α-lanosta-8,24-dien-21-oic acid)	*G. tsugae*	[61]
**120**	Ganosinoside A (3-oxo-5α-lanosta-8,24-dien-21-oic acid ester β-d-glucoside)	*G.* *sinense* (fruit bodies)	[47]
**121**	Tsugarioside A (3α-acetoxy-5α-lanosta-8,24-dien-21-oic acid ester β-d-glucoside)	*G.* *tsugae* (fruit bodies)	[60]
**122**	3-Oxo-5α-lanosta-8,24-dien-21-oic acid	*G.* *resinaceum* (fruit bodies)	[62]
**123**	3β-Hydroxy-5α-lanosta-8,24-dien-21-oic acid	*G. tsugae* (fruit bodies)	[60]

**Table:** 

Cpd	R1	R2	R3	R4
**99**	β-OH	β-OH	H	α-OH
**100**	=O	=O	H	=O
**101**	=O	=O	β-*O*-Ac	=O
**102**	β-OH	=O	β-*O*-Ac	=O
**103**	β-OH	β-OH	β-OH	=O
**104**	=O	β-OH	OH	=O
**105**	β-OH	=O	OH	=O

**Table:** 

Cpd	R1	R2	R3	R4	R5	R6	R7	R8
**106**	=O	=O	H	α-OH	β-CH_3_	ξ-OH	=O	COOH
**107**	β-OH	β-OH	H	=O	α-CH_3_	β-OH	ξ-OH	ξ-COOH
**108**	=O	β-OH	H	=O	α-CH_3_	β-OH	ξ-OH	ξ-COOH
**109**	β-OH	β-OH	β-OH	=O	β-CH_3_	β-OH	=O	COOH
**110**	β-OH	β-OH	=O	=O	α-CH_3_	ξ-OH	=O	COOH

**Table:** 

Cpd	R1	R2	R3	R4	R5
**111**	=O	=O	α-CH_3_	α-OH	CH_3_
**112**	=O	α-*O*-Et	β-CH_3_	ξ-OH	CH_2_OH
**113**	=O	=O	β-CH_3_	ξ-OH	CH_2_OH
**114**	=O	α-*O*-CH_3_	β-CH_3_	ξ-OH	CH_2_OH
**115**	β-OH	=O	β-CH_3_	ξ-OH	CH_2_OH
**116**	β-OH	=O	α-CH_3_	α-OH	CH_3_

**Table:** 

Cpd	R1	R2	R_3_
**117**	β-OH	α-COOH	CH_2_OH
**118**	α-*O*-Ac	CH_2_O-β-D-xylosyl	CH_3_
**119**	α-*O*-COCH_3_	α-COOH	CH_3_
**120**	=O	α-COO-β-D-glucopyranosyl	CH_3_
**121**	α-*O*-Ac	α-COOH	CH_3_
**122**	=O	α-COOH	CH_3_
**123**	β-OH	α-COOH	CH_3_

**Table 5:** *Ganoderma* triterpenes **124**–**147** in Figure 10, Figure 11, Figure 12, Figure 13, Figure 14, Figure 15, Figure 16 and Figure 17 and *Ganoderma* triterpenes **148**–**155** in Figure 18.

No.	Compound Name	Source	Ref.
**124**	3β, 7β-Dihydroxy-11,15,23-trioxolanost-8,16-dien-26-oic acid	*G.* *lucidum* (fruit bodies)	[63]
**125**	3β, 7β-Dihydroxy-11,15,23-trioxolanost-8,16-dien-26-oic acid methyl ester	*G.* *lucidum* (fruit bodies)	[63]
**126**	12β-Acetoxy-3β, 7β-dihydroxy-11,15,23-trioxolanost-8,16-dien-26-oic acid	*G.* *lucidum* (fruit bodies)	[63]
**127**	Methyl ganoderate I	*G.* *lucidum*	[20,22]
**128**	Methyl ganoderate AP (methyl 12β, l5α, 20-trihydroxy-3,7,11,23- tetraoxo-5α-lanost-8-en-26-oate)	*G.* *applanatum* (fruit bodies)	[52]
**129**	Methyl ganoderate N (Methyl 7β, 20-dihydroxy-3,11,15,23-tetraoxo-5α-lanost-8-en-26-oate)	*G.* *lucidum* (fruit bodies)	[64]
**130**	Methyl ganoderate M (methyl 7β, 12α-dihydroxy-3,11,15,23-tetraoxo-5α-lanost-8-en-26-oate)	*G.* *lucidum* (fruit bodies)	[64]
**131**	Ganoderiol E (3β, 26,27-trihydroxy-5α-lanosta-8,24-dien-7-one)	*G.* *lucidum* (fruit bodies)	[39]
**132**	Ganoderiol I (15α, 26,27-trihydroxy-5α-lanosta-8,24-dien-3-one)	*G.* *lucidum* (fruit bodies)	[39]
**133**	Ganoderiol J (26,27-dihydroxy-5α-lanosta-8,24-dien-3,7-dione)	*G. sinense* (fruit bodies)	[47]
**134**	Epoxyganoderiol A (24*S*, 25*S*-epoxy-7α, 26-dihydroxy-5α-lanost-8-en-3-one)	*G.* *lucidum*	[40]
**135**	Ganoderone C (5α-lanosta-8-ene-24,25-epoxy-26-hydroxy-3,7-dione)	*G. pfeifferi* (fruit bodies)	[42]
**136**	3-*O*-Acetylganoderic acid B (3β-acetoxy-7β-hydroxy-11,15,23-trioxolanost-8-en-26-oic acid)	*G.* *lucidum* (mycelia)	[65]
**137**	3-*O*-Acetylganoderic acid K (3β-acetyloxy-15α-hydroxy-7,11,23-trioxolanost-8-en-26-oic acid)	*G.* *lucidum* (mycelia)	[65]
**138**	Ethyl 3-*O*-acetylganoderate B	*G.* *lucidum* (mycelia)	[65]
**139**	Ethyl ganoderate J	*G.* *lucidum* (mycelia)	[65]
**140**	Applanoxidic acid G (15β, 20-dihydroxy-7α, 8α-epoxy-3,12,23-trioxo-5α-lanosta-9(11),16-dien-26-oic acid)	*G.* *applanatum*	[66]
**141**	Applanoxidic acid H (3β, 12α, 20-trihydroxy-7α, 8α-epoxydioxo-5α-lanosta-9(11),16-dien-26-oic acid)	*G. applanatum*	[66]
**142**	8β, 9α-Dihydroganoderic acid J	*G.* *lucidum* (fruit bodies)	[57]
**143**	Methyl 8β, 9α-dihydroganoderate J	*G.* *lucidum* (fruit bodies)	[57]
**144**	Ganosporeric acid A (3,7,11,12,15,23-hexaoxo-5α-lanosta-8-en-26-oic acid)	*G.* *lucidum* (spores)	[67]
**145**	24ξ-Methyl-5α-lanosta-25-one	*G.* *applanatum* (fruit bodies)	[68]
**146**	3α-Carboxyacetoxy-24-methylene-23-oxolanost-8-en-26-oic acid	*G.* *applanatum* (fruit bodies)	[69]
**147**	3α-Carboxyacetoxy-24-methyl-23-oxolanost-8-en-26-oic acid	*G. applanatum* (fruit bodies)	[69]
**148**	Fornicatin C ((3β)-3-hydroxy-18(13→12β)-*abeo-*lanosta-13(17),24-dien-18-oic acid)	*G.* *fornicatum* (fruit bodies)	[70]
**149**	3-Epipachymic acid (3α-acetoxy-16α-hydroxy-24-methylene-5α-lanost-8-en-21-oic acid)	*G. resinaceum* (fruit bodies)	[62]
**150**	3β, 15α-Diacetoxylanosta-8,24-dien-26-oic acid	*G.* *lucidum* (mycelia)	[71]
**151**	Tsugaric acid C ((24*R*,*S*)-3α-acetoxy-24-hydroxy-5α-lanosta-8,25-dien-21-oic acid)	*G. tsugae* (fruit bodies)	[60]
**152**	Ganoderic acid V1 ((24*E*)-3β, 20ξ-dihydroxy-7,11,15-trioxo-5α-lanosta-8,24-dien-26-oic acid)	*G.* *lucidum*	[72]
**153**	Tsugaric acid B (3α-acetoxy-16α-hydroxy-24ξ-methyl-5α-lanosta-8,25-dien-21-oic acid)	*G.* *tsugae*	[61]
**154**	Methyl ganoderenate E (7β, 12β-dihydroxy-3,11,15,23-tetraoxo-5α-lanosta-8,20*E*-dien-26-oate)	*G.* *lucidum* (fruit bodies)	[64]
**155**	8β, 9α-Dihydroganoderic acid C	*G.* *lucidum* (mycelia)	[65]

**Table:** 

Cpd	R1	R2
**124**	H	COOH
**125**	H	COOCH_3_
**126**	β-*O*-Ac	COOH

**Table:** 

Cpd	R1	R2	R3	R4
**127**	β-OH	β-OH	H	=O
**128**	=O	=O	β-OH	α-OH
**129**	=O	β-OH	H	=O
**130**	=O	β-OH	α-OH	=O

**Table:** 

Cpd	R1	R2	R3	R4
**131**	β-OH	=O	H	β-CH_3_
**132**	=O	β-CH_3_	α-OH	β-CH_3_
**133**	=O	=O	H	α-CH_3_

**Table:** 

Cpd	R1	R2
**134**	α-OH	β-CH_3_
**135**	=O	α-CH_3_

**Table:** 

Cpd	R1	R2	R3	R4
**136**	β-*O*-Ac	β-OH	=O	COOH
**137**	β-*O*-Ac	=O	α-OH	COOH
**138**	β-*O*-Ac	β-OH	=O	COOEt
**139**	=O	=O	α-OH	COOEt

**Table:** 

Cpd	R1	R2	R3
**140**	=O	=O	β-OH
**141**	β-OH	α-OH	=O

**Table:** 

Cpd	R1	R2	R3	R4	R5	R6	R7	R8	R9
**142**	=O	=O	=O	H	α-OH	β-CH_3_	=O	H	COOH
**143**	=O	=O	=O	H	α-OH	β-CH_3_	=O	H	COOCH_3_
**144**	=O	=O	=O	=O	=O	β-CH_3_	=O	H	COOH
**145**	H	H	H	H	H	α-CH_3_	H	ξ-CH_3_	=O

**Table:** 

Cpd	R
**146**	CH_2_
**147**	α-CH_3_

**Table 6:** *Ganoderma* triterpenes **156**–**196** in Figure 19.

No.	Compound Name	Source	Ref.
**156**	15-Hydroxy-ganoderic acid S (15α-hydroxy-3-oxo-5α-lanosta-7,9(11),24(*E*)-trien-26-oic acid)	*G.* *lucidum* (fruit bodies)	[35]
**157**	3α, 16α-Dihydroxylanosta-7,9(11),24-trien-21-oic acid	*G.* *applanatum* (fruit bodies)	[69]
**158**	3α, 16α, 26-Trihydroxylanosta-7,9(11),24-trien-21-oic acid	*G. applanatum* (fruit bodies)	[69]
**159**	Ganoderic acid S_1_	*G.* *lucidum* (fruit bodies)	[73]
**160**	Ganoderic acid SZ (3-oxo-lanosta-7,9(11),24(*Z*)-trien-26-oic acid)	*G.* *lucidum* (fruit bodies)	[74]
**161**	5α-Lanosta-7,9(11),24-triene-15α-26-dihydroxy-3-one	*G.* *concinna*	[75]
**162**	Ganoderic acid Me (3α, 15α-diacetoxy-5α-lanost-7,9(11),24*E*-trien-26-oic acid)	*G.* *lucidum* (cultured mycelial mat)	[41]
**163**	Ganoderic acid Mf (3α-acetoxy-15α-hydroxy-5α-lanost-7,9(11),24*E*-trien-26-oic acid)	*G. lucidum* (cultured mycelial mat)	[41]
**164**	Ganodermenonol (26-hydroxy-5α-lanosta-7,9(11),24-trien-3-one)	*G. lucidum* (dried fruit bodies)	[76]
**165**	Ganodermadiol (5α-lanosta-7,9(11),24-triene-3β, 26-diol)	*G. lucidum* (dried fruit bodies)	[76]
**166**	Ganodermatriol (5α-lanosta-7,9(11),24-triene-3β, 26,27-triol)	*G. lucidum* (fruit bodies)	[77]
**167**	Ganodermic acid S (lanosta-7,9(11),24-trien-3β, 15α-diacetoxy-26-oic acid)	G. *lucidum*	[78]
**168**	Carnosodione (26,27-dihydroxylanosta-7,9(11),24-trien-3,16-dione)	G. carnosum (fruit bodies)	[79]
**169**	Canoderol B ((24*E*)-5α-lanosta-7,9(11),24-trien-3,26-diol)	*G.* *lucidum*	[23]
**170**	Ganoderic acid Mk (3α, 22-diacetoxy-15α-hydroxy-5α-lanost-7,9(11),24*E*-trien-26-oic acid)	*G.* *lucidum* (mycelia mat)	[43]
**171**	Ganoderiol B (15α, 26,27-trihydroxy-5α-lanosta-7,9(11),24-trien-3-one)	*G.* *lucidum* (fruit bodies)	[77]
**172**	Ganoderic acid T ((22*S*, 24*E*)-3α, 15α, 22-triacetoxy-5α-lanosta-7,9,(11),24-trien-26-oic acid)	*G.* *lucidum* (cultured mycelia)	[80]
**173**	Ganoderic acid S ((22*S*, 24*E*)-22-acetoxy-3α-hydroxy-5α-lanosta-7,9(11),24-trien-26-oic acid)	*G.* *lucidum* (cultured mycelia)	[23,80]
**174**	Ganoderic acid R ((22*S*, 24*E*)-3α, 22-diacetoxy-5α-lanosta-7,9,(11),24-trien-26-oic acid)	*G.* *lucidum* (cultured mycelia)	[80]
**175**	Ganorbiformin G	*G.* *orbiforme*	[46]
**176**	Lanosta-7,9(11),24-trien-3β, 15α, 22β-triacetoxy-26-oic acid	*G.* *lucidum*	[81]
**177**	Lanosta-7,9(11),24-trien-15α-acetoxy-3α-hydroxy-23-oxo-26-oic acid	*G.* *lucidum*	[81]
**178**	Lanosta-7,9(11),24-trien-3α, l5α-diacetoxy-23-oxo-26-oic acid	*G.* *lucidum*	[81]
**179**	Lanosta-7,9(11),24-trien-3α, 15α-hydroxy-23-oxo-26-oic acid	*G.* *lucidum*	[81]
**180**	Lanosta-7,9(11),24-trien-3α-acetoxy-15α, 22β-dihydroxy-26-oic acid	*G.* *lucidum*	[81]
**181**	Ganodermic acid T-N (3β-hydroxy-15α-acetoxy-lanosta-7,9(11),24-trien-26-oic acid)	*G.* *lucidum* (mycelia)	[82]
**182**	Ganodermic acid T-O (3β-acetoxy-15α-hydroxy-lanosta-7,9(11),24-trien-26-oic acid)	*G.* *lucidum* (mycelia)	[82]
**183**	Ganodermic acid T-Q (3β-oxo-15α-acetoxy-lanosta-7,9(11),24-trien-26-oic acid)	*G.* *lucidum* (mycelia)	[82]
**184**	Compound 10	*G.* *orbiforme*	[46]
**185**	Ganoderic acid P ((22*S*, 24*E*)-15α, 22-diacetoxy-3α-hydroxy-5α-lanosta-7,9(11),24-trien-26-oic acid)	*G.* *lucidum* (cultured mycelium)	[50]
**186**	Ganoderic acid Q ((22*S*, 24*E*)-3α, 22-diacetoxy-15α-hydroxy-5α-lanosta-7,9(11),24-trien-26-oic acid)	*G.* *lucidum* (cultured mycelium)	[50]
**187**	Ganoderic acid Jc (15α, 23-dihydroxy-3-oxo-5α-lanosta-7,9(11),24-trien-26-oic acid)	*G. sinense* (fruit bodies)	[47]
**188**	Ganodermatetraol (3β, 15α, 26,27-tetrahydroxy-5α-lanosta-7,9(11),24-triene)	*G.* *sinense* (fruit bodies)	[47]
**189**	5α-Lanosta-7,9(11),24-triene-3β-hydroxy-26-al	*G.* *concinna*	[75]
**190**	Ganoderiol F (26,27-dihydroxy-5α-lanosta-7,9(11),24-trien-3-one)	*G. lucidum* (fruit bodies)	[39]
**191**	26,27-Dihydroxy-5α-lanosta-7,9(11),24-triene-3,22-dione	*G. lucidum* (basidiocarp)	[83]
**192**	26-Hydroxy-5α-lanosta-7,9(11),24-triene-3,22-dione	*G. lucidum* (basidiocarp)	[83]
**193**	Ganodermic acid P1 (lanosta-7,9(11),24-trien-3α, 22β-diacetoxy-15α-hydroxy-26-oic acid)	*G. lucidum* (mycelia)	[84]
**194**	Ganodermic acid P_2_ (lanosta-7,9(11),24-trien-15α, 22β-diacetoxy-3β-hydroxy-26-oic acid)	*G.* *lucidum* (mycelia)	[84]
**195**	Lanosta-7,9(11),24-trien-3β, 15α, 22-triacetoxy-26-oic acid	*G.* *amboinense* (fruit bodies)	[85]
**196**	16α-Hydroxy-3-oxolanosta-7,9(11),24-trien-21-oic acid	*G.* *applanatum* (fruit bodies)	[69]

**Table 7:** *Ganoderma* triterpenes (**197**–**213**) in Figure 20.

No.	Compound Name	Source	Ref.
**197**	Lucialdehyde A ((24*E*)-3β-hydroxy-5α-lanosta-7,9(11),24-trien-26-al)	*G.* *lucidum* (fruit bodies)	[25]
**198**	Ganoderiol a triacetate (3β, 24,26-triacetoxy-5α-lanosta-7, 9(11)-dien-25-ol)	*G.* *sinense* (fruit bodies)	[86]
**199**	Ganoderal A	*G.* *lucidum*	[23]
**200**	Ganoderol A	*G.* *lucidum*	[23]
**201**	Lucidumol B ((24*S*)-lanosta-7,9(11)-diene-3β, 24,25-triol)	*G.* *lucidum* (spores)	[38]
**202**	Ganodermanontiol (24,25,26-trihydroxy-5α-lanosta-7,9(11)-dien-3-one)	*G.* *lucidum* (spores)	[67]
**203**	Ganoderiol A (5α-lanosta-7,9(11)-dien-3β, 24,25,26-tetraol)	*G.* *lucidum* (fruit bodies)	[77]
**204**	Ganodermanondiol	*G.* *lucidum* (fruit bodies)	[87]
**205**	Ganoderic acid X (3α-hydroxy-15α-acetoxy-lanosta-7,9(11),24-trien-26-oic acid)	*G.* *amboinense*	[88]
**206**	Ganoderic acid TR	*G.* *lucidum*	[89]
**207**	Ganodermic acid Ja (lanosta-7,9(11),24-trien-3α, 15α-dihydroxy-26-oic acid)	*G.* *lucidum* (mycelia)	[84]
**208**	Ganodermic acid Jb (lanosta-7,9(11),24-trien-3β, 15α-dihydroxy-26-oic acid)	*G.* *lucidum* (mycelia)	[84]
**209**	Ganodermic acid R (lanosta-7,9(11),24-trien-3α, 15α-diacetoxy-26-oic acid)	*G.* *lucidum*	[78]
**211**	15α-Hydroxy-3-oxo-5α-lanosta-7,9,24(*E*)-triene-26-oic acid	*G.* *lucidum*	[26]
**212**	15α, 26-Dihydroxy-5α-lanosta-7,9,24(*E*)-trien-3-one	*G.* *lucidum*	[26]
**213**	3β-Hydroxy-5α-lanosta-7,9,24(*E*)-trien-26-oic acid	*G.* *lucidum*	[26]

**Table:** 

Cpd	R1	R2	R3	R4	R5	R6	R7	R8	
**156**	=O	α-OH	H	β-CH_3_	H	H	COOH	CH_3_	
**157**	α-OH	H	α-OH	α-COOH	H	H	CH_3_	CH_3_	
**158**	α-OH	H	α-OH	α-COOH	H	H	CH_2_OH	CH_3_	
**159**	=O	H	H	α-CH_3_	H	H	COOH	CH_3_	
**160**	=O	H	H	β-CH_3_	H	H	COOH	CH_3_	
**161**	=O	α-OH	H	α-CH_3_	H	H	CH_2_OH	CH_3_	
**162**	α-*O*-Ac	α-*O*-Ac	H	β-CH_3_	ξ-H_2_	H	COOH	CH_3_	
**163**	β-*O*-Ac	α-OH	H	β-CH_3_	ξ-H_2_	H	COOH	CH_3_	
**164**	=O	H	H	α-CH_3_	H	H	CH_2_OH	CH_3_	
**165**	β-OH	H	H	α-CH_3_	H	H	CH_2_OH	CH_3_
**166**	β-OH	H	H	α-CH_3_	H	H	CH_2_OH	CH_2_OH
**167**	β-*O*-Ac	α-*O*-Ac	H	β-CH_3_	H	H	COOH	CH_3_
**168**	=O	H	O	β-CH_3_	H	H	CH_2_OH	CH_2_OH
**169**	β-OH	H	H	β-CH_3_	H	H	CH_2_OH	CH_3_
**170**	α-*O*-Ac	α-OH	H	β-CH_3_	ξ-*O*-Ac	H	COOH	CH_3_
**171**	=O	α-OH	H	α-CH_3_	H	H	CH_2_OH	CH_2_OH
**172**	α-*O*-Ac	α-*O*-Ac	H	α-CH_3_	β-*O*-Ac	H	COOH	CH_3_
**173**	α-OH	H	H	α-CH_3_	β-*O*-Ac	H	COOH	CH_3_
**174**	α-*O*-Ac	H	H	α-CH_3_	β-*O*-Ac	H	COOH	CH_3_
**175**	=O	H	H	α-CH_3_	β-*O*-Ac	H	COOH	CH_3_
**176**	β-*O*-Ac	α-*O*-Ac	H	β-CH_3_	β-*O*-Ac	H	COOH	CH_3_
**177**	α-OH	α-*O*-Ac	H	β-CH_3_	H	=O	COOH	CH_3_
**178**	α-*O*-Ac	α-*O*-Ac	H	β-CH_3_	H	=O	COOH	CH_3_
**179**	α-*O*-Ac	α-OH	H	β-CH_3_	H	=O	COOH	CH_3_
**180**	α-*O*-Ac	α-OH	H	β-CH_3_	H	H	COOH	CH_3_
**181**	β-OH	α-*O*-Ac	H	β-CH_3_	H	H	COOH	CH_3_
**182**	β-*O*-Ac	α-OH	H	β-CH_3_	H	H	COOH	CH_3_
**183**	=O	α-*O*-Ac	H	β-CH_3_	H	H	COOH	CH_3_
**184**	=O	α-*O*-Ac	H	α-CH_3_	β-*O*-Ac	H	COOH	CH_3_
**185**	α-OH	α-*O*-Ac	H	α-CH_3_	β-*O*-Ac	H	COOH	CH_3_
**186**	α-*O*-Ac	α-OH	H	α-CH_3_	β-*O*-Ac	H	COOH	CH_3_
**187**	=O	α-OH	H	α-CH_3_	H	OH	COOH	CH_3_
**188**	β-OH	α-OH	H	α-CH_3_	H	H	CH_2_OH	CH_2_OH
**189**	β-OH	H	H	α-CH_3_	H	H	CHO	CH_3_
**190**	=O	H	H	β-CH_3_	H	H	CH_2_OH	CH_2_OH
**191**	=O	H	H	α-CH_3_	=O	H	CH_2_OH	CH_2_OH
**192**	=O	H	H	α-CH_3_	=O	H	CH_2_OH	CH_3_
**193**	α-*O*-Ac	α-OH	H	β-CH_3_	*O*-Ac	H	COOH	CH_3_
**194**	β-OH	α-*O*-Ac	H	β-CH_3_	*O*-Ac	H	COOH	CH_3_
**195**	β-*O*-Ac	α-*O*-Ac	H	α-CH_3_	β-*O*-Ac	H	COOH	CH_3_
**196**	=O	H	α-OH	α-COOH	H	H	CH_3_	CH_3_

**Table:** 

Cpd	R1	R2	R3	R4	R5	R6
**197**	β-OH	H	α-CH_3_	△^24,25^	△^24,25^	CHO
**198**	β-*O*-Ac	H	α-CH_3_	OAc	OH	CH_2_-*O*-Ac
**199**	=O	H	β-CH_3_	△^24,25^	△^24,25^	CHO
**200**	=O	H	β-CH_3_	△^24,25^	△^24,25^	CH_2_OH
**201**	β-OH	H	α-CH_3_	α-OH	OH	CH_3_
**202**	=O	H	β-CH_3_	α-OH	H	CH_2_OH
**203**	β-OH	H	α-CH_3_	OH	OH	CH_2_OH
**204**	=O	H	α-CH_3_	OH	OH	CH_3_
**205**	β-OH	α-*O*-Ac	β-CH_3_	H	H	COOH
**206**	=O	α-OH	α-CH_3_	△^24,25^	△^24,25^	COOH
**207**	α-OH	α-OH	β-CH_3_	△^24,25^	△^24,25^	COOH
**208**	β-OH	α-OH	β-CH_3_	△^24,25^	△^24,25^	COOH
**209**	α-*O*-Ac	α-*O*-Ac	β-CH_3_	△^24,25^	△^24,25^	COOH
**210**	=O	H	α-CH_3_	OH	OH	CH_2_OH
**211**	=O	α-OH	α-CH_3_	△^24,25^	△^24,25^	COOH
**212**	=O	α-OH	α-CH_3_	△^24,25^	△^24,25^	CH_2_OH
**213**	β-OH	H	α-CH_3_	△^24,25^	△^24,25^	COOH

**Table 8:** *Ganoderma* triterpenes (**214**–**221**) in Figure 21 and Figure 22.

No.	Compound Name	Source	Ref.
**214**	3α, 15α, 22α-Trihydroxylanosta-7,9(11),24-trien-26-oic acid	*G.* *lucidum* (mycelia)	[71]
**215**	3β, 15α, 22β-Trihydroxylanosta-7,9(11),24-trien-26-oic acid	*G.* *lucidum* (mycelia)	[71]
**216**	3α, 15α-Diacetoxy-22α-hydroxylanosta-7,9(11),24-trien-26-oic acid	*G.* *lucidum* (mycelia)	[71]
**217**	3β, 15α-Diacetoxy-22α-hydroxylanosta-7,9(11),24-trien-26-oic acid	*G.* *lucidum* (mycelia)	[71]
**218**	22β-Acetoxy-3α, 15α-dihydroxylanosta-7,9(11),24-trien-26-oic acid	*G.* *lucidum* (mycelia)	[71]
**219**	22β-Acetoxy-3β, 15α-dihydroxylanosta-7,9(11),24-trien-26-oic acid	*G.* *lucidum* (mycelia)	[71]
**220**	Epoxyganoderiol B (24*S*, 25*S*-epoxy-26-hydroxy-5α-lanosta-7,9(11)-diene-3-one)	*G.* *lucidum*	[40]
**221**	Epoxyganoderiol C (24*S*, 25*S*-epoxy-5α-lanosta-7,9(11)-diene-3β, 26-diol)	*G.* *lucidum*	[40]

**Table:** 

Cpd	R1	R2	R3
**214**	α-OH	α-OH	α-OH
**215**	β-OH	α-OH	β-OH
**216**	α-*O*-Ac	α-*O*-Ac	α-OH
**217**	β-*O*-Ac	α-*O*-Ac	α-OH
**218**	α-OH	α-OH	β-*O*-Ac
**219**	β-OH	α-OH	β-*O*-Ac

**Table:** 

Cpd	R1
**220**	β-OH
**221**	=O

**Table 9:** *Ganoderma* triterpenes (**222**–**260**) in Figure 23.

No.	Compound Name	Source	Ref.
**222**	Butyl lucidenate N	*G.* *lucidum* (fruit bodies)	[17]
**223**	Butyl lucidenate A	*G.* *lucidum* (fruit bodies)	[17]
**224**	20(21)-Dehydrolucidenic acid N (3β, 7β-dihydroxy-11,15-dioxo-25,26,27- trinorlanosta-8,20-dien-24-oic acid)	*G.* *sinense* (fruit bodies)	[33]
**225**	20-Hydroxylucidenic acid A (7β, 20ξ-dihydroxy-3,11,15-trioxo-25,26,27-trinorlanost-8-en-24-oic acid)	*G. sinense* (fruit bodies)	[33]
**226**	Methyl lucidenate D (methyl 12β-acetoxy-3,7,11,15-tetraoxo-5α-lanost-8-en-24-oate)	*G.* *lucidum* (fruit bodies)	[53,54]
**227**	20(21)-Dehydrolucidenic acid A (7β-Hydroxy-3,11,15-trioxo-25,26,27-trisnorlanosta-8,20(21)-dien-24-oic acid)	*G.* *lucidum* (fruit bodies)	[90]
**228**	Methyl 20(21)-dehydrolucidenate A (methyl 7β-hydroxy-3,11,15-trioxo-25,26,27-trisnorlanosta-8,20(21)-dien-24-oate)	*G.* *lucidum* (fruit bodies)	[90]
**229**	Lucidenic acid N (3,7-dihydroxy-4,4,14-trimethyl-11,15-dioxo-5-chol-8-en-24-oic acid)	*G.* *lucidum* (dried fruit bodies)	[91,92]
**230**	Lucidenic acid D (12β-acetoxy-4,4,14α-trimethyl-3,7,11,15-tetraoxo-5α-chol-8-en-24-oic acid)	*G.* *lucidum* (dried fruit bodies)	[31]
**231**	Methyl lucidenate E	*G.* *lucidum* (gills)	[54]
**232**	Methyl lucidenate F	*G.* *lucidum* (gills)	[23,54]
**233**	Ethyl lucidenates A (ethyl 7β-hydroxy-4,4,14α-trimethyl-3,11,15-trioxo-5α-chol-8-en-24-oate)	*G.* *lucidum* (fruit bodies)	[93]
**234**	3β-Oxo-formyl-7β, 12β-dihydroxy-4,4,14α-trimethyl-5α-chol-11,15-dioxo-8-en(*E*)-24-oic acid	*G.* *lucidum*	[24]
**235**	Lucidenic acid A (7β-hydroxy-4,4,14α-trimethyl-3,11,15-trioxo-5α-chol-8-en-24-oic acid)	*G.* *lucidum* (dried fruit bodies)	[94]
**236**	Lucidenic acid B (7β, 12-dihydroxy-4,4,14α-trimethyl-3,11,15-trioxo-5α-chol-8-en-24-oic acid)	*G.* *lucidum* (dried fruit bodies)	[94]
**237**	Lucidenic acid C (3β, 7β, 12-trihydroxy-4,4,14α-trimethyl-11,15-dioxo-5α-chol-8-en-24-oic acid)	*G.* *lucidum* (dried fruit bodies)	[94]
**238**	4,4,14α-Trimethyl-3,7-dioxo-5α-chol-8-en-24-oic acid	*G.* *lucidum*	[26]
**239**	Lucidenic acid P (3β, 7β-dihydroxy-12β-acetoxy-25,26,27-trinor-11,15-dioxo dioxolanost-8-en-24-oic acid)	*G.* *lucidum* (fruit bodies)	[95]
**240**	Methyl lucidenate P	*G.* *lucidum* (fruit bodies)	[95]
**241**	Methyl lucidenate Q (methyl-7β, 15α-dihydroxy-25,26,27-trinor-3,11-dioxolanost-8-en-24-oate)	*G.* *lucidum* (fruit bodies)	[95]
**242**	3β-Hydroxy-4,4,14-trimethyl-7,11,15-trioxochol-8-en-24-oic acid	*G.* *lucidum* (fruit bodies)	[28]
**243**	Methyl lucidenate D_2_	*G.* *lucidum* (gill surface)	[30]
**244**	Methyl lucidenate E_2_	*G.* *lucidum* (gill surface)	[30]
**248**	Methyl lucidenate N (methyl 3β, 7β-dihydroxy-4,4,14α-trimethyl-11,15-dioxo-5α-chol-8-en-24-oate)	*G.* *lucidum* (fruit bodies)	[96]
**249**	*t*-Butyl lucidenate B (*t*-butyl 7β, 12β-dihydroxy-4,4,14α-trimethyl-3,11,15-trioxo-5α-chol-8-en-24-oate)	*G.lucidum* (fruit bodies)	[96]
**250**	Methyl lucidenate A	*G.* *lucidum* (fruit bodies)	[93]
**251**	Lucidenic acid D_2_	*G.* *lucidum* (fruit bodies)	[95]
**252**	20-Hydroxylucidenic acid D_2_ ((20ξ)-12β-acetoxy-20-hydroxy-3,7,11,15-tet-raoxo-25,26,27-trisnorlanost-8-en-24-oic acid)	*G.* *lucidum* (fruit bodies)	[90]
**253**	20-Hydroxylucidenic acid F ((20ξ)-20-hydroxy-3,7,11,15-tetraoxo-25,26,27-trisnorlanost-8-en-24-oic acid)	*G.* *lucidum* (fruit bodies)	[90]
**254**	20-Hydroxylucidenic acid E_2_ (12β-acetoxy-3β-hydroxy-7,11,15-trioxo-25,26,27-trisnorlanost-8-en-24-oic acid)	*G.* *lucidum* (fruit bodies)	[90]
**255**	20-Hydroxylucidenic acid N ((20ξ)-3β, 7β, 20-trihydroxy-11,15-dioxo-25,26,27-trisnorlanost-8-en-24-oic acid)	*G.* *lucidum* (fruit bodies)	[90]
**256**	20-Hydroxylucidenic acid P ((20ξ)-12β-acetoxy-3β, 7β, 20-trihydroxy-11,15-dioxo-25,26,27-trisnorlanost-8-en-24-oic acid)	*G.* *lucidum* (fruit bodies)	[90]
**257**	Lucidenic acid F	*G.* *lucidum* (gills)	[22]
**258**	Methyl lucidenate C	*G.* *lucidum*	[26]
**259**	Lucidenic acid E_2_	*G.* *lucidum* (fruit bodies)	[95]
**260**	Lucideric acid A	*G.* *lucidum*	[26]

**Table:** 

Cpd	R1	R2	R3	R4	R5	R6	R7	R8
**222**	β-OH	β-OH	=O	H	=O	α-CH_3_	H	COOBu
**223**	=O	β-OH	=O	H	=O	α-CH_3_	H	COOBu
**224**	β-OH	β-OH	=O	H	=O	CH_2_	△^20,21^	COOH
**225**	=O	β-OH	=O	H	=O	β-CH_3_	ξ-OH	COOH
**226**	=O	=O	=O	β-*O*-Ac	=O	β-CH_3_	H	COOCH_3_
**227**	=O	β-OH	=O	H	=O	CH_2_	△^20,21^	COOH
**228**	=O	β-OH	=O	H	=O	CH_2_	△^20,21^	COOCH_3_
**229**	β-OH	β-OH	=O	H	=O	α-CH_3_	H	COOH
**230**	=O	=O	=O	β-*O*-Ac	=O	β-CH_3_	H	COOH
**231**	β-OH	=O	=O	β-*O*-Ac	=O	β-CH_3_	H	COOCH_3_
**232**	=O	=O	=O	H	=O	β-CH_3_	H	COOCH_3_
**233**	=O	OH	=O	H	=O	α-CH_3_	H	COOEt
**234**	β-*O*-CHO	β-OH	=O	OH	=O	β-CH_3_	H	COOH
**235**	=O	β-OH	=O	H	=O	β-CH_3_	H	COOH
**236**	=O	β-OH	=O	β-OH	=O	β-CH_3_	H	COOH
**237**	β-OH	β-OH	=O	β-OH	=O	β-CH_3_	H	COOH
**238**	=O	=O	H	H	H	α-CH_3_	H	COOH
**239**	β-OH	β-OH	=O	β-*O*-Ac	=O	α-CH_3_	H	COOH
**240**	β-OH	β-OH	=O	β-*O*-Ac	=O	α-CH_3_	H	COOCH_3_
**241**	=O	β-OH	=O	H	α-OH	α-CH_3_	H	COOCH_3_
**242**	β-OH	=O	=O	H	=O	α-CH_3_	H	COOH
**243**	=O	=O	=O	*O*-Ac	=O	β-CH_3_	H	COOCH_3_
**244**	β-OH	=O	=O	*O*-Ac	=O	β-CH_3_	H	COOCH_3_
**245**	=O	=O	=O	α-OH	=O	β-CH_3_	H	COOCH_3_
**246**	β-OH	=O	=O	β-OH	=O	β-CH_3_	H	COOCH_3_
**247**	β-OH	α-OH	=O	H	α-OH	β-CH_3_	H	COOCH_3_
**248**	β-OH	β-OH	=O	H	=O	α-CH_3_	H	COOCH_3_
**249**	=O	β-OH	=O	β-OH	=O	α-CH_3_	H	COOBu
**250**	=O	β-OH	=O	H	=O	α-CH_3_	H	COOCH_3_
**251**	=O	=O	=O	β-*O*-Ac	=O	α-CH_3_	H	COOH
**252**	=O	=O	=O	β-*O*-Ac	=O	β-CH_3_	ξ-OH	COOH
**253**	=O	=O	=O	H	=O	β-CH_3_	ξ-OH	COOH
**254**	β-OH	=O	=O	β-*O*-Ac	=O	β-CH_3_	ξ-OH	COOH
**255**	β-OH	β-OH	=O	H	=O	β-CH_3_	ξ-OH	COOH
**256**	β-OH	β-OH	=O	β-*O*-Ac	=O	β-CH_3_	ξ-OH	COOH
**257**	=O	=O	=O	H	=O	α-CH_3_	H	COOH
**258**	β-OH	β-OH	=O	H	=O	α-CH_3_	H	COOCH_3_
**259**	β-OH	=O	=O	β-*O*-Ac	=O	α-CH_3_	H	COOH
**260**	=O	β-OH	=O	H	=O	α-CH_3_	H	COOH

**Table 10:** *Ganoderma* triterpenes **261**–**280** in Figure 24, Figure 25, Figure 26, Figure 27, Figure 28, Figure 29, Figure 30 and Figure 31.

No.	Compound Name	Source	Ref.
**261**	4,4,14α-Trimethyl-5α-chol-7,9(11)-dien-3-oxo-24-oic acid	*G.* *lucidum* (dried fruit bodies)	[73]
**262**	Ganoderic acid Jd (15α-hydroxy-3-oxo-5α-lano-sta-7,9(11)-dien-24-oic acid)	*G.* *sinense* (fruit bodies)	[47]
**263**	Methyl lucidenate H (methyl 3β, 7β-dihydroxy-4α-hydroxymethyl-4β, 14α-dimethyl-11,15-dioxo-5α-chol-8-en-24-oate)	*G.* *lucidum* (fruit bodies)	[64]
**264**	Methyl lucidenate I (3β-hydroxy-4α-hydroxymethyl-4β, 14α-dimethyl-7,11,15-trioxo-5α-chol-8-en-24-oate)	*G.* *lucidum* (fruit bodies)	[64]
**265**	Methyl lucidenate J (3β, 12β-dihydroxy-4α-hydroxymethyl-4β, 14α-dimethyl-7,11,15-trioxo-5α-chol-8-en-24-oate)	*G.* *lucidum* (fruit bodies)	[64]
**266**	Methyl lucidenate Ha	*G.* *sinense* (fruit bodies)	[47]
**267**	Colossolactone I ((22S)-3-β-hydroxylanosta-8,24-dien-26,22-olide)	*G.* *colossum*	[97]
**268**	Colossolactone II ((22S)-1,3-β-dihydroxylanosta-8,24-dien-26,22-olide)	*G.* *colossum*	[97]
**269**	Colossolactone D	*G.* *colossum* (fruit bodies)	[98]
**270**	Colossolactone E	*G.* *colossum* (fruit bodies)	[98]
**271**	Colossolactone F	*G.* *colossum* (fruit bodies)	[98]
**272**	Colossolactone G	*G.* *colossum* (fruit bodies)	[98]
**273**	Ganosporelactone A	*G.* *lucidum* (spores)	[99]
**274**	Ganosporelactone B	*G.* *lucidum* (spores)	[99]
**275**	Ganosinensin B (ganodermanontriol 24-*O*-{(2*Z*, 5*E*, 9*E*)-2-[2-(2,5-dihydroxyphenyl)-2-oxo-ethylidene]-11-hydroxy-6,10-dimethylundeca-5,9-dienate)	*G.* *sinense* (fruit bodies)	[100]
**276**	Ganosinensin C (ganodermanontriol 24-*O*-{(2*Z*, 5*E*, 9*E*)-2-[2-(2,5-dihydroxyphenyl)ethylidene]-11-hydroxy-6,10-dimethylundeca-5,9- dien-ate)	*G.* *sinense* (fruit bodies)	[100]
**277**	Ganodermacetal (methyl 7β, 15α-isopropylide-nedioxy-3β-hydroxy-11,23-dioxo-5α-lanost-8-en-26-oate)	*G.* *amboinense* (fruit bodies)	[85]
**278**	Methyl ganoderate A acetonide (methyl 7β, 15α-isopropylidenedioxy-3,11,23-trioxo-5α-lanost-8-en-26-oate)	*G.* *lucidum* (fruit bodies)	[16]
**279**	Applanoxidic acid A (15α-hydroxy-7α, 8α-epoxy-3,12,23-trioxo-5α-lanosta-9(11),20*E*-dien-26-oic acid)	*G.* *applanatum*	[101]
**280**	Applanoxidic acid B (3β-hydroxy-7α, 8α-epoxy-12,15,23-trioxo-5α-lanosta-9(11),20*E*-dien-26-oic acid)	*G.* *applanatum*	[101]

**Table:** 

Cpd	R1	R2
**261**	=O	H
**262**	=O	α-OH

**Table:** 

Cpd	R1	R2	R3
**263**	β-OH	H	β-CH3
**264**	=O	H	β-CH3
**265**	=O	β-OH	β-CH3
**266**	β-OH	H	α-CH3

**Figure 26:**
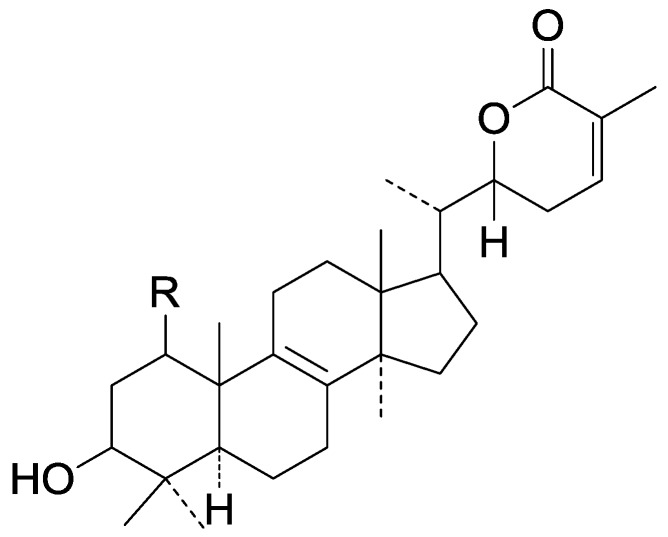
Structures of compounds **267**–**268**.

**Table:** 

Cpd	R
**267**	H
**268**	β-OH

**Figure 27:**
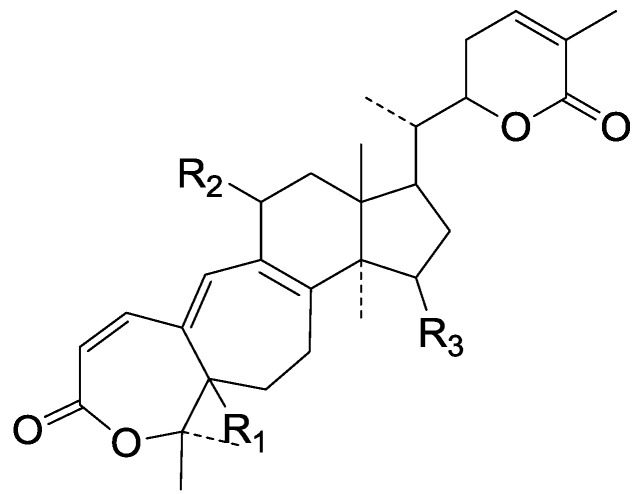
Structures of compounds **269**–**272**.

**Table:** 

Cpd	R1	R2	R3
**269**	α-H	β-H	β-OH
**270**	α-H	β-H	β-*O*-Ac
**271**	α-H	β-OH	β-*O*-Ac
**272**	ξ-OH	H	β-*O*-COCH_3_

**Figure 28:**
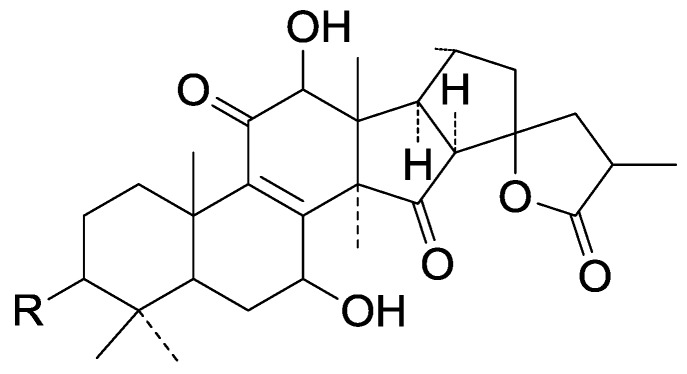
Structures of compounds **273**–**274**.

**Table:** 

Cpd	R
**273**	=O
**274**	β-OH

**Figure 29:**
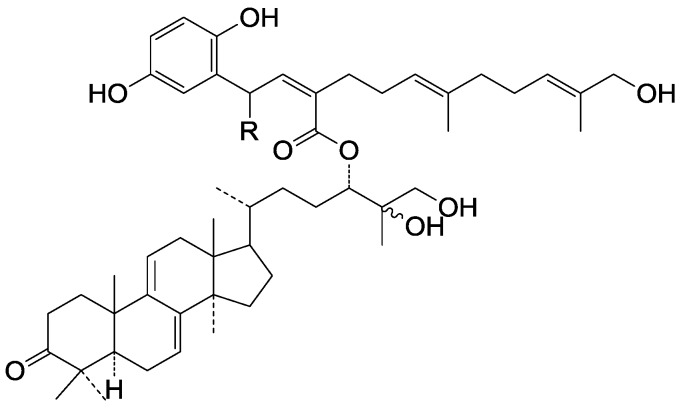
Structures of compounds **275**–**276**.

**Table:** 

Cpd	R
**275**	=O
**276**	H

**Figure 30:**
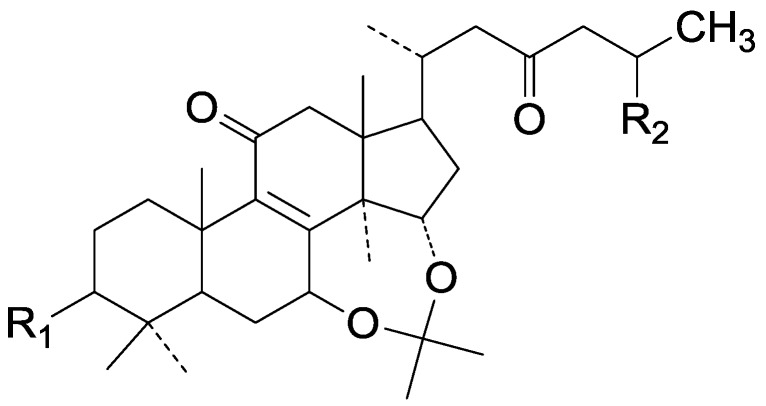
Structures of compounds **277**–**278**.

**Table:** 

Cpd	R1	R2
**277**	β-OH	COOH
**278**	=O	COOCH_3_

**Figure 31:**
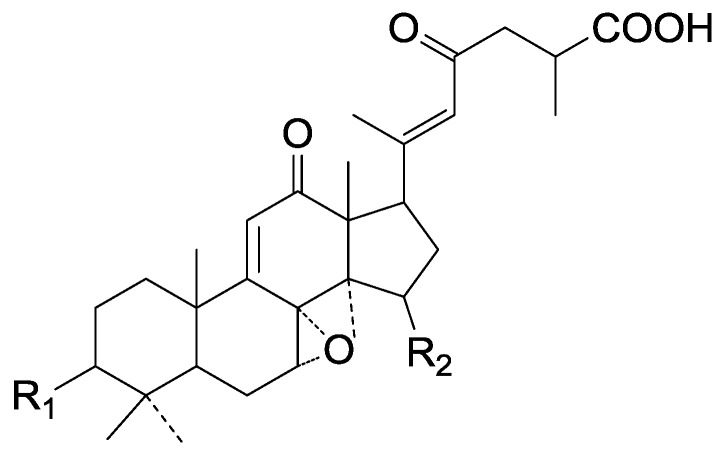
Structures of compounds **279**–**280**.

**Table:** 

Cpd	R1	R2
**279**	=O	α-OH
**280**	β-OH	=O

**Table 11:** *Ganoderma* triterpenes **281**–**287**, **288**–**307**, **308**–**311**, **312**, **313**–**315**, **316** in Figure 32, Figure 33, Figure 34, Figure 35, Figure 36, Figure 37, Figure 38, Figure 39 and Figure 40.

No.	Compound Name	Source	Ref.
**281**	Applanoxidic acid C (20-hydroxy-7α, 8α-epoxy-3,12,15,23-tetraoxo-5α-lanosta-9(11),16-dien-26-oic acid)	*G.* *applanatum*	[101]
**282**	Applanoxidic acid D (3β, 20-dihydroxy-7α, 8α-epoxy-12,15,23-trioxo-5α-lanosta-9(11),16-dien-26-oic acid)	*G. applanatum*	[101]
**283**	Lanosta-7,9(11),24-trien-3-one15,26-dihydroxy	*G. zonatum* *Murill.*	[27]
**284**	Lanosta-7,9(11),24-trien-26-oic,3-hydroxy	*G. zonatum* *Murill.*	[27]
**285**	Ganoderic acid Y ((24*E*)-3-ol-5α-lanosta-7,9(11),24-trien-26-oic acid)	*G. zonatum* *Murill.*	[27]
**286**	Applanoxidic acid E (15β-hydroxy-7α, 8α-epoxy-3,12,23-trioxo-5α-lanosta-9(11),20*E*-dien-26-oic acid)	*G. applanatum*	[66]
**287**	Applanoxidic acid F (7α, 8α-epoxy-3,12,15,23-tetraoxo-5α-lanosta-9(11),20*E*-dien-26-oic acid)	*G. applanatum*	[66]
**288**	Ganosinensin A (ganodermanontriol 26-*O*-{(2*Z*, 5*E*, 9*E*)-2-[2-(2,5-dihydroxyphenyl)-2-oxo-ethylidene]-11-hydroxy-6,10-dimethylundeca-5,9-dienate})	*G. sinense* (fruit bodies)	[100]
**289**	Colossolactone III ((22*S*)-3β, 19-epoxy-lanosta-8,24-dien-26,22-olide)	*G. colossum*	[97]
**290**	Colossolactone IV ((22*S*)-A,B-dihomo-19-nor-4-oxalanosta-8,24-dien-26,22-olide)	*G. colossum*	[97]
**291**	Colossolactone VIII ((22*S*, 23*R*)-A,B-dihomo-19-nor-15-β-acetoxy-23-hydroxy-4-oxa-3-oxolanosta-1,8,19,24-tetraen-26,22-olide)	*G. colossum*	[102]
**292**	Austrolactone ((23*S*, 25*S*)-12α, 23-epoxy-3β, 15β, 20α-trihydroxy-7,11-dioxo-5α-lanosta-8,16-dien-23,26-olide)	*G. australe*	[103]
**293**	Ganolactone B (3β, 7β-dihydroxy-11,15-dioxolanosta-8-en-24→20 lactone)	*G.* *sinense* (fruit bodies)	[86]
**294**	Ganolactone (7β-hydroxy-3,11,15-trioxo-lanosta-8-en-24→20s lactone)	*G.* *lucidum* (fruit bodies)	[104]
**295**	Colossolactone B	*G.* *colossum* (fruit bodies)	[98]
**296**	Colossolactone C	*G.* *colossum* (fruit bodies)	[98]
**297**	3α-(3-Hydroxy-5-methoxy-3-methyl-1,5-dioxopentyloxy)-24-methylene-5α-lanost-8-en-21-oic acid	*G.* *resinaceum* (fruit bodies)	[62]
**298**	Colossolactone A	*G. colossum* (fruit bodies)	[98]
**299**	Methyl ganosinensate A	*G.* *sinense* (fruit bodies)	[56]
**300**	Ganosinensic acid A	*G. sinense* (fruit bodies)	[56]
**301**	Ganosinensic acid B	*G.* *sinense* (fruit bodies)	[56]
**302**	Tsugarioside C (3α-acetoxy-(*Z*)-24-methyl-5α-lanosta-8,23,25-trien-21-oic acid ester β-D-xyloside)	*G.* *tsugae* (fruit bodies)	[60]
**303**	Ganorbiformin A	*G.* *colossum* (fruit bodies)	[46]
**304**	Colossolactone V ((22*R*)-3,4-seco-19,22-diacetoxy-4-hydroxylanosta-8,24(*Z*)-dien-3,26-dioic acid 3-methyl-ester)	*G.* *colossum* (fruit bodies)	[102]
**305**	Colossolactone VI ((22*R*)-3,4-seco-19,22-diacetoxy-4-hydroxylanosta-7,9(11),24(*Z*)-trien-3,26-dioic acid 3-methyl ester)	*G.* *colossum* (fruit bodies)	[102]
**306**	Colossolactone VII ((22*S*)-3,4-seco-19-acetoxy-4-hydroxylanosta-8,24-dien-26,22-olide 3-methyl ester)	*G.* *colossum* (fruit bodies)	[102]
**307**	Furanoganoderic acid (21,23-epoxy-15α-hydroxy-3,7,1l-trioxo-5α-lanosta-8,20(21),22-trien-26-oic acid)	*G.* *applanatum* (fruit bodies)	[52]
**308**	Fornicatin B (7β-hydroxy-11-oxo-3,4-seco-25,26,27-trinorlanosta-4(28),8-dien-3,24-dioic acid)	*G.* *fornicatum* (fruit bodies)	[105]
**309**	Fornicatin G (7β-hydroxy-11-oxo-3,4-seco-25,26,27-trinorlanosta-4(28),8-dien-24-oic-3-acetyl ester)	*G.* *cochlear* (sporophore)	[106]
**310**	Fornicatin A (4, 7β-epoxy-28-hydroxy-11-oxo-3,4-seco-25,26,27-trinorlanosta-8-en-3,24-dioic acid)	*G. fornicatum* (fruit bodies)	[105]
**311**	Fornicatin H (4, 7β-epoxy-28-hydroxy-11-oxo-3,4-seco-25,26,27-trinorlansta-8-en-3,24-diester)	*G.* *cochlear* (sporophore)	[106]
**312**	Australic acid ((20*Z*, 23*R*, 25*R*)-15α-acetyl-7α, 8α-epoxy-12-oxo-3,4-seco-5α-lanosta-4(28),9,20(22)-trien-23,26-olid-3-oic acid)	*G.* *australe*	[103]
**313**	Lucidone A	*G.* *tsugae*	[107]
**314**	Lucidenol	*G. tsugae*	[107]
**315**	Ganosineniol A	*G.* *sinense* (fruit bodies)	[47]
**316**	8α, 9α-Epoxy-4,4,14α-trimethyl-3,7,11,15,20-pentaoxo-5α-pregnane	*G.* *concinna*	[75]

**Figure 32:**
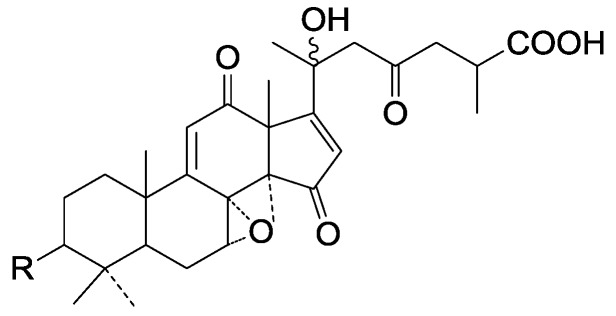
Structures of compounds **281**–**282**.

**Table:** 

Cpd	R
**281**	=O
**282**	β-OH

**Figure 33:**
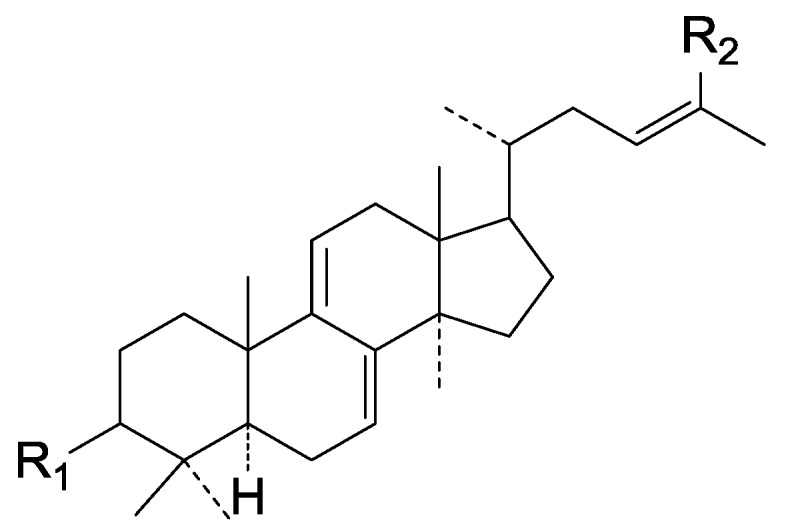
Structures of compounds **283**–**285**.

**Table:** 

Cpd	R	R2
**283**	α-OH	COOH
**284**	=O	CH_2_OH
**285**	β-OH	COOH

**Figure 34:**
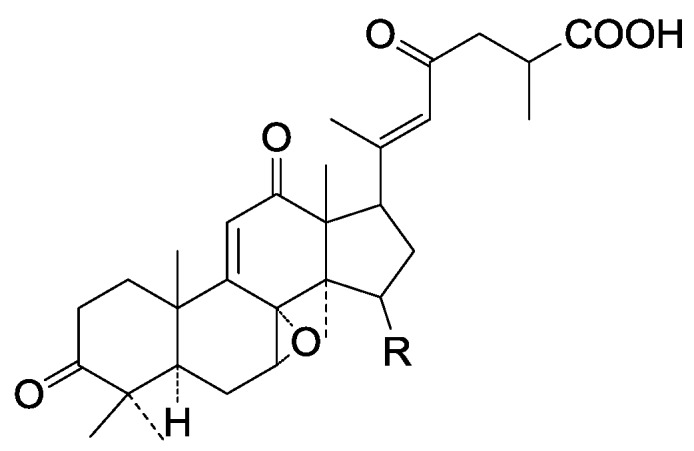
Structures of compounds **286**–**287**.

**Table:** 

Cpd	R
**286**	α-OH
**287**	=O

**Figure 35:**
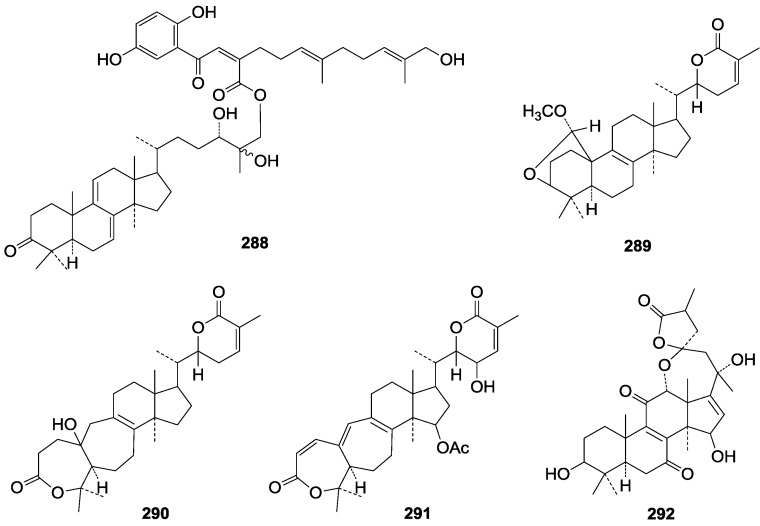

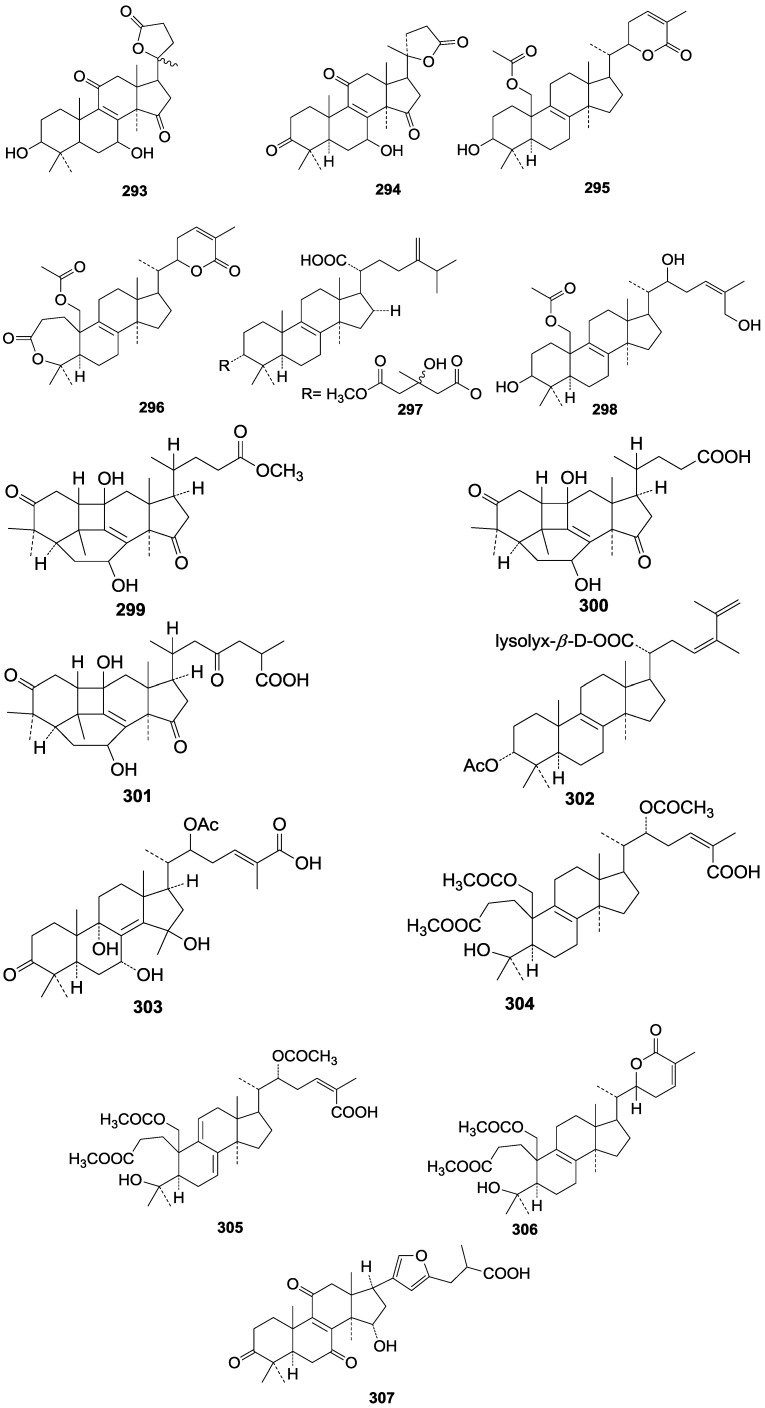
Structures of compounds **288**–**307**.

**Figure 36:**
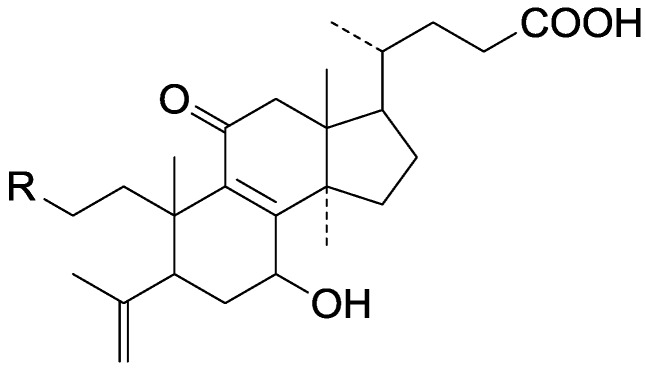
Structures of compounds **308**–**309**.

**Table:** 

Cpd	R
**308**	COOH
**309**	COOCH_2_CH_3_

**Figure 37:**
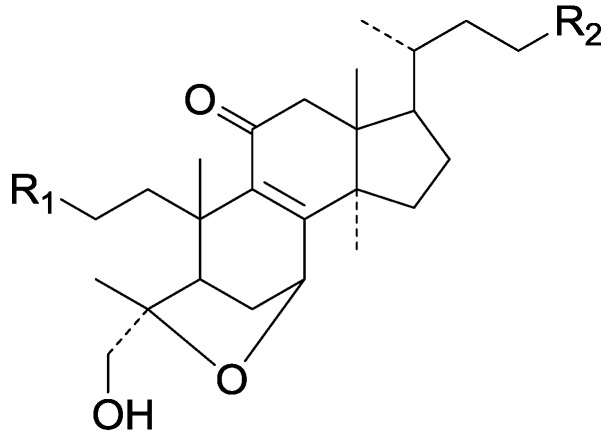
Structure of compounds **310**–**311**.

**Table:** 

Cpd	R1	R2
**310**	COOH	COOH
**311**	COOCH_3_	COOCH_3_

**Figure 38:**
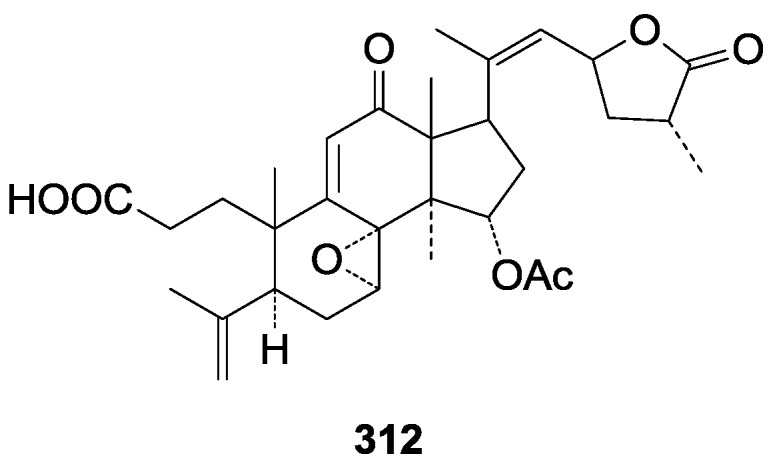
Structure of compound **312**.

**Figure 39:**
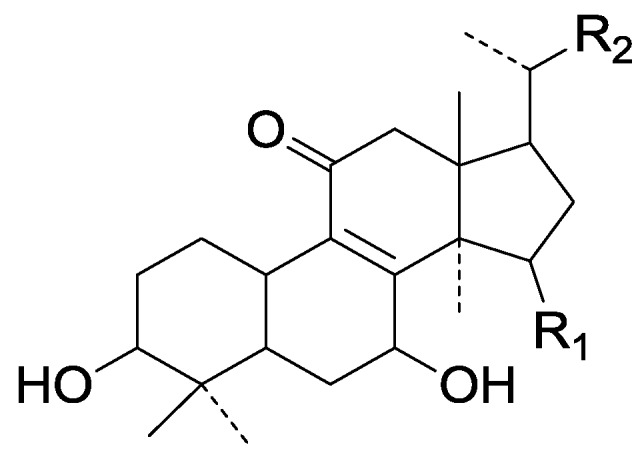
Structures of compounds **313**–**315**.

**Table:** 

Cpd	R1	R2
**313**	=O	=O
**314**	=O	OH
**315**	α-OH	CH_2_OH

**Figure 40:**
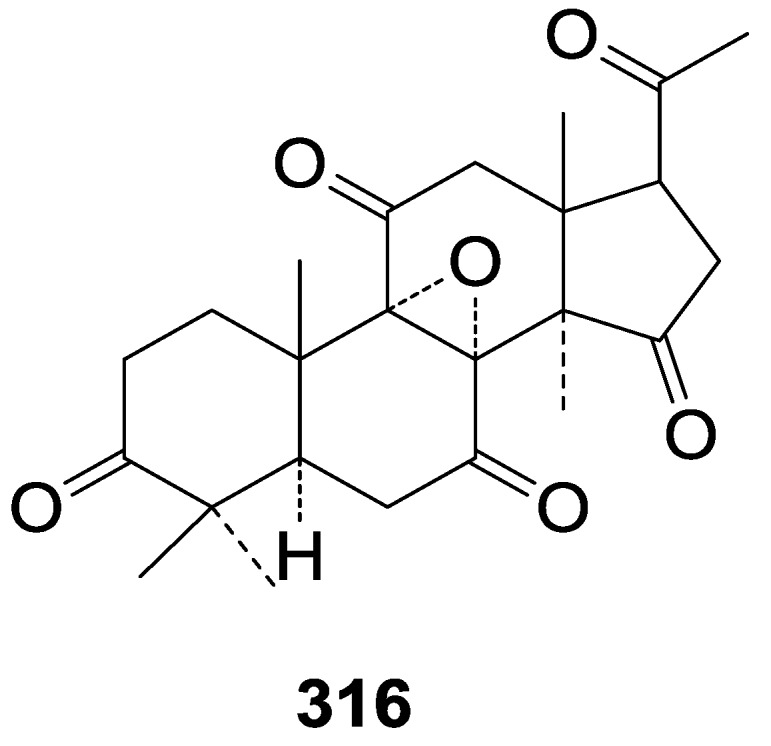
Structure of compound **316**.

## 3. ^13^C-NMR Data of *Ganoderma* Triterpenes

The reported GTs ^13^C-NMR data are shown in Table 12. For compounds **5**, **7**, **22**, **23**, **28**, **31**–**34**, **36**, **37**, **54**, **55**, **59**, **89**, **95**, **97**, **98**, **110**, **112**, **114**, **121**–**123**, **130**–**132**, **146**, **147**, **154**, **159**, **169**, **195**, **199**, **200**, **206**, **211**–**213**, **235**–**237**, **240**, **245**–**247**, **250**, **251**, **257**–**260**, **265**, **283**–**285**, **313** and **314** have no ^13^C-NMR data reported or cannot be researched.

As summarized above, a large number of triterpenes together with their potential pharmacological activities are described from *Ganoderma*. Being inclined to complement the prior reviews, we summarized the triterpenes from *Ganoderma*. They contain 30 or 27 carbon atoms, including some with 24 carbon atoms. The great majority of the triterpenes possess double bonds on the ring, at C-8 (9), with hydroxy and carbonyl substituted at C-3, C-7, C-11, and C-15 generally. For this type, the carbon atoms mentioned above are usually a characteristic for its structural determination. The ^13^C-NMR data of hydroxy substituted C-3 appear from 77–80 ppm, while the data of carbonyl substituted increase to 208–218 ppm. As to the double bonds, the resonance of C-8 arises at 131–165 ppm and the C-9 signal arises at 134–165 ppm, fluctuated for the neighbouring substituent groups.

In the other type, with double bonds located at C-7(8) and C-9(11), the resonance of C-7 appears from 119 ppm to 121 ppm, while the C-8 signal increases to 140–143 ppm. The C-9 signal appears at 144–147 ppm, while the C-11 signal moves to 115–118 ppm. C-23 tends to be oxidized to a carbonyl with ^13^C-NMR signals appearing at 206 ppm to 217 ppm, or to hydroxy with signals in the 65–67 ppm range. When double bonds appear between C-20 and C-22, the C-23 signal will move to 197–200 ppm. Moreover, C-24 and C-25 are sometimes linked by double bonds in some *Ganoderma* triterpenes. In this case, the ^13^C-NMR peaks of C-24 appear at 144–156 ppm and those of C-25 appear at 126–140 ppm. According to the compiled ^13^C-NMR data, this review should provide a useful and fast way for the identification of GTs.

**Table 12:** The ^13^C-NMR spectural data of compounds **1**–**316** except those which have no reported ^13^C-NMR data.

NO.	1^b)^	2^b)^	3^b)^	4^b)^	6^b)^	8^b)^	9^b)^	10^b)^	11^b)^	12^b)^	13^b)^	14^b)^	15^b)^	16^b)^	17^b)^	18^b)^	19^b)^	20^b)^
C1	33.4	35.7	35.0	33.1	35.1	34.4	35.7	35.6	34.8	34.8	35.0	34.7	35.7	35.7	34.6	34.3	34.8	34.2
C2	27.5	34.5	27.9	27.2	34.2	28.0	34.4	34.4	27.6	28.3	28.0	28.2	34.3	34.3	27.8	27.7	34.1	27.3
C3	77.5	217.1	78.5	77.3	217.7	78.7	208.4	208.7	78.3	78.5	77.5	78.2	217.5	217.5	78.2	77.5	217.8	78.5
C4	40.6	46.8	39.0	39.0	47.0	39.0	47.0	46.7	38.8	39.0	38.9	38.6	46.8	46.8	38.6	40.2	47.2	39.1
C5	51.5	49.0	49.3	51.2	51.7	51.8	49.2	48.8	49.2	45.7	49.5	49.1	49.0	49.0	49.1	49.8	45.2	47.7
C6	36.8	29.3	26.7	36.6	18.7	17.4	29.2	29.1	26.6	27.8	27.6	27.8	27.7	27.8	28.2	36.5	27.9	28.0
C7	199.1	69.1	67.1	199.0	29.6	30.4	69.1	68.9	66.9	67.0	69.5	69.5	66.4	66.3	69.5	205.3	66.7	68.0
C8	151.9	159.1	157.1	145.6	163.2	162.9	159.6	159.5	156.8	155.0	159.6	158.0	157.8	157.8	158.1	154.6	159.3	158.8
C9	145.9	140.5	142.9	151.7	138.6	140.0	140.6	140.1	142.7	142.9	142.2	142.0	141.3	141.3	141.9	149.8	140.0	141.6
C10	39.3	38.2	38.8	40.3	37.1	37.8	46.8	46.8	38.6	45.4	38.9	38.6	38.3	38.3	38.5	38.9	38.0	38.6
C11	194.2	199.6	198.0	193.9	198.1	198.3	200.0	199.7	197.9	200.0	201.2	199.8	197.6	197.6	199.9	201.3	199.1	199.4
C12	79.3	51.9	50.5	79.1	51.7	52.1	51.9	51.8	50.3	51.0	52.3	52.0	50.2	50.2	51.9	52.3	51.8	52.2
C13	48.1	47.0	45.5	47.9	46.8	47.2	38.2	38.0	45.4	38.8	47.4	47.1	45.0	45.0	47.1	48.0	46.4	46.1
C14	58.7	54.1	59.6	58.5	53.6	53.5	54.2	54.1	59.4	58.5	54.4	54.0	59.4	59.4	54.0	52.8	53.4	53.5
C15	205.9	72.7	217.7	66.2	72.9	73.0	72.6	72.4	217.5	207.5	72.4	72.4	216.6	216.4	72.5	72.1	72.4	72.3
C16	38.1	36.7	41.1	38.0	38.6	38.7	36.2	36.3	40.9	41.0	35.9	36.2	41.0	41.0	36.1	36.3	37.8	37.8
C17	44.9	48.4	45.8	44.6	48.7	48.7	48.3	48.2	45.6	49.2	48.5	48.1	45.7	45.8	48.1	48.2	49.0	49.0
C18	12.3	17.5	17.6	12.1	17.2	17.1	17.4	17.3	17.4	17.2	17.2	17.1	17.7	17.7	17.1	17.4	17.5	17.3
C19	18.1	19.5	18.7	17.9	19.0	19.0	19.6	19.4	18.5	17.6	19.6	19.5	18.2	18.2	19.6	17.6	17.5	17.3
C20	29.6	32.8	32.1	29.4	32.6	32.5	32.8	32.8	32.0	32.1	33.0	32.7	32.0	32.0	32.7	32.4	32.5	32.5
C21	21.8	19.7	19.8	21.5	19.4	19.4	19.4	19.7	19.6	19.8	19.7	19.6	19.6	19.7	19.6	19.5	19.3	19.3
C22	48.6	49.9	49.4	48.5	49.6	49.7	49.8	49.7	49.0	49.3	50.0	49.8	49.0	49.1	49.7	49.5	49.6	49.6
C23	207.7	208.6	207.9	206.1	208.3	208.3	217.3	217.4	207.6	215.9	210.0	208.5	207.5	207.6	208.7	208.2	208.3	208.3
C24	46.8	46.9	46.8	46.6	46.8	46.8	46.7	46.8	46.6	46.9	46.9	46.7	46.6	46.8	46.7	46.8	46.9	46.9
C25	34.9	35.0	35.1	35.1	34.7	34.6	34.8	34.8	34.6	34.9	35.0	34.7	34.5	34.7	34.6	34.7	34.7	34.7
C26	175.9	176.0	175.9	181.0	176.2	176.1	27.4	27.5	180.3	26.8	178.5	176.2	180.3	176.1	176.3	176.1	176.2	176.4
C27	17.3	17.3	17.3	17.1	17.1	17.1	180.1	176.3	16.9	176.2	17.2	17.1	16.9	17.1	17.1	17.1	17.1	17.1
C28	28.1	27.5	28.4	27.8	--	--	--	--	--	--	--	--	--	--	--	--	--	--
C29	15.7	20.9	15.6	15.5	--	--	--	--	--	--	--	--	--	--	--	--	--	--
C30	21.5	19.8	24.6	21.2	27.8	28.3	17.0	17.2	28.2	15.5	28.3	28.2	27.0	27.0	28.2	27.8	27.6	28.2
	OCOCH_3_	Bu1'	Bu1'	CH_3_CO	C31	C31	C31	C31	C31	C31	C31	C31	C31	C31	C31	C31	C31	C31
	170.4	64.8	64.8	170.2	20.6	15.7	20.8	20.8	15.5	24.5	15.9	15.7	20.8	20.8	15.7	15.4	20.5	15.8
	OCOCH_3_	Bu 2'	Bu 2'	CH_3_CO	C32	C32	C32	C32	C32	C32	C32	C32	C32	C32	C32	C32	C32	C32
	21.0	30.9	30.9	20.9	18.8	18.8	19.8	19.7	24.4	18.7	19.5	19.4	24.7	24.7	19.4	20.3	21.1	21.1
	N-BU1'	Bu 3'	Bu3'		OCH_3_	OCH_3_		COOCH_3_		COOCH_3_		COOCH_3_		COOCH_3_	OCH_3_	OCH_3_	OCH_3_	OCH_3_
	64.8	19.3	19.3		51.9	51.8		52.0		51.7		51.9		51.9	51.9	51.9	51.9	51.9
**NO.**	**21^b)^**	**24^b)^**	**25^b)^**	**26^b)^**	**27^c)^**	**29^b)^**	**30^b)^**	**35^c)^**	**38^b)^**	**39^b)^**	**40^b)^**	**41^b)^**	**42^d)^**	**43^b)^**	**44^a)^**	**45^b)^**	**46^b)^**	**47^c)^**
C1	34.5	33.9	34.4	34.5	35.4	37.4	37.2	35.2	35.6	35.7	34.4	33.8	34.0	34.8	36.0	35.4	34.8	35.2
C2	24.0	33.6	27.4	28.4	34.7	34.1	34.6	27.6	34.4	34.5	27.4	27.5	33.5	27.4	34.5	34.3	28.8	33.9
C3	79.9	215.1	78.2	78.4	221.0	214.9	215.4	78.0	216.8	218.0	78.2	78.5	215.5	78.0	215.9	214.6	78.0	218.6
C4	38.0	46.8	38.6	40.7	47.2	46.9	47.0	39.1	46.2	47.0	38.5	38.8	38.7	39.0	47.0	47.2	39.8	46.4
C5	49.2	50.8	49.1	53.0	45.7	51.0	50.9	49.8	48.8	49.0	49.1	51.3	49.7	49.9	48.7	50.4	50.7	48.3
C6	26.6	37.4	36.7	37.8	28.1	33.7	33.8	27.4	27.6	29.2	26.6	17.3	36.8	36.6	28.8	37.1	36.7	28.1
C7	66.1	198.6	66.2	201.1	67.1	198.6	199.4	67.0	66.3	69.2	66.2	29.3	199.6	199.1	66.0	198.0	199.0	68.2
C8	157.4	149.8	155.8	151.9	161.8	149.9	149.8	157.3	157.8	159.2	155.9	161.5	146.1	138.9	159.7	139.8	138.9	160.4
C9	141.6	145.9	142.9	153.6	140.2	146.1	146.8	143.1	141.2	140.6	142.0	140.1	149.0	164.9	140.9	162.6	164.7	139.6
C10	37.5	39.2	38.5	42.4	38.4	39.4	39.3	38.9	38.2	38.2	38.5	37.5	46.4	39.8	38.4	39.4	38.9	37.6
C11	199.3	194.1	192.0	195.0	72.0	194.1	199.4	200.3	197.7	200.1	192.1	191.6	199.8	23.7	198.2	23.8	23.6	200.3
C12	77.9	78.9	79.5	199.8	52.4	79.0	48.9	78.4	50.1	52.0	79.8	80.1	48.5	30.2	50.9	30.1	30.2	51.5
C13	51.9	47.6	49.6	62.2	47.6	47.7	43.9	52.4	44.9	46.9	49.9	51.5	56.8	45.0	45.2	45.0	45.0	46.3
C14	60.3	58.7	60.6	61.3	54.1	58.7	57.2	60.9	59.3	54.1	60.5	53.9	43.5	47.8	59.0	47.8	49.0	53.7
C15	216.8	205.6	216.2	207.5	201.0	205.5	207.0	217.8	218.1	72.9	216.4	74.6	207.8	32.0	217.0	28.7	27.5	71.6
C16	38.3	37.8	37.9	40.8	35.3	37.8	39.8	38.6	41.2	36.7	37.3	33.6	39.9	28.8	42.0	31.8	32.0	35.6
C17	45.7	44.3	45.2	40.7	49.5	44.5	44.3	46.6	46.7	48.7	46.1	48.6	44.4	49.0	46.8	49.0	49.9	48.5
C18	12.0	12.0	12.0	13.5	17.8	12.1	16.0	12.3	17.7	17.4	13.4	12.3	15.4	15.8	17.8	15.9	15.3	16.6
C19	18.8	18.7	18.6	18.6	17.9	18.7	18.6	19.1	18.2	19.6	18.6	19.0	18.4	18.4	18.2	17.9	18.4	18.9
C20	28.7	29.4	28.2	33.8	33.2	29.5	32.1	29.1	35.5	36.2	35.5	34.1	32.9	36.2	33.9	36.2	36.3	33.1
C21	21.3	21.6	21.9	20.4	19.5	21.6	19.8	21.8	18.2	18.5	20.8	19.7	19.7	18.6	19.8	18.6	18.6	19.0
C22	48.3	48.4	47.9	50.2	50.0	48.4	48.8	48.7	34.5	34.8	33.1	33.7	42.8	34.8	43.8	34.7	34.8	42.9
C23	208.1	207.6	207.4	211.1	210.7	207.3	207.6	210.3	25.6	25.9	26.5	26.3	65.4	25.9	66.5	26.0	26.0	65.9
C24	46.1	46.6	46.6	48.0	47.2	46.4	46.5	46.9	144.1	145.3	143.2	144.2	144.4	145.6	145.0	155.2	155.3	143.2
C25	34.2	34.7	34.6	36.6	37.4	34.6	34.6	35.4	127.0	127.2	127.1	127.2	126.9	126.6	128.8	139.2	139.2	128.4
C26	180.5	175.6	176.1	180.7	179.0	180.8	180.9	178.8	171.2	172.0	171.0	172.7	169.0	172.4	170.7	195.3	195.3	170.2
C27	16.9	17.1	17.1	18.0	17.4	16.9	16.9	17.3	12.1	12.3	12.1	12.0	12.7	12.0	13.4	9.2	9.2	12.3
C28	27.9	27.6	28.0	28.7	27.8	--	--	--	24.7	19.7	24.1	28.2	27.0	25.0	27.0	25.4	27.5	27.0
C29	16.4	20.3	15.4	16.6	20.7	--	--	--	26.9	27.6	28.1	15.7	19.9	27.5	20.8	21.4	15.8	20.1
C30	23.0	20.7	24.0	24.9	21.4	27.6	27.6	28.4	20.7	20.9	15.4	19.8	20.0	15.3	25.1	24.9	25.0	19.0
	C31	CH_3_CO	CH_3_CO			C31	C31	C31			CH_3_CO	12-COCH_3_						
	161.0	170.2	170.4			20.4	20.3	15.8			170.1	170.5						
		CH_3_CO	CH_3_CO			C32	C32	C32			CH_3_CO	12-COCH_3_						
		20.9	20.9			20.8	21.0	23.5			20.7	21.0						
		OCH_2_CH_3_	OCH_3_			OCOCH_3_						15-COCH_3_						
		60.7	51.9			170.2						170.6						
**NO**	**48^c)^**	**49^c)^**	**50^c)^**	**51^c)^**	**52^c)^**	**53^b)^**	**56^b)^**	**57^b)^**	**58^b)^**	**60^b)^**	**61^b)^**	**62^b)^**	**63^b)^**	**64^b)^**	**65^b)^**	**66^a)^**	**67^a)^**	**68^a)^**
C1	34.9	35.7	33.6	34.4	33.2	34.7	30.1	35.0	35.3	34.8	34.5	34.7	34.6	35.4	35.2	35.5	35.2	35.2
C2	34.3	28.0	26.9	27.1	26.7	27.6	23.3	34.1	34.3	34.6	34.4	27.3	27.3	34.4	34.3	29.0	28.9	34.1
C3	219.5	78.7	77.2	78.0	76.8	78.3	77.3	215.5	214.6	214.4	214.9	77.8	77.8	214.6	217.2	77.6	77.9	218.4
C4	46.6	39.7	39.0	38.4	40.0	38.8	36.2	46.9	47.2	47.5	47.4	38.8	38.8	47.2	46.6	39.3	39.7	45.9
C5	45.3	50.0	50.8	49.0	51.2	49.1	40.5	49.8	50.4	50.8	51.6	49.7	49.8	49.0	44.8	49.9	52.4	48.6
C6	27.7	27.6	36.2	26.5	36.4	26.6	26.1	37.1	37.1	37.5	37.2	36.5	36.5	37.2	28.4	28.8	30.9	28.8
C7	66.6	67.7	199.9	66.2	199.5	66.8	70.0	201.3	198.1	199.4	199.8	199.0	198.9	198.0	66.2	69.4	17.9	68.6
C8	160.9	158.4	148.6	156.6	146.2	156.9	131.1	151.5	139.6	142.1	139.7	138.9	138.8	139.6	134.8	160.5	164.8	159.8
C9	139.7	143.9	151.8	142.0	151.0	142.7	145.1	149.8	162.7	158.7	160.6	164.8	164.6	162.8	140.1	141.3	139.8	140.3
C10	37.9	39.4	40.4	38.1	38..8	38.6	38.3	38.8	39.4	40.1	39.7	39.7	39.7	39.4	38.1	39.1	38.3	37.5
C11	200.1	200.1	200.1	199.5	201.4	198.0	21.4	201.8	23.8	65.8	67.2	23.6	23.5	23.8	20.8	200.1	198.6	199.8
C12	52.0	51.2	49.6	78.2	77.6	50.3	31.2	51.2	30.1	44.6	42.7	30.0	30.1	30.2	31.2	52.9	52.9	51.6
C13	47.0	46.4	44.2	51.7	49.5	45.3	45.8	46.7	44.9	47.6	43.4	44.8	44.9	45.0	45.3	47.6	47.4	46.2
C14	53.4	60.3	57.0	60.1	57.5	59.3	52.1	48.7	47.8	48.1	48.9	47.7	47.7	47.8	51.2	54.7	54.0	53.7
C15	71.7	218.5	209.0	217.4	207.6	217.9	72.4	31.9	31.9	32.6	31.9	31.9	31.9	28.7	76.4	72.4	72.1	72.1
C16	37.5	42.0	45.6	36.8	36.7	41.1	39.7	27.43	28.7	27.9	28.7	28.7	28.7	31.9	36.4	37.5	39.6	36.2
C17	49.7	47.2	42.7	46.2	45.5	46.1	48.8	49.1	49.0	49.7	48.1	48.9	48.9	50.5	49.3	49.5	49.9	47.5
C18	17.3	17.6	15.9	11.9	10.6	17.4	16.3	16.9	15.9	16.9	17.3	15.7	15.7	15.9	16.6	17.4	17.0	17.0
C19	17.5	18.8	17.6	18.6	17.6	18.4	17.7	17.9	17.9	19.2	19.5	18.2	18.2	17.9	17.3	19.9	19.4	19.3
C20	33.5	34.0	33.1	28.5	29.4	35.5	36.2	36.1	36.2	36.0	36.2	36.1	36.1	36.2	36.2	34.6	34.4	35.9
C21	19.2	20.0	19.3	22.0	21.3	18.2	18.4	18.3	18.7	18.4	18.6	18.6	18.5	18.4	18.2	20.2	19.9	18.4
C22	43.3	43.7	40.4	41.3	41.8	37.4	34.7	34.5	35.9	34.6	34.6	35.8	34.7	34.7	34.6	44.5	44.4	34.4
C23	66.4	66.8	65.9	67.0	66.5	25.6	25.8	25.92	24.5	25.8	25.8	24.4	25.9	25.9	25.9	67.0	66.9	26.0
C24	142.4	144.2	142.8	142.7	142.2	143.9	145.2	154.6	126.8	145.3	145.3	126.8	155.1	145.5	144.9	145.4	145.4	156.3
C25	130.2	129.6	128.8	129.3	129.0	127.5	126.8	139.4	134.4	126.8	126.7	134.3	139.1	126.5	126.7	128.7	128.7	138.6
C26	172.0	171.2	170.8	170.8	175.0	172.3	172.4	195.2	69.0	172.7	172.7	69.0	195.3	172.5	171.2	170.8	170.8	194.4
C27	13.1	13.1	12.6	12.8	12.6	12.1	12.0	9.2	13.6	11.9	11.9	13.5	9.0	12.0	12.1	13.5	13.5	9.2
C28	27.5	28.6	27.5	27.9	27.4	28.1	27.2	27.44	25.4	25.1	25.1	24.9	24.9	25.0	26.5	28.8	28.9	20.8
C29	20.5	16.1	15.3	15.2	15.2	15.4	22.7	20.3	21.4	21.6	21.8	27.3	27.3	25.9	21.2	16.7	16.7	27.5
C30	21.0	24.9	21.6	22.9	20.1	24.4	17.9	25.87	24.9	25.3	24.9	15.2	15.2	21.4	20.1	20.2	19.8	19.1
							COCH_3_											
							171.9											
							COCH_3_											
							21.9											
**NO.**	**69^c)^**	**70^b)^**	**71^b)^**	**72^b)^**	**73^b)^**	**74^b)^**	**75^b)^**	**77^b)^**	**78^b)^**	**79^b)^**	**80^b)^**	**81^b)^**	**82^b)^**	**83^h)^**	**84^b)^**	**85^b)^**	**86^b)^**	**87^b)^**
C1	34.9	30.3	30.3	30.1	30.2	30.3	30.3	30.3	34.5	34.8	35.2	35.3	35.3	31.3	30.0	33.3	35.9	35.6
C2	34.0	23.3	23.3	23.3	23.4	23.2	23.3	23.3	23.8	27.4	34.2	34.3	34.3	24.3	23.3	27.3	34.5	34.2
C3	219.6	77.5	77.5	77.3	77.6	77.3	77.5	77.6	79.6	77.9	217.2	217.4	217.4	77.7	77.2	77.4	216.7	216.4
C4	46.8	36.3	36.3	36.2	36.4	36.5	36.4	36.3	37.8	38.9	46.6	46.7	46.7	37.2	36.3	39.1	47.0	46.7
C5	51.3	40.1	40.0	40.5	40.4	40.1	40.5	40.2	49.9	49.8	44.7	45.0	45.0	40.8	39.6	51.2	49.2	48.8
C6	18.4	27.4	27.4	26.0	22.4	21.3	28.1	28.9	36.4	36.6	28.4	30.0	23.3	28.5	21.3	36.5	27.9	27.6
C7	29.3	66.5	66.5	69.9	76.5	76.3	66.8	67.0	198.6	198.9	66.1	66.7	76.1	67.1	75.9	198.5	66.5	66.2
C8	165.1	133.9	133.8	131.1	134.3	132.8	134.5	135.6	138.9	138.8	134.6	136.4	135.3	141.7	142.9	145.8	157.8	157.3
C9	137.9	141.8	141.8	145.0	141.3	143.6	141.7	141.3	164.4	164.6	140.1	139.4	139.5	135.1	132.7	151.9	141.4	141.2
C10	36.8	38.4	38.4	38.3	38.1	38.4	38.1	38.2	39.6	39.8	38.1	37.9	37.8	39.2	38.7	40.5	38.6	38.3
C11	199.1	20.7	20.7	21.2	21.0	20.9	20.6	21.0	23.6	23.6	20.7	21.3	21.0	21.5	21.1	193.5	197.5	196.8
C12	51.6	31.2	31.2	31.2	31.1	31.4	31.7	31.0	30.1	30.1	31.2	31.0	31.1	32.1	30.7	78.5	49.2	48.9
C13	46.4	45.5	45.3	45.6	45.0	46.2	45.7	45.0	44.9	44.9	45.1	45.0	44.9	46.0	45.6	57.8	45.9	45.9
C14	53.3	51.2	51.3	52.1	50.0	52.8	52.4	49.7	47.8	47.8	51.2	49.7	49.9	52.4	51.7	48.7	58.8	58.6
C15	72.0	76.5	76.0	72.2	30.2	72.0	72.4	29.8	31.9	31.9	75.9	29.9	30.1	76.0	75.2	204.6	217.3	216.6
C16	38.1	36.6	36.3	39.3	27.9	37.3	38.1	27.9	28.5	28.5	36.1	27.9	27.8	37.3	37.1	37.6	38.1	37.8
C17	49.0	49.3	45.9	45.5	47.2	46.4	46.4	47.1	45.6	45.7	45.9	47.1	47.2	46.9	45.2	48.9	48.3	49.7
C18	16.7	16.5	16.3	16.2	15.9	16.6	16.4	15.9	15.6	15.6	16.4	16.0	16.0	17.0	16.1	13.3	19.2	19.0
C19	18.6	17.5	17.5	17.7	17.5	17.2	17.3	17.3	18.5	18.4	17.3	17.3	17.4	18.0	17.6	17.8	18.4	18.1
C20	33.1	36.3	39.9	39.5	39.8	39.3	39.5	39.7	39.5	39.5	39.9	39.7	39.7	41.0	39.9	154.7	138.5	153.3
C21	18.8	18.2	12.7	12.9	12.9	13.0	12.9	12.8	13.1	13.1	12.6	12.8	12.8	13.6	12.7	21.1	18.3	21.0
C22	42.9	34.7	74.4	74.6	74.8	74.9	74.7	74.7	74.8	74.8	74.3	74.7	74.7	74.8	74.3	126.0	126.9	124.7
C23	66.0	25.9	31.9	31.7	31.9	31.9	31.9	31.8	31.8	31.8	31.9	31.8	31.8	32.8	31.8	197.8	74.5	197.9
C24	142.4	145.0	138.9	139.1	139.6	139.1	139.1	139.4	139.4	139.4	138.8	139.6	139.5	140.0	139.1	47.5	37.2	47.7
C25	129.5	126.8	129.5	129.3	129.1	129.3	129.3	129.2	129.0	129.0	129.5	129.2	129.0	130.2	129.3	34.4	34.5	34.8
C26	171.5	172.2	172.0	171.7	172.1	172.0	171.4	172.4	171.3	171.2	171.9	172.0	171.7	172.9	172.2	180.2	179.8	176.3
C27	12.7	12.0	12.3	12.3	12.3	12.3	12.4	12.3	12.3	12.3	12.3	12.3	12.3	13.1	12.3	17.0	16.0	17.2
C28	27.5	27.4	27.4	27.2	27.3	27.3	27.4	27.4	27.4	27.4	26.5	26.5	26.5	--	--	27.9	27.3	27.0
C29	20.2	21.9	22.0	21.9	22.2	22.0	21.9	22.0	16.3	15.3	21.2	21.3	21.3	--	--	15.5	21.0	20.8
C30	18.7	20.2	20.2	18.0	25.6	18.5	19.2	26.3	25.1	25.0	26.1	26.1	25.4	28.2	27.3	21.3	24.8	24.7
		OCOCH_3_	COCH_3_	COCH_3_	COCH_3_	COCH_3_	COCH_3_	CO	3-OC-	22-OC-	15-OC-	22-OC-	7-OCH_3_	C31	C31	C31		OCH_3_
		170.9	170.9	171.9	171.1	170.9	170.9	170.9	OCH_3_	OCH_3_	OCH_3_	OCH_3_	55.8	22.6	22.2	170.3		51.9
		OCOCH_3_	COCH_3_	COCH_3_	COCH_3_	COCH_3_	COCH_3_	CO	170.8	170.6	170.5	170.7		C32	C32	C32		
		21.4	21.4	21.7	21.6	21.5	21.4	170.7						21.1	19.0	20.5		
**NO.**	**88^c)^**	**89^c)^**	**90^b)^**	**91^b)^**	**92^b)^**	**93^h)^**	**94^h)^**	**96^h)^**	**99^b)^**	**100^b)^**	**101^b)^**	**102^b)^**	**103^b)^**	**104^b)^**	**105^b)^**	**106^b)^**	**107^b)^**	**108^b)^**
C1	35.5	34.8	34.8	35.6	35.1	34.9	35.2	34.5	35.7	37.3	37.5	36.6	34.6	35.2	33.4	35.0	35.1	35.9
C2	34.3	27.7	27.8	34.2	34.1	34.7	33.9	27.9	34.3	34.7	34.1	27.3	27.6	34.3	27.4	33.9	28.0	34.5
C3	217.4	78.3	78.1	216.5	215.9	213.3	213.0	77.0	217.5	217.2	214.8	77.3	78.3	216.8	77.5	215.4	78.6	216.8
C4	46.7	39.0	38.8	46.8	46.8	46.8	46.3	40.4	46.8	43.9	46.9	40.4	38.6	47.0	40.3	46.4	39.1	47.0
C5	48.8	49.2	50.0	48.9	49.2	48.5	49.0	50.1	49.0	50.9	51.0	51.4	49.2	49.5	51.5	49.0	49.4	49.2
C6	29.0	26.7	27.6	27.7	27.6	36.1	36.8	36.5	27.8	33.8	33.7	33.2	26.9	27.8	36.8	36.8	26.9	27.9
C7	68.9	66.9	69.4	66.3	65.8	198.4	204.5	205.0	66.3	199.3	198.7	198.7	66.2	65.8	198.8	204.6	67.1	66.5
C8	159.1	156.6	158.8	157.4	156.7	146.9	150.3	149.6	157.8	149.7	149.9	151.6	157.4	158.3	150.5	150.4	156.9	157.9
C9	140.3	142.8	142.0	141.4	141.6	149.5	152.6	154.6	141.3	146.8	146.1	145.7	141.9	140.5	147.1	152.3	142.7	141.3
C10	38.1	38.7	38.8	38.4	38.2	39.5	39.2	38.8	38.3	39.4	39.3	39.1	38.4	37.9	39.2	39.1	38.9	38.5
C11	199.1	198.0	200.0	197.8	191.8	198.1	199.8	200.1	197.6	199.3	194.1	193.9	199.3	199.5	201.6	201.2	198.0	197.8
C12	50.5	49.1	50.8	48.9	78.5	47.8	50.5	51.0	50.2	48.9	79.0	79.1	77.9	78.1	77.5	52.1	51.1	50.9
C13	48.1	46.3	49.3	46.0	50.3	44.8	48.6	49.2	45.0	47.0	47.7	47.9	51.9	51.7	49.8	47.8	46.0	45.7
C14	53.4	58.7	53.5	58.7	59.9	56.8	52.4	52.5	59.4	57.2	58.6	58.4	60.3	60.4	57.9	52.8	59.8	59.8
C15	72.7	216.4	72.5	216.4	215.1	204.9	72.9	72.9	216.4	206.8	205.4	205.5	216.8	215.5	206.0	71.8	217.9	218.0
C16	31.8	37.7	31.6	37.9	38.4	37.1	32.3	32.5	41.0	39.8	37.8	37.8	38.4	38.5	37.9	30.2	36.2	36.4
C17	52.2	49.7	52.4	49.7	50.1	50.8	52.0	52.1	45.8	44.5	44.5	44.7	45.8	45.8	45.3	50.9	50.2	50.4
C18	19.0	18.8	18.8	19.0	14.4	17.5	16.7	15.5	17.7	16.1	12.1	12.1	12.0	12.1	10.9	18.9	19.1	19.4
C19	19.9	18.4	19.7	18.1	18.1	18.4	17.4	17.3	18.2	18.6	18.7	17.9	18.8	18.3	18.1	17.5	18.6	18.4
C20	157.0	153.8	157.3	153.6	154.1	153.6	155.6	155.7	32.0	32.0	29.4	29.3	28.7	28.7	29.5	73.3	73.4	73.3
C21	21.3	21.0	21.3	20.9	20.3	21.5	21.1	21.1	19.7	19.8	21.6	21.6	21.4	21.4	21.3	26.6	26.4	26.3
C22	124.3	124.7	124.5	124.7	126.1	124.6	124.5	124.6	49.1	49.1	48.5	48.4	48.4	48.5	48.9	52.5	48.5	48.5
C23	198.6	197.2	199.6	196.9	197.8	197.2	197.4	197.3	207.6	207.6	207.4	207.4	208.1	208.1	208.1	211.2	74.8	74.8
C24	47.5	47.6	48.1	47.5	47.5	47.6	47.6	47.9	46.8	46.7	46.7	46.6	46.4	46.4	46.6	47.7	36.8	36.8
C25	35.1	34.8	34.8	34.8	34.4	35.1	35.2	35.2	34.7	34.6	34.7	34.6	34.6	34.7	34.7	34.5	33.8	33.7
C26	180.2	180.0	179.0	180.6	180.0	181.2	181.2	175.9	176.1	176.1	176.0	176.0	176.1	176.1	176.1	177.8	178.8	178.8
C27	17.1	17.1	17.3	17.0	17.0	17.0	17.0	17.3	17.1	17.1	17.1	17.0	17.1	17.1	17.1	17.0	16.1	16.1
C28	--	--	--	--	26.6	27.2	27.0	27.7	--	--	--	--	--	--	--	27.2	28.4	27.2
C29	--	--	--	--	21.0	20.3	20.3	18.7	--	--	--	--	--	--	--	20.2	15.7	21.0
C30	27.5	28.2	28.2	27.0	24.0	20.9	20.7	20.7	27.0	27.6	27.6	27.9	28.1	26.3	28.0	20.6	25.1	25.3
	C31	C31	C31	C31	CH_3_CO			CO-	C31	C31	C31	C31	C31	C31	C31			
	20.7	15.5	15.8	20.8	170.6			OCH_3_	20.8	20.9	20.8	15.5	15.4	21.3	15.6			
	C32	C32	C32	C32	CH_3_CO			51.4	C32	C32	C32	C32	C32	C32	C32			
	19.4	24.4	19.5	24.7	20.6				24.7	20.3	20.4	21.2	23.1	23.3	20.3			
**NO.**	**109^f)^**	**111^b)^**	**113^b)^**	**115^a)^**	**116^b)^**	**117^a)^**	**118^b)^**	**119^b)^**	**120^b)^**	**124^b)^**	**125^b)^**	**126^b)^**	**127^b)^**	**128^h)^**	**129^b)^**	**133^a)^**	**134^b)^**	**135^b)^**
C1	35.9	35.3	35.4	35.2	34.9	35.9	30.2	30.4	36.1	34.6	34.6	34.2	34.9	34.5	35.7	35.2	35.5	35.4
C2	28.4	34.4	34.4	28.9	27.5	28.5	23.3	23.4	34.7	27.5	27.7	27.3	27.8	33.9	34.3	34.3	34.4	34.3
C3	79.2	214.8	214.6	77.1	78.0	77.8	78.1	78.0	216.3	78.2	78.2	78.2	78.4	208.9	217.8	214.9	217.3	214.6
C4	39.9	47.2	47.3	40.1	38.8	39.3	36.7	36.8	47.3	38.6	38.7	38.5	38.9	46.2	45.3	47.1	46.7	47.2
C5	50.5	50.3	49.1	49.6	49.9	50.7	45.3	45.3	51.3	49.3	49.3	49.2	49.2	49.1	49.4	50.2	50.7	50.4
C6	28.4	37.1	37.2	37.2	36.5	18.5	18.0	18.0	19.6	26.0	26.1	26.0	26.7	36.8	27.7	37.0	28.2	37.1
C7	67.7	198.2	198.1	198.7	198.8	26.6	26.0	26.0	26.5	67.1	67.1	66.6	66.9	203.5	66.3	198.2	66.8	198.0
C8	157.3	139.5	139.6	138.9	138.8	134.1	134.0	133.8	135.0	158.0	158.1	158.3	156.6	151.1	157.6	139.4	136.7	139.5
C9	144.2	162.8	162.8	164.9	164.6	134.1	134.6	134.6	133.9	142.2	142.1	142.6	142.3	152.6	141.0	163.0	139.6	162.6
C10	39.8	39.4	39.5	39.5	39.7	37.2	36.9	36.9	37.1	39.1	39.2	38.7	38.7	38.9	38.3	39.3	38.0	39.4
C11	199.4	23.8	23.9	23.8	23.7	21.0	21.0	20.8	21.4	197.4	197.3	192.2	197.8	200.9	197.6	23.8	21.1	23.8
C12	78.7	30.1	30.2	30.5	30.2	30.6	30.8	30.8	29.0	44.3	44.4	75.8	50.7	78.5	50.5	30.0	31.0	30.1
C13	53.3	44.9	45.0	45.2	45.0	44.7	44.3	44.3	45.0	51.4	51.4	54.0	45.7	54.4	46.8	44.8	45.1	44.9
C14	62.7	47.4	47.8	48.2	47.8	49.6	49.9	49.6	49.9	58.3	58.3	59.4	59.7	55.0	59.7	47.7	49.7	47.8
C15	217.4	28.6	28.7	28.3	32.0	27.3	27.5	27.0	31.0	210.9	210.8	209.9	217.7	72.1	216.8	31.8	30.0	31.9
C16	38.6	31.8	31.9	32.7	28.7	29.2	29.7	29.0	27.3	122.4	122.3	122.5	36.1	33.3	36.3	28.6	29.9	28.7
C17	53.9	49.0	50.5	50.6	49.0	47.5	40.6	47.2	47.6	187.6	187.6	187.1	49.3	55.3	49.0	48.8	45.1	49.0
C18	13.8	15.9	16.0	16.1	15.9	16.1	16.1	16.0	16.5	30.9	30.9	26.3	19.0	13.1	19.3	15.8	16.2	15.9
C19	19.5	24.9	17.9	18.3	18.4	19.2	19.0	18.9	18.6	18.5	18.5	18.4	18.4	17.3	18.1	17.8	17.2	17.9
C20	73.8	36.6	36.6	37.3	36.7	48.9	44.9	47.5	48.5	28.6	28.6	29.1	73.0	72.9	72.9	36.0	36.4	36.2
C21	28.3	18.9	18.9	19.3	19.0	178.4	70.2	182.0	175.8	19.4	19.4	18.5	26.7	26.4	26.7	18.5	18.5	18.7
C22	52.3	33.4	33.6	34.5	33.5	33.0	30.7	32.5	33.4	47.6	47.6	47.4	52.7	51.2	52.7	36.0	32.8	32.6
C23	211.5	28.6	28.8	29.1	28.6	26.1	24.7	25.9	26.4	205.8	205.9	206.1	210.4	208.9	210.4	24.2	25.3	25.0
C24	49.4	79.5	79.1	77.1	79.6	124.0	124.8	123.6	124.7	46.1	46.3	46.0	47.7	48.3	47.7	131.4	60.6	60.3
C25	36.2	73.2	73.9	74.8	73.2	136.7	131.4	132.2	131.9	34.4	34.6	34.2	34.5	35.0	34.5	136.7	60.7	60.7
C26	180.2	23.2	67.7	69.3	23.3	67.8	17.7	17.6	25.8	179.9	176.0	180.3	175.9	176.3	175.9	67.4	65.4	65.5
C27	17.8	26.5	20.9	20.1	26.6	13.7	25.7	25.7	17.8	16.9	17.1	16.7	17.0	17.2	17.0	59.8	14.2	14.2
C28	28.9	25.3	25.4	27.9	25.0	--	27.6	27.6	26.4	28.2	28.2	28.0	--	27.9	27.0	25.2	26.5	25.4
C29	16.4	21.4	21.4	16.0	27.5	--	21.8	21.8	21.3	15.5	15.5	15.3	--	19.6	20.8	21.3	21.3	21.4
C30	23.3	17.9	25.0	25.2	15.3	28.4	24.4	24.3	24.4	33.2	33.2	33.0	28.2	20.7	25.1	24.8	26.1	24.9
						C31	C1'		Glc		C31	C31	C31	CO-	CO-			
						16.2	102.7		C1'		51.9	170.7	15.5	OCH_3_	OCH_3_			
						C32	C2'		95.8			C32	C32	51.4	52.0			
						24.3	71.9					20.7	24.8					
NO.	**136^b)^**	**137^b)^**	**138^b)^**	**139^b)^**	**140**	**141**	**142^c)^**	**143^b)^**	**144^b)^**	**145^b)^**	**148^a)^**	**149^e)^**	**150^b)^**	**151^g)^**	**152^b)^**	**153^c)^**	**155^b)^**
C1	34.7	34.8	34.7	34.7	35.9	35.6	37.2	36.3	37.2	39.2	34.1	31.1	35.2	30.5	33.7	30.5	35.9
C2	24.2	24.1	24.2	24.2	33.6	27.6	35.2	34.0	34.5	18.2	28.5	23.6	24.1	24.6	27.4	23.0	33.8
C3	80.2	79.3	80.2	80.2	216.4	78.2	217.5	214.2	214.5	41.3	78.3	77.8	80.8	78.8	77.6	78.2	215.3
C4	37.8	37.9	37.7	37.8	46.0	41.6	49.0	47.8	47.4	38.3	39.4	37.0	37.8	38.1	40.5	36.5	47.7
C5	49.2	49.9	49.5	49.2	40.5	39.1	53.6	52.8	50.9	59.5	52.4	45.8	50.3	46.9	50.8	45.1	54.4
C6	26.7	37.0	26.7	26.7	23.1	22.2	40.9	40.0	33.4	22.3	19.1	18.3	26.3	19.4	36.2	17.7	40.3
C7	66.9	202.8	66.9	66.9	62.4	62.6	215.6	212.8	198.2	32.4	22.5	26.4	18.0	27.3	199.3	25.8	204.8
C8	157.0	154.7	157.0	157.0	63.6	62.8	55.0	54.0	150.0	42.8	51.2	134.7	132.9	135.6	146.8	133.9	46.2
C9	142.8	149.9	142.8	142.8	167.7	158.2	60.4	59.5	149.5	53.1	44.9	134.9	135.4	136.3	151.4	134.3	59.1
C10	38.9	40.1	38.9	38.9	40.5	37.9	38.0	36.5	39.2	37.5	35.7	37.2	37.0	38.4	39.1	36.6	37.5
C11	197.9	199.5	197.9	197.5	130.0	128.7	210.1	207.6	197.0	22.3	27.1	21.0	20.8	22.2	199.8	21.0	207.7
C12	50.4	52.3	50.4	50.4	203.6	78.1	53.7	52.6	192.6	41.5	49.0	29.6	31.1	30.8	49.9	28.6	50.6
C13	45.6	48.1	45.6	45.6	50.2	50.2	51.0	50.0	59.0	42.1	145.2	46.2	44.7	45.8	44.9	45.6	46.2
C14	59.6	53.0	59.6	59.6	64.8	64.4	51.3	49.7	61.0	59.5	47.9	48.8	51.0	51.0	57.2	49.3	54.7
C15	217.6	72.3	217.6	217.6	78.4	204.0	75.5	74.1	203.8	35.6	33.8	43.6	76.0	28.3	207.7	42.1	210.4
C16	41.1	36.5	41.1	41.1	127.2	127.2	39.0	38.4	38.9	30.5	31.3	76.6	36.5	28.7	35.0	76.6	40.2
C17	45.7	48.3	45.8	45.7	167.7	168.9	49.0	47.7	38.3	53.1	137.3	57.3	49.0	48.6	47.8	56.1	44.5
C18	17.6	17.5	17.6	17.6	30.9	31.0	17.1	16.6	12.4	14.6	179.2	17.8	16.1	17.0	17.8	17.0	15.8
C19	18.7	18.0	18.7	18.7	24.7	25.7	13.7	13.1	18.5	18.6	19.7	19.0	19.1	20.0	17.8	21.4	13.0
C20	32.2	32.6	32.2	32.2	72.2	72.2	33.5	32.0	32.2	36.0	35.4	48.7	36.2	49.0	73.9	47.9	32.1
C21	19.9	19.6	19.9	19.9	28.6	28.7	20.0	19.4	23.3	17.9	18.6	178.8	18.2	178.1	26.5	179.7	19.4
C22	49.5	49.5	49.5	49.5	53.9	53.9	50.5	49.6	48.6	35.3	35.1	31.6	34.6	34.3	42.1	32.0	49.1
C23	207.8	208.5	207.8	207.8	208.0	208.7	211.2	208.3	207.3	22.3	23.2	33.2	25.9	32.2(24R)	23.4	24.9	207.7
C24	46.8	46.8	46.9	46.8	48.0	48.2	47.8	46.8	46.5	58.2	125.6	156.1	145.2	76.5(24R)	143.6	33.6	47.1
C25	34.6	34.8	35.0	34.6	34.1	34.5	36.1	34.6	33.6	213.2	131.5	34.1	126.7	150.0(24R)	127.6	155.1	34.5
C26	179.9	179.3	175.8	179.9	180.1	179.6	179.6	176.2	180.3	32.1	25.8	21.9	172.6	110.8(24R)	173.0	106.4	176.2
C27	17.1	17.1	17.4	17.1	16.8	16.9	17.8	17.1	16.8	31.8	17.7	21.9	12.0	18.7(24R)	12.1	18.6	16.7
C28	28.3	17.3	28.3	28.3	24.0	24.6	25.9	25.2	--	20.3	26.7	27.8	18.2	28.7	27.9	27.3	25.4
C29	16.7	20.3	16.7	16.7	21.7	22.2	21.9	21.2	--	35.0	16.4	22.0	27.9	22.9	15.5	21.5	20.7
C30	24.5	28.3	24.6	24.5	26.9	27.0	12.8	12.5	27.4		28.7	25.7	16.5	25.3	22.4	24.8	12.7
	CO	CO						OCH_3_	C31			C31	CH_3_CO	COCH_3_			
	171.1	170.9						51.9	20.3			107.0	171.1	171.3			
	CH_3_	CH_3_							C32			CH_3_CO	CH_3_CO	COCH_3_			
	21.5	21.4							19.3			21.1	171.1	21.8			
**NO.**	**156^b)^**	**157^d)^**	**158^d)^**	**160^b)^**	**161^d)^**	**162^b)^**	**163^b)^**	**164**	**165**	**166**	**167**	**168^b)^**	**170^b)^**	**171**	**172^b)^**	**173^b)^**	**174^b)^**	**175^b)^**
C1	36.6	29.8	29.7	36.6	35.8	30.9	30.7	36.6	35.8	35.4	35.3	36.4	30.6	36.9	30.8	29.8	30.5	36.6
C2	34.8	25.7	25.6	34.9	34.8	23.2	23.2	34.8	28.2	28.1	24.1	34.7	23.1	35.0	23.3	25.5	23.0	34.8
C3	216.6	73.8	73.8	216.9	216.6	78.1	78.1	217.0	79.0	80.8	80.6	216.4	78.1	215.2	78.2	76.0	78.0	216.9
C4	47.4	37.2	37.0	47.5	47.3	36.6	36.6	47.4	38.7	38.0	37.4	47.4	36.5	47.5	36.8	37.1	36.4	47.5
C5	50.5	42.8	42.8	50.7	50.4	44.0	44.1	50.7	50.4	50.3	48.8	50.6	44.0	51.0	44.1	43.1	44.0	50.7
C6	23.6	22.6	22.6	23.7	23.6	22.9	22.8	23.6	23.1	24.3	22.7	23.6	22.8	23.9	22.7	27.5	27.5	23.7
C7	121.2	120.6	120.6	119.9	121.0	121.2	121.3	119.8	120.3	120.0	120.9	121.7	121.6	121.6	121.6	120.3	120.2	120.3
C8	141.0	142.0	141.9	142.9	141.0	140.3	140.8	142.8	142.7	142.6	140.0	139.9	140.5	142.1	140.2	142.2	142.2	142.5
C9	144.8	146.0	145.9	144.5	144.7	145.9	146.2	144.5	145.9	145.6	145.5	145.0	146.2	145.4	146.2	145.9	145.8	144.6
C10	37.3	37.1	37.1	37.2	37.2	37.4	37.4	37.2	37.4	37.2	37.2	37.4	37.3	37.6	37.5	37.3	37.1	37.2
C11	116.9	115.1	115.1	117.3	117.0	115.7	115.7	117.2	116.3	116.5	116.0	116.8	115.3	117.3	115.5	115.5	115.5	116.9
C12	38.5	35.3	35.3	37.8	38.5	38.0	38.5	37.8	37.9	37.8	37.9	36.6	38.4	38.9	38.1	37.6	37.6	37.8
C13	44.4	43.9	43.9	43.8	44.3	44.2	44.5	43.7	43.8	43.8	43.9	44.3	44.3	44.6	44.1	43.6	43.6	43.7
C14	52.0	48.5	48.4	50.3	51.9	51.5	52.1	50.3	49.2	49.3	51.2	42.9	52.1	52.6	51.6	50.3	50.3	50.3
C15	74.6	43.4	43.3	31.5	74.6	77.5	74.7	27.9	28.0	27.9	77.2	47.0	74.5	73.7	77.4	31.2	31.3	31.4
C16	40.0	75.1	75.0	27.9	40.1	37.0	40.1	31.4	31.5	31.5	36.8	219.2	39.6	40.5	36.7	22.9	22.8	27.6
C17	48.9	56.2	56.2	50.8	48.8	48.9	48.9	50.9	50.9	50.8	48.7	60.5	45.5	49.4	45.6	47.3	47.3	47.4
C18	16.0	16.9	16.9	15.7	15.9	16.0	16.0	15.7	15.7	15.7	15.8	16.8	15.8	16.5	15.9	15.4	15.4	15.5
C19	22.2	22.7	22.7	22.1	22.1	22.7	22.5	22.4	22.8	22.9	22.7	22.1	22.7	22.2	23.0	22.7	22.4	21.1
C20	35.9	46.9	46.9	36.2	35.8	36.0	36.0	36.0	36.1	36.1	35.8	31.5	39.3	36.3	39.8	39.3	39.3	39.4
C21	18.3	177.2	177.2	18.3	18.3	18.5	18.3	18.4	18.4	18.4	18.0	18.6	12.8	18.7	12.8	12.6	12.6	12.7
C22	34.7	31.9	31.7	35.8	36.6	34.7	34.8	35.9	36.0	35.8	34.5	35.7	74.7	30.0	74.6	74.6	74.6	74.7
C23	25.8	26.0	25.4	26.9	25.4	26.0	25.9	24.5	24.6	24.8	25.8	24.8	31.7	24.7	32.1	31.7	31.8	31.9
C24	145.1	124.3	123.1	147.1	126.6	144.9	145.2	126.9	127.0	128.6	144.9	131.2	139.1	127.6	139.2	139.5	139.6	139.5
C25	126.8	131.2	135.7	125.7	134.6	126.8	126.8	134.3	134.3	137.4	126.7	137.3	129.4	140.8	129.4	129.0	129.0	129.1
C26	172.2	25.7	66.5	172.1	69.0	172.0	172.1	69.0	69.1	66.8	173.0	67.7	172.2	65.5	172.0	172.2	172.3	171.9
C27	12.0	17.7	13.6	20.6	13.5	12.1	12.1	13.6	13.7	59.8	11.8	60.5	12.3	58.6	12.4	12.2	12.2	12.3
C28	17.0	28.7	28.7	25.4	16.9	27.8	27.8	25.3	25.6	25.6	18.2	22.4	27.8	25.7	--	--	--	25.3
C29	25.4	22.8	22.8	25.3	25.4	22.5	22.7	25.4	27.8	28.2	28.0	25.3	22.5	22.4	--	--	--	22.5
C30	22.5	26.2	26.1	22.5	22.1	18.2	17.2	22.0	15.8	17.0	16.8	25.7	17.3	18.0	27.9	28.1	27.7	25.5
						COCH_3_	COCH_3_				AcCH_3_		COCH_3_		C31	C31	C31	22-O-COCH_3_
						171.2	170.8				21.1		170.8		22.6	22.5	22.5	170.7
						COCH_3_	COCH_3_				AcCH_3_		COCH_3_		C32	C32	C32	22-O-COCH_3_
						21.4	21.3				21.2		21.3		18.6	25.7	25.6	21.1
**NO.**	**176^b)^**	**177^b)^**	**178^b)^**	**179^b)^**	**180^b)^**	**181^b)^**	**182^b)^**	**183^b)^**	**184^b)^**	**185^b)^**	**186^b)^**	**187^a)^**	**188^a)^**	**189^b)^**	**190^b)^**	**191^b)^**	**192^b)^**	**193^b)^**
C1	35.4	29.9	30.5	30.6	30.5	35.6	35.3	36.6	36.6	29.9	30.6	36.8	36.8	35.6	36.1	35.8	35.4	30.5
C2	24.2	25.5	23.0	23.1	23.1	27.6	24.1	37.5	34.8	25.5	23.1	35.0	29.2	27.8	34.9	36.5	36.5	23.1
C3	80.7	76.7	78.0	78.1	78.0	78.8	80.7	216.6	216.4	76.1	78.1	215.3	78.4	78.8	216.8	216.5	216.5	78.0
C4	37.6	37.3	36.4	36.5	36.4	38.5	37.4	47.4	47.4	37.0	36.5	47.5	39.8	38.6	47.5	47.6	47.6	36.4
C5	48.9	42.9	43.8	44.0	43.9	48.7	48.9	50.4	50.4	42.9	45.0	50.9	50.1	49.0	50.8	60.7	60.8	44.2
C6	22.8	23.0	22.7	22.8	22.7	22.9	22.6	23.6	23.7	23.0	22.8	23.8	23.9	22.9	23.7	23.8	23.8	22.7
C7	121.3	121.5	121.3	121.4	121.2	121.3	121.0	121.0	121.3	121.5	121.6	121.6	122.5	120.3	120.0	121.8	121.8	121.5
C8	140.0	140.0	140.0	140.6	140.6	140.0	140.6	140.4	140.2	140.0	140.5	142.0	142.4	142.4	142.9	140.1	140.2	140.4
C9	145.8	146.0	145.8	146.1	146.1	145.8	145.7	145.0	144.7	146.0	146.2	145.3	147.4	145.9	144.6	145.1	145.1	146.1
C10	37.3	37.3	37.3	37.3	37.2	37.3	37.2	37.3	37.3	37.3	37.3	37.5	38.3	37.3	37.3	37.6	37.6	37.2
C11	115.9	115.5	115.4	115.5	115.4	115.8	116.0	116.9	116.7	115.3	115.3	117.2	116.6	116.0	117.3	117.0	117.0	115.2
C12	38.0	37.9	37.8	38.3	38.3	37.9	38.3	38.0	38.0	37.9	38.4	38.8	39.2	37.7	37.9	36.8	36.8	38.4
C13	43.9	44.1	44.1	44.5	44.3	44.0	44.2	44.1	43.9	43.9	44.2	44.5	45.0	43.7	43.8	44.5	44.5	43.9
C14	51.3	51.7	51.4	52.2	52.0	51.2	51.8	51.3	51.4	51.4	52.0	52.6	53.0	50.2	50.4	43.1	43.1	52.0
C15	77.0	77.0	77.2	74.6	74.5	77.3	74.5	77.2	76.7	76.9	74.5	73.6	74.2	31.4	27.9	34.9	34.9	74.4
C16	36.6	37.2	37.1	40.1	40.2	36.9	39.8	37.0	36.6	36.7	39.8	40.9	41.0	27.7	31.5	25.0	25.3	39.6
C17	45.4	48.7	48.6	48.8	49.2	48.7	48.7	48.9	45.5	45.4	45.0	50.1	49.8	50.7	50.9	31.6	31.7	45.4
C18	15.7	16.0	15.9	16.0	15.7	15.9	15.8	16.0	15.8	15.8	15.8	16.3	16.8	15.7	15.8	17.0	17.1	15.7
C19	22.8	22.6	22.5	22.7	22.5	22.7	22.7	22.4	22.1	22.7	22.6	22.1	23.6	22.6	22.5	22.3	22.3	22.6
C20	39.6	32.8	32.8	33.0	33.4	35.8	35.8	35.9	39.6	39.6	39.3	34.4	36.8	36.0	36.1	50.8	50.8	39.2
C21	12.6	19.4	19.3	19.6	19.4	18.1	18.1	18.2	12.7	12.7	12.8	20.3	19.0	18.2	18.4	18.7	18.8	12.7
C22	74.4	51.5	51.5	51.9	67.0	34.5	34.7	34.6	74.4	74.4	74.6	44.7	37.3	34.6	36.7	219.3	219.0	74.5
C23	31.9	201.6	201.4	201.8	43.6	25.8	25.7	25.9	31.9	31.9	31.7	67.1	25.1	25.9	24.4	47.1	47.1	31.7
C24	139.0	133.8	133.9	134.1	144.8	144.9	145.0	144.5	139.0	139.1	139.2	145.5	127.9	155.4	131.7	131.4	126.8	139.2
C25	129.2	139.5	139.4	139.3	128.3	126.7	126.8	126.8	129.2	129.2	129.4	128.7	141.3	139.1	136.8	137.4	134.8	129.2
C26	171.3	171.2	171.8	171.0	172.0	172.9	172.9	172.1	171.3	171.6	172.0	170.3	65.8	195.3	67.7	60.2	69.3	172.1
C27	12.3	14.1	13.9	14.1	12.6	11.9	11.9	12.0	12.3	12.3	12.3	13.5	58.8	9.0	60.2	67.9	13.8	12.2
C28	18.4	18.5	18.3	17.2	17.1	18.3	17.0	18.2	25.4	--	--	25.6	29.3	25.4	25.5	25.5	25.5	17.2
C29	28.1	28.2	27.7	27.8	27.7	28.1	28.0	25.4	22.4	--	--	22.3	17.1	28.0	22.1	22.6	22.6	27.6
C30	16.9	22.6	22.3	22.5	22.3	15.7	16.8	22.1	18.3	28.2	27.7	18.0	18.6	15.5	25.4	25.8	25.8	22.4
	AcCO	AcCO	AcCO	AcCO	AcCO	AcCO	AcCO	AcCO	15-O-	C31	C31							C1'
	171.1	171.0	171.1	170.9	170.7	171.1	170.9	171.2	COCH_3_	22.8	22.5							170.8
	AcCO	AcCH_3_	AcCO	AcCH_3_	AcCH_3_	AcCO	AcCO	AcCO	171.1	C32	C32							C2'
	170.6	21.4	170.7	21.3	21.2	21.3	21.2	21.4		18.5	17.3							170.5
										AcCH_3_	AcCH_3_							C3'
										21.0	21.6							21.2
**NO.**	**194^b)^**	**196^d)^**	**197^b)^**	**198^b)^**	**201^b)^**	**202^b)^**	**203**	**204^b)^**	**205^b)^**	**207^b)^**	**208^b)^**	**209**	**210^b)^**	**214^b)^**	**215^b)^**	**216^b)^**	**217^b)^**	**218^b)^**
C1	35.6	36.1	35.7	35.4	35.7	36.6	36.6	36.5	29.9	30.0	35.5	30.5	36.7	29.8	35.6	30.6	35.4	29.9
C2	27.7	34.4	28.0	22.8	27.9	34.8	28.7	34.9	25.6	25.6	27.0	23.0	34.8	25.4	27.4	23.1	24.2	25.6
C3	78.8	215.3	78.9	80.8	78.9	216.7	78.1	216.9	76.1	76.3	78.3	78.0	217.0	75.8	78.6	78.0	80.7	76.1
C4	38.6	46.9	38.7	37.6	38.7	47.4	39.4	47.5	37.3	37.3	38.3	36.4	47.5	37.2	38.5	36.5	37.6	37.4
C5	48.7	50.5	49.1	49.3	49.1	50.7	49.8	50.3	42.9	43.1	48.5	43.8	50.3	42.9	49.0	43.9	49.0	43.0
C6	22.9	23.2	23.0	24.3	23.0	23.6	23.5	23.7	23.0	23.0	22.6	22.7	23.6	22.8	22.8	22.8	22.9	22.9
C7	121.6	120.1	120.4	120.0	120.2	119.9	121.0	119.9	121.3	121.4	121.0	121.0	119.9	121.2	121.2	121.2	121.2	121.7
C8	139.9	142.1	142.5	142.7	142.6	142.8	143.0	142.8	140.2	140.9	140.5	140.1	142.8	140.6	140.7	140.1	140.2	140.5
C9	146.0	144.2	146.0	146.7	145.9	144.5	146.6	144.5	146.0	146.3	145.9	145.4	144.4	146.1	146.0	145.9	145.7	146.3
C10	37.4	37.0	37.4	37.8	37.3	37.2	37.8	37.8	37.3	37.3	37.1	37.2	37.8	37.2	37.3	37.3	37.3	37.4
C11	115.5	116.7	116.1	116.5	116.2	117.2	116.6	117.2	115.6	115.7	115.5	115.5	117.2	115.4	115.8	115.5	116.1	115.3
C12	37.9	35.3	37.8	37.2	37.8	37.8	38.1	37.2	38.0	38.5	38.2	37.9	37.2	38.3	38.4	37.9	38.0	38.5
C13	43.9	43.8	43.8	43.7	43.7	43.7	44.1	43.8	44.1	44.4	44.0	44.0	43.7	44.2	44.1	44.1	44.1	44.2
C14	51.3	48.3	50.3	50.3	50.3	50.3	50.7	50.7	51.4	52.2	51.6	51.3	50.7	51.9	51.9	51.4	51.4	52.1
C15	76.9	43.3	31.5	26.0	31.5	27.6	28.1	27.9	77.4	74.8	73.9	77.3	27.9	74.2	74.3	77.3	77.1	74.6
C16	36.6	75.0	27.8	31.5	27.8	28.8	31.9	28.7	37.0	40.0	39.2	36.9	28.8	39.8	38.7	37.2	37.2	39.8
C17	45.4	56.2	50.9	50.8	50.9	51.0	51.5	50.9	48.9	48.8	48.7	48.8	51.0	49.2	45.0	49.4	49.4	45.4
C18	15.7	16.9	15.7	16.9	15.7	15.7	16.6	15.7	16.0	15.9	15.5	15.9	15.3	15.7	15.7	15.9	15.9	15.8
C19	22.7	21.8	22.7	22.8	22.7	22.4	23.1	22.5	22.7	22.7	22.4	22.5	22.4	22.6	22.7	22.6	22.9	22.7
C20	39.5	46.9	36.2	36.5	36.5	36.5	37.1	36.6	36.0	35.9	35.6	35.9	36.6	33.3	40.7	33.6	33.6	39.3
C21	12.6	177.2	18.3	18.6	18.6	18.6	19.0	18.6	18.2	18.3	17.9	18.1	18.6	19.3	12.4	19.3	19.3	12.8
C22	74.4	31.8	34.7	32.6	33.5	31.4	34.4	31.5	34.7	34.8	34.5	34.5	31.5	66.5	72.1	67.2	67.2	74.6
C23	31.8	26.0	26.1	27.8	28.7	33.5	28.9	33.5	25.9	25.7	25.4	25.8	33.5	43.4	34.8	43.3	43.4	31.7
C24	138.9	124.2	155.4	76.6	79.6	79.2	77.2	79.6	145.1	145.2	143.3	145.0	79.1	143.6	139.9	144.7	144.7	139.2
C25	129.2	131.1	139.1	73.3	73.2	73.9	74.8	73.3	126.6	127.0	127.0	126.7	74.1	128.6	129.0	128.1	128.1	129.1
C26	171.5	25.6	195.4	68.5	23.6	67.6	69.3	25.5	171.9	172.8	170.5	172.9	67.6	170.3	170.4	171.2	171.2	171.0
C27	12.2	17.6	9.2	20.2	26.5	22.0	20.1	25.3	12.0	12.0	11.7	11.8	22.0	12.7	11.4	12.8	12.8	12.3
C28	18.3	25.4	28.1	28.1	28.1	--	28.8	23.2	18.5	17.4	16.8	18.3	25.4	17.0	17.1	18.4	18.4	17.3
C29	28.1	22.1	15.8	15.7	15.8	--	16.0	26.6	28.2	28.2	27.7	27.7	25.4	28.0	28.0	27.8	28.1	28.1
C30	15.7	25.8	25.6	25.5	25.6	25.3	25.9	22.1	22.8	22.8	15.4	22.3	20.9	22.7	15.7	22.4	16.9	22.8
	C1'			OCOCH_3_		C31			C31			AcCH_3_				CH_3_CO	CH_3_CO	CH_3_CO
	171.0			170.64		20.9			171.2			21.2				170.8	170.5	170.6
	C2'			170.93		C32			C32			AcCH_3_				CH_3_CO	CH_3_CO	CH_3_CO
	170.5			171.11		25.4			21.4			21.3				170.6	171.0	21.03
	C3'			OCOCH_3_								AcCO				CH_3_CO	CH_3_CO	
	21.3			20.83								170.7				21.4	21.3	
	C4'			20.99								AcCO				CH_3_CO	CH_3_CO	
	20.9			21.29								171.0				21.3	21.4	
**NO.**	**219^b)^**	**220^b)^**	**221^b)^**	**222^b)^**	**223^b)^**	**224^b)^**	**225^b)^**	**226^b)^**	**227^b)^**	**228^b)^**	**229^b)^**	**230^b)^**	**231^b)^**	**232^b)^**	**233^b)^**	**234^b)^**	**238^b)^**	**239^b)^**
C1	35.6	36.6	35.7	35.0	35.8	34.7	35.8	37.4	35.7	36.0	34.8	37.4	36.7	37.3	35.6	34.2	35.3	34.4
C2	27.3	34.8	27.8	27.9	34.5	27.5	34.1	34.0	34.3	34.5	27.6	34.0	27.4	34.6	34.2	23.9	34.3	27.2
C3	78.6	216.8	78.9	78.5	216.8	78.3	216.6	214.8	216.5	215.9	78.3	215.2	77.5	215.3	218.1	79.9	214.7	78.0
C4	38.5	47.5	38.7	38.8	47.0	38.6	46.7	46.9	46.8	46.8	38.6	46.9	40.5	43.9	46.7	37.5	47.2	38.5
C5	48.9	50.7	49.1	49.3	49.1	49.1	48.8	51.0	49.0	48.9	49.1	50.9	51.4	51.0	48.7	49.2	50.4	49.1
C6	22.8	23.7	23.0	26.8	27.8	26.5	27.6	33.8	27.7	29.1	26.6	33.7	33.3	33.9	27.6	26.6	37.1	26.6
C7	121.6	120.0	120.3	67.0	66.5	66.9	66.2	198.5	66.3	65.7	66.9	198.7	198.8	199.5	66.2	66.1	198.1	66.1
C8	140.4	142.8	142.6	157.2	158.1	156.8	157.4	149.7	157.8	159.8	156.8	149.8	151.6	149.7	157.9	157.4	139.5	155.9
C9	146.2	144.5	146.0	142.9	141.4	142.6	141.1	146.2	141.3	140.9	142.7	146.2	146.0	146.9	141.1	141.6	162.7	142.9
C10	37.3	37.2	37.4	38.8	38.4	38.8	38.2	39.3	38.3	38.5	38.8	39.3	39.2	39.4	38.2	37.5	39.4	38.5
C11	115.4	117.2	116.2	198.2	197.9	197.8	196.9	194.1	197.5	198.0	198.0	194.1	194.1	199.4	197.8	199.5	23.8	192.3
C12	38.3	37.8	37.8	50.6	50.4	49.1	49.9	79.1	49.1	49.8	50.3	79.1	79.4	49.0	50.2	78.2	30.1	79.8
C13	44.0	43.8	43.8	45.5	45.1	46.0	45.0	47.6	45.3	45.5	45.3	47.6	48.0	47.0	44.9	51.9	44.9	50.4
C14	51.8	50.3	50.3	59.6	59.5	58.8	59.2	58.6	58.8	58.3	59.4	58.6	58.5	57.2	59.3	60.2	47.8	60.6
C15	74.0	31.5	31.5	218.1	218.2	217.7	215.8	205.8	217.7	215.5	217.5	205.9	206.0	207.3	216.8	217.1	28.5	216.7
C16	39.1	27.9	27.9	41.2	41.3	38.4	35.6	37.4	38.7	39.5	41.0	37.6	37.6	39.9	41.1	38.0	31.8	37.4
C17	45.3	50.9	50.9	46.4	46.5	46.1	49.5	45.2	46.3	46.7	46.1	45.2	45.5	45.2	46.2	46.5	48.9	46.0
C18	15.6	15.7	15.7	17.6	17.9	18.4	19.0	12.0	18.8	19.3	17.4	12.0	12.1	16.1	17.6	12.0	15.9	13.1
C19	22.7	22.5	22.7	18.6	18.3	18.5	18.1	18.6	18.2	18.6	18.4	18.7	18.0	18.6	18.2	18.8	17.9	18.6
C20	39.1	36.1	36.0	35.4	35.4	143.9	85.9	33.0	143.9	145.8	35.1	33.1	33.0	35.4	35.2	31.7	35.9	31.8
C21	12.5	18.4	18.4	18.3	18.2	112.2	25.9	20.1	112.3	111.6	18.0	20.0	20.2	18.3	18.0	20.5	18.3	20.4
C22	74.9	32.7	32.7	30.8	30.9	31.3	34.2	30.1	31.3	31.8	30.4	29.9	30.2	30.8	30.6	29.4	30.8	29.5
C23	31.5	25.3	25.3	31.3	31.3	32.3	27.4	31.6	31.9	32.6	30.7	31.6	31.8	31.0	31.1	31.5	30.9	30.0
C24	137.2	60.6	60.6	173.8	173.8	177.3	175	173.6	175.1	173.2	178.2	178.7	173.7	173.8	173.5	177.8	178.1	178.2
C25	129.9	60.7	60.7	--	--	--	--	--	--	--	--	--	--	--	--	--	--	--
C26	169.9	65.4	65.4	--	--	--	--	--	--	--	--	--	--	--	--	--	--	--
C27	12.3	14.2	14.2	--	--	--	--	--	--	--	--	--	--	--	--	--	--	--
C28	17.1	25.4	28.1	28.4	27.2	24.2	25.0	--	27.0	27.0	24.4	--	--	--	--	27.9	25.3	28.0
C29	27.9	22.1	15.8	15.6	20.9	28.1	26.9	--	20.8	20.9	28.1	--	--	--	--	16.4	21.4	15.3
C30	15.6	25.4	25.6	24.6	24.9	15.4	20.7	27.6	24.6	25.2	15.4	27.6	27.9	27.7	27.0	23.1	24.9	24.0
	CH_3_CO			Bu1'	Bu1'			C31		COOCH_3_		C31	C31	C31	C31	C31		COCH_3_
	171.1			64.6	64.6			20.8		51.5		20.4	15.6	20.9	20.7	161.0		170.5
	CH_3_CO			Bu2'	Bu2'			C32				C32	C32	C32	C32			COCH_3_
	20.87			30.9	30.9			20.4				20.8	21.4	20.3	24.6			20.7
**NO.**	**241^b)^**	**242^b)^**	**243^b)^**	**244^b)^**	**248^b)^**	**249^b)^**	**252^b)^**	**253^b)^**	**254^b)^**	**255^b)^**	**256^b)^**	**261^b)^**	**262^a)^**	**263^a)^**	**264^a)^**	**266^a)^**	**267^b)^**	**268^a)^**
C1	35.6	33.6	34.0	33.3	35.0	35.4	34.0	34.6	33.2	34.8	34.4	36.6	36.8	35.4	36.7	35.3	35.4	73.8
C2	34.3	27.4	33.8	27.4	27.9	34.5	33.6	33.8	27.3	27.6	27.4	34.8	35.0	28.3	27.9	28.9	27.7	39.8
C3	216.9	77.6	214.8	77.5	78.5	215.9	214.9	215.1	77.4	78.2	78.1	216.5	215.3	72.0	70.7	79.3	78.8	75.5
C4	46.8	39.1	46.9	40.5	38.8	47.2	46.9	47.0	40.5	38.6	38.6	47.4	47.4	43.0	43.6	43.1	38.8	40.2
C5	48.8	50.8	51.0	51.4	49.3	49.6	50.9	50.9	51.4	49.1	49.1	50.7	50.9	42.5	44.5	50.2	50.2	49.1
C6	29.0	36.3	37.4	36.7	26.9	28.0	37.4	37.2	36.6	26.6	26.7	23.7	23.8	27.8	34.1	28.5	18.2	17.6
C7	68.8	199.4	198.5	198.8	67.0	66.0	198.4	199.1	198.0	66.0	66.1	119.9	121.6	66.7	200.4	67.0	26.4	26.0
C8	159.2	151.6	149.7	151.6	157.2	158.6	145.6	146.2	151.4	156.5	155.9	142.6	142.0	158.8	152.2	158.0	134.1	134.1
C9	140.3	147.0	146.2	156.0	142.9	140.6	149.6	149.6	145.4	142.6	142.9	144.4	145.3	142.9	147.4	142.6	134.4	137.0
C10	38.0	40.4	39.3	39.2	38.9	38.1	39.3	34.6	39.1	38.9	38.6	37.2	37.5	39.2	40.8	39.1	36.9	44.1
C11	199.6	199.9	194.1	194.1	198.1	199.9	193.3	198.0	193.0	197.0	191.0	117.0	117.2	198.3	199.8	198.4	20.9	25.1
C12	51.8	49.6	79.1	79.4	50.6	78.5	78.6	48.8	78.9	50.2	79.1	37.8	38.7	51.1	49.9	51.1	30.7	32.0
C13	46.6	44.3	47.6	48.0	45.5	51.9	47.9	44.3	48.2	45.4	49.8	43.7	44.5	45.6	44.5	45.6	44.4	44.3
C14	53.9	57.0	58.6	58.5	59.6	60.5	58.9	57.1	58.9	59.3	61.0	50.2	52.6	59.2	57.6	59.0	49.8	50.4
C15	72.6	208.1	205.8	206.0	218.1	217.5	203.8	204.9	203.9	215.7	214.5	27.9	73.6	216.8	208.0	216.7	30.7	31.6
C16	36.6	40.2	37.4	37.6	41.2	37.9	35.4	34.3	35.7	35.7	37.1	31.5	40.4	41.4	40.5	41.5	27.7	28.7
C17	48.5	45.6	45.2	45.5	46.4	46.8	48.8	48.1	49.2	49.5	50.2	50.8	49.3	46.2	45.5	46.3	45.7	46.6
C18	17.3	16.2	12.0	12.1	17.6	12.3	13.0	17.4	13.2	18.8	14.2	15.7	16.4	17.8	16.4	17.9	15.5	16.2
C19	19.4	17.8	18.6	18.0	18.6	18.5	18.7	18.6	17.9	18.3	18.5	22.0	22.1	19.2	18.6	19.2	19.1	15.5
C20	35.7	35.3	33.0	33.0	35.4	31.8	86.4	86.0	86.6	85.9	86.7	36.2	36.1	35.4	35.5	35.3	40.4	40.7
C21	18.1	18.2	20.1	20.2	18.2	20.7	26.1	26.3	26.1	25.9	25.2	18.3	18.4	18.1	18.2	18.0	13.3	13.8
C22	30.0	30.5	30.1	30.2	30.9	30.0	34.5	34.2	34.5	34.2	34.6	29.7	32.0	31.0	31.0	31.0	80.2	80.5
C23	31.0	30.8	31.6	31.8	31.1	32.4	28.0	27.3	28.1	27.5	28.3	30.1	31.9	31.0	31.1	30.9	27.7	28.7
C24	174.3	178.0	173.6	173.7	174.1	173.9	175.6	175.8	175.6	175.9	175.5	178.2	176.4	174.0	174.0	174.1	139.7	140.5
C25	--	27.8	--	--	--	--	--	--	--	--	--	--	--	66.6	65.1	--	128.1	127.8
C26	--	15.5	--	--	--	--	--	--	--	--	--	--	--	13.2	13.1	--	166.6	166.2
C27	--	21.8	--	--	--	--	--	--	--	--	--	--	--	24.8	21.6	--	17.1	18.7
C28	27.4	--	--	--	28.4	26.5	27.6	27.6	27.9	28.1	28.1	25.4	25.6	--	--	23.7	27.9	28.2
C29	20.7	--	--	--	15.6	21.4	20.4	20.3	15.5	15.4	15.4	22.4	22.3	--	--	64.2	15.4	15.4
C30	19.4	--	27.6	27.9	24.6	23.5	21.1	21.3	21.6	24.7	24.5	25.3	17.9	--	--	24.9	24.3	24.9
	CO-		C31	C31	C1'	C1'	COCH_3_		COCH_3_		COCH_3_			CO-	CO-	CO-		
	OCH_3_		20.4	15.6	51.9	64.6	170.1		170.1		170.3			OCH_3_	OCH_3_	OCH_3_		
	51.6		C32	C32		C2'	COCH_3_		COCH_3_		COCH_3_			51.4	51.4	51.4		
			20.8	21.4		30.9	21.0		21.0		21.2							
**NO.**	**269^b)^**	**270^b)^**	**271^b)^**	**272^b)^**	**273^b)^**	**274^b)^**	**275^b)^**	**276^b)^**	**277^a)^**	**278^b)^**	**279^b)^**	**280^b)^**	**281^b)^**	**282^b)^**	**286**	**287**	**288^b)^**	**289^b)^**
C1	143.7	143.6	144.2	147.9	35.2	35.0	36.6	36.6	35.5	35.2	36.0	35.7	35.7	35.4	36.2	36.1	36.7	29.7
C2	118.0	118.1	118.5	116.7	34.3	28.3	34.8	34.8	29.3	34.3	33.6	27.7	35.4	27.5	33.7	33.6	34.9	22.8
C3	167.1	167.0	166.8	163.9	213.2	77.0	217.0	217.0	78.1	217.2	216.2	78.0	215.7	77.9	216.5	216.0	216.9	77.5
C4	80.5	80.5	80.6	77.5	47.0	38.6	47.5	47.5	40.1	44.8	45.9	41.5	46.0	41.8	46.0	45.9	47.5	36.4
C5	49.0	49.0	49.2	92.6	49.8	49.6	50.8	50.8	50.5	49.4	40.8	39.3	40.7	39.4	39.4	40.7	50.7	47.7
C6	38.7	38.4	37.3	44.1	27.9	27.9	23.6	23.6	27.2	27.2	23.2	21.8	22.9	21.8	23.2	22.7	23.7	20.4
C7	27.6	27.2	27.2	26.8	65.8	66.6	119.4	119.4	71.7	70.1	60.5	58.7	59.1	59.0	62.9	62.6	119.4	25.7
C8	147.6	147.0	149.3	149.5	157.8	156.8	142.8	142.7	158.3	158.3	66.0	71.4	62.1	62.9	64.3	57.6	142.7	137.6
C9	130.5	129.9	131.8	127.6	140.9	143.0	144.4	144.4	140.7	138.7	163.4	165.6	167.6	168.9	164.8	164.8	144.3	128.3
C10	139.1	139.3	140.1	132.9	37.8	39.2	37.2	37.1	39.3	37.5	40.2	37.9	40.8	38.0	40.3	40.7	37.2	39.4
C11	26.9	26.8	67.7	28.0	199.4	200.5	117.3	117.2	200.0	199.7	130.8	128.7	129.8	128.5	130.1	129.7	117.2	22.5
C12	31.4	31.1	42.5	31.1	78.1	79.0	37.7	37.7	53.3	52.1	201.2	200.9	199.8	200.2	202.8	200.5	37.7	31.0
C13	43.7	44.3	43.2	44.3	49.9	50.5	43.7	43.7	40.1	46.9	50.3	54.9	54.5	54.3	53.3	55.0	43.8	44.3
C14	56.4	54.9	55.6	55.1	63.3	63.0	50.3	50.2	53.7	52.9	59.3	62.0	62.8	61.5	60.2	58.8	50.3	50.4
C15	76.2	78.0	77.6	78.4	215.5	215.0	27.8	27.8	71.9	70.8	71.7	209.6	202.8	203.1	76.6	209.5	27.8	31.1
C16	40.6	38.6	38.5	38.4	60.4	60.6	31.5	31.4	33.3	32.5	33.6	38.5	125.2	125.2	39.5	38.3	31.5	27.6
C17	45.6	45.3	45.1	45.7	54.2	54.0	50.8	50.7	49.6	48.9	45.8	43.0	181.6	181.9	48.7	42.9	50.9	45.7
C18	17.3	16.7	18.9	16.8	12.8	13.3	15.7	15.7	18.1	17.6	18.1	18.1	30.3	29.8	19.6	18.1	15.8	15.7
C19	143.0	142.8	141.0	139.4	18.3	19.2	22.0	22.0	20.7	19.6	21.6	18.3	24.9	25.9	21.3	21.6	22.1	104.0
C20	40.0	39.9	39.9	39.8	33.0	33.3	36.4	36.5	33.6	32.7	157.3	154.9	73.0	72.9	158.6	158.6	36.5	40.4
C21	13.4	13.3	13.2	13.3	19.2	19.6	18.5	18.5	20.3	19.8	20.6	20.4	29.5	29.4	20.9	20.4	18.7	13.3
C22	80.0	79.8	79.6	79.7	52.5	52.3	33.1	33.1	50.6	49.9	126.0	126.7	52.9	52.9	126.3	126.8	33.5	80.1
C23	27.8	27.8	27.7	27.7	87.0	87.5	25.8	25.7	209.3	208.5	198.8	198.7	206.2	206.3	199.2	198.5	29.8	27.9
C24	139.6	139.4	139.4	139.3	40.6	40.4	78.8	76.8	47.8	46.9	47.6	47.7	47.8	47.8	47.6	47.6	77.2	139.0
C25	128.3	128.4	128.4	128.4	34.3	34.8	73.6	73.5	36.3	34.8	34.8	34.9	34.5	33.1	33.7	34.9	73.9	128.0
C26	166.4	166.3	166.2	166.2	178.6	179.0	66.5	68.7	178.6	176.4	179.6	179.7	179.9	179.9	180.1	180.2	69.6	166.5
C27	17.1	17.1	17.1	17.1	15.6	15.6	18.1	18.3	18.3	17.3	17.0	17.0	16.9	16.9	17.0	17.0	20.3	17.2
C28	29.0	28.8	28.1	24.9	--	--	25.3	25.3	29.3	27.8	14.6	17.1	24.8	17.1	21.7	24.8	25.4	23.8
C29	26.3	26.4	26.7	24.8	--	--	22.5	22.4	17.4	20.8	24.6	25.8	21.6	25.7	24.8	21.7	22.5	25.7
C30	26.6	26.4	25.4	24.4	25.8	28.5	25.4	25.4	20.2	19.8	28.7	28.7	28.7	28.7	28.6	28.6	25.5	23.2
		C1'	C1'	C1'	C31	C31	C1'	C1'	C1'	COCH_3_							C1'	OCH_3_
		170.4	170.3	170.1	21.3	16.0	195.5	31.8	102.7	52.1							195.3	55.2
		C2'	C2'	C2'	C32	C32	C2'	C2'	C2'	C(CH_3_)_2_							C2'	
		21.4	21.4	21.3	25.2	24.6	127.8	140.6	24.1	102.3							126.6	
**NO.**	**290^b)^**	**291^b)^**	**292^b)^**	**293^a)^**	**294^b)^**	**295^b)^**	**296^b)^**	**297^e)^**	**298^b)^**	**299^a)^**	**300^a)^**	**301^a)^**	**302^b)^**	**303^b)^**	**304^b)^**	**305^b)^**	**306^b)^**	**307^b)^**
C1	27.5	143.8	34.1	35.5	35.7	31.2	28.7	31.3	32.6	57.0	57.0	57.0	30.4	31.0	28.8	28.8	29.7	35.2
C2	27.1	118.0	27.4	28.6	34.2	27.7	28.4	23.6	28.0	36.5	36.5	36.5	23.3	34.6	27.7	25.5	28.3	34.0
C3	177.3	167.0	76.5	77.6	216.4	78.7	179.0	78.3	78.9	216.5	216.5	216.4	78.1	216.6	175.6	174.5	175.5	214.9
C4	74.5	77.8	39.2	49.7	46.8	38.9	75.3	37.0	39.0	47.0	47.0	47.1	36.7	47.1	75.2	75.0	74.8	46.6
C5	55.1	48.9	50.3	49.7	48.9	50.2	47.9	46.0	50.1	55.4	55.4	55.5	45.3	39.7	47.9	48.0	47.8	49.3
C6	33.8	35.8	36.9	28.1	27.6	17.6	23.9	18.4	17.8	29.4	29.4	29.4	18.0	28.8	24.3	21.5	24.8	36.9
C7	27.1	27.1	203.8	66.7	66.2	25.7	26.7	26.3	26.2	68.3	68.4	68.3	25.9	66.2	24.3	117.9	26.0	204.5
C8	139.2	146.0	146.2	158.7	157.5	137.1	143.3	134.4	137.9	136.4	136.3	136.3	133.7	132.9	143.1	134.1	139.6	150.9
C9	121.7	147.0	155.9	142.7	141.2	131.7	126.1	135.1	130.4	154.7	154.7	154.8	134.7	76.3	126.1	141.1	126.1	153.2
C10	91.5	142.8	40.4	39.3	38.3	39.6	45.4	37.3	42.2	48.6	48.6	48.6	36.9	41.4	45.4	42.2	45.7	39.4
C11	33.0	26.3	198.5	198.0	196.8	23.0	20.8	21.2	22.0	83.5	83.6	83.5	20.8	26.2	22.6	120.4	22.5	200.9
C12	30.7	31.1	79.3	50.9	50.1	31.0	31.0	30.8	31.0	37.4	37.4	37.4	30.8	34.2	31.2	39.4	31.2	49.6
C13	44.5	44.3	54.1	45.8	45.1	44.4	44.3	45.0	44.4	45.6	45.6	45.7	44.3	45.2	51.4	43.7	44.1	48.2
C14	50.5	54.9	50.6	59.0	59.3	50.2	51.5	49.9	50.4	62.3	62.4	62.4	49.5	155.0	51.8	49.9	51.4	51.8
C15	30.1	77.8	80.1	215.0	215.8	30.7	31.3	27.5	30.7	215.0	215.3	214.8	27.0	77.5	30.5	31.5	30.8	72.9
C16	27.1	38.5	128.1	36.5	35.8	27.6	27.4	29.4	27.5	41.0	41.1	41.0	28.9	47.9	28.5	26.0	26.6	31.7
C17	45.5	44.8	157.7	49.7	49.0	45.8	45.9	47.7	47.1	46.8	46.9	46.6	47.0	49.7	46.9	47.5	47.8	39.2
C18	15.5	16.7	26.0	18.6	18.1	15.6	15.9	16.4	16.6	17.7	17.7	17.7	16.3	17.2	15.7	16.0	15.8	16.6
C19	41.5	142.8	20.8	19.4	19.1	67.8	67.3	19.1	65.8	18.8	18.8	18.8	19.0	17.0	67.2	61.6	67.2	17.6
C20	40.3	35.8	71.2	86.5	85.5	40.4	40.3	49.1	41.6	35.7	35.9	32.6	47.8	37.8	40.1	40.0	40.2	124.4
C21	13.3	12.8	32.8	25.8	26.0	13.3	13.4	178.5	12.0	18.6	18.7	20.3	175.2	13.3	12.9	12.6	13.3	138.4
C22	80.1	84.0	48.6	27.8	27.5	80.2	80.3	31.9	72.7	31.4	31.9	49.8	32.9	74.6	75.6	75.5	80.1	107.7
C23	27.9	63.6	106.8	34.2	34.2	27.9	27.8	32.8	34.0	31.1	31.8	208.9	123.5	31.6	33.1	33.1	27.3	153.6
C24	139.7	143.8	44.6	176.7	175.9	139.6	139.7	156.0	125.4	174.1	176.1	46.9	132.3	138.5	141.0	140.9	142.9	34.3
C25	128.0	127.7	34.1	--	--	128.2	128.2	34.3	137.9	--	--	35.7	155.4	129.8	128.2	128.1	128.0	38.5
C26	166.5	164.0	178.7	--	--	166.6	166.7	22.0	61.4	--	--	178.4	106.7	172.0	172.4	171.7	166.3	180.0
C27	17.1	16.8	14.5	--	--	17.1	17.1	21.9	22.4	--	--	17.7	17.7	12.5	20.6	20.5	17.0	18.9
C28	32.0	28.5	29.6	25.4	25.0	28.1	33.7	28.0	28.4	27.1	27.1	27.1	27.6	25.8	33.7	33.5	28.7	27.3
C29	25.2	26.6	15.1	16.5	27.0	15.5	26.1	21.9	15.5	18.9	18.9	18.9	21.8	22.0	26.1	25.8	23.8	20.4
C30	24.5	26.6	29.7	28.8	20.7	24.2	24.9	24.5	24.7	20.6	20.7	20.6	24.4	31.4	24.3	24.3	22.5	20.5
		CO				C1'	C1'	C1'	C1'	OCH_3_			C1'		C1'	C1'	C1'	
		170.4				171.1	170.7	171.2	170.5	51.4			94.5		170.6	170.5	170.4	
**NO.**	**308^a)^**	**309^b)^**	**310^a)^**	**311^b)^**	**312^a)^**	**315^a)^**	**316^b)^**	**NO.**	**308^a)^**	**309^b)^**	**310^a)^**	**311^b)^**	**312^a)^**	**315^a)^**	**316^b)^**
C1	32.7	31.8	38.0	36.6	37.1	35.5	32.4	C18	17.7	17.7	18.0	17.8	17.7	17.7	19.6
C2	30.8	30.4	29.6	29.9	30.1	28.8	32.2	C19	22.4	22.4	25.5	24.9	23.0	20.0	16.3
C3	176.5	173.9	176.4	174.7	175.6	77.6	214.1	C20	36.2	36.2	36.2	36.0	139.9	40.1	205.2
C4	146.5	146.4	87.3	86.1	145.0	39.4	47.0	C21	18.5	18.5	18.1	18.1	19.0	17.5	31.2
C5	45.0	45.0	48.0	48.4	44.8	49.9	43.5	C22	31.9	27.7	31.9	31.3	126.3	66.9	26.9
C6	35.5	35.4	32.1	32.5	27.0	29.0	36.7	C23	31.9	32.4	31.6	31.0	75.2	--	20.3
C7	67.9	67.8	72.9	73.1	61.3	69.5	198.3	C24	176.5	176.5	176.4	174.5	36.8	--	17.5
C8	165.3	165.7	161.4	161.2	66.5	160.5	66.8	C25	--	--	--	--	34.3	--	--
C9	137.4	137.1	135.4	135.1	163.0	141.9	68.1	C26	--	--	--	--	179.0	--	--
C10	41.3	41.2	41.6	41.1	43.8	39.1	37.4	C27	--	--	--	--	15.5	--	--
C11	200.4	200.4	199.8	200.2	130.0	200.3	200.6	C28	115.6	115.7	71.3	71.3	115.2	28.8	--
C12	52.1	52.0	50.9	50.8	201.1	52.8	46.0	C29	23.4	23.2	25.0	25.3	23.0	16.7	
C13	47.3	41.2	45.3	45.2	59.1	47.7	45.5	C30	27.6	27.7	24.5	23.8	15.1	20.2	
C14	53.4	53.3	50.9	50.6	51.8	54.5	54.8			CH_2_O		3-OCH_3_	OAc		
C15	32.4	31.9	30.1	29.3	71.8	72.6	205.9			60.4		51.9	20.7		
C16	27.8	32.4	27.2	29.5	31.9	36.6	36.4			CH_3_CH_2_O		24-OCH_3_	169.9		
C17	50.1	50.1	50.2	50.0	44.3	45.4	52.4			14.4		51.8			

Notes: NO. **1** N-BU 2' 30.8, N-BU 3' 19.3, N-BU 4' 13.9; NO. **2** Bu 4' 13.9; NO. **3** Bu 4' 13.9; NO. **24** OCH_2_CH_3_ 14.1; NO. **29** OCOCH_3_ 20.9; NO. **41** 15-COCH_3_ 21.3; NO.**65** 15-OCOCH_3_ 21.1; NO. **73** 7-OCH_3_ 55.6; NO. **74** 7-OCH_3_ 54.5; NO.**77** AcCH_3_ 21.4, AcCH_3_ 21.0; NO. **78** 3-OCOCH_3_ 21.2, 22-OCOCH_3_ 170.6, 22-OCOCH_3_ 21.0; NO. **79** 22-OCOCH_3_ 21.0; NO. **80** 15-OCOCH_3_ 21.1, 22-OCOCH_3_, 170.6, 22-OCOCH_3_ 21.0; NO. **81** 22-OCOCH_3_ 21.1; NO. **82** 22-OCOCH_3_ 170.6, 22-OCOCH_3_ 21.0; NO. **83** AcCH_3_ 21.1, AcCH_3_ 21.3, AcCH_3_ 21.3, CO 170.3, CO 170.3, CO 170.5; NO. **84** AcCH_3_ 21.0, AcCH_3_ 21.6, AcCH_3_ 21.7, CO 170.6, CO 170.1, CO 170.2, OCH_3_ 55.2; NO. **99** COOCH_3 _51.9; NO. **100** OCH_3_ 51.9; NO. **101** OCH_3_ 51.9, COCH_3_ 20.9, COCH_3_ 170.2; NO. **102** OCH_3_ 51.8, COCH_3_ 20.8, COCH_3_ 170.1; NO. **103** OCH_3_ 51.9; NO. **104** OCH_3_ 51.9; NO. **105** OCH_3_ 51.9; NO. **118** C3' 73.7, C4' 69.7, C5' 63.7, COCH_3_ 170.9, COCH_3_ 21.4; NO. **120** C2' 74.0, C3' 79.2, C4' 71.4, C5' 79.0, C6' 62.5; NO. **127** OCH_3_ 52.0; NO. **138 **COO-CH_2_CH_3_ 60.9, COOCH_2_CH_3_ 14.4, CO of AcO-C(3) 171.1, CH_3_ of AcO-C(3) 21.5; NO. **139** COOCH_2_CH_3_ 60.8, COOCH_2_CH_3_ 14.4; NO. **145** C24' 6.8; NO.** 149** CH_3_CO 170.4; NO. **150** CH_3_CO 21.4, CH_3_CO 21.3; NO. **151** C23 31.8(24S), C24 75.6(24S), C25 149.8(24S), C26 111.5(24S), C27 18.1(24S); NO. **153** C24' 21.5; NO. **167** AcCO 170.8, AcCO 171.0.; NO. **172** AcCH_3_ 21.2, AcCH_3_ 21.4, AcCH_3_ 21.5, CO 170.7, CO 170.9, CO 170.2; NO. **173** AcCH_3_ 21.0, CO 170.6; NO. **174** AcCH_3 _21.0, AcCH_3_ 21.3, CO 170.6, CO 170.6; NO. **176** AcCO 170.0, AcCH_3_ 21.4, AcCH_3_ 21.3, AcCH_3_ 21.0; NO. **178** AcCH_3_ 21.2, AcCH_3_ 21.2; NO. **184** 15-OCOCH_3_ 21.4, 22-OCOCH_3_ 170.6, 22-OCOCH_3_ 21.0; NO. **185** AcCH_3_ 21.5, CO 170.6, CO 171.2; NO. **186** AcCH_3_ 21.7, CO 170.7, CO 170.9; NO. **193** C4' 20.9; NO. **222** Bu3' 19.3, Bu4' 13.9; NO. **223** Bu3' 19.3, Bu4' 13.9; NO. **226** COOCH_3_ 20.8, OCH_3_ 51.4, COCH_3_ 170.0; NO. **230** COCH_3_ 20.8, COCH_3_ 170.1; NO. **231** OCH_3_ 51.6, COCH_3_ 20.9, COCH_3_ 170.1; NO. **232** OCH_3_ 51.7; NO. **233** C33 60.5, C34 14.2; NO. **243** OCH_3_ 51.6, COCH_3 _20.8, COCH_3_ 170.0; NO. **244** OCH_3_ 51.6, COCH_3_ 20.9, COCH_3_ 170.1; NO. **249** C3' 19.3, C4' 13.9; NO. **275** C3' 145.2, C4' 34.5 C5' 25.6, C6' 122.1, C7' 136.9, C8' 39.0, C9' 25.6, C10' 125.5, C11' 134.5, C12' 38.7, C13' 13.8, C14' 16.2, C15', 169.0, C1'' 157.1, C2'' 112.7, C3'' 117.3, C4'' 148.2, C5'' 126.0, C6'' 119.8; NO. **276** C3' 133.3, C4' 35.9 C5' 28.5, C6' 124.6, C7' 136.7, C8' 38.9, C9' 27.3, C10' 126.8, C11' 135.8, C12' 69.0, C13' 13.7, C14' 16.2, C15' 172.3, C1'' 128.0, C2'' 149.3, C3'' 114.8, C4'' 151.2, C5'' 116.9, C6'' 117.8; NO. **277** C3' 27.0; NO. **278** CH_3_ 26.4, CH_3_ 23.5; NO. **288 **C3' 146.3, C4' 34.6C5' 25.5, C6' 122.1, C7' 136.6, C8' 39.1, C9' 25.5, C10' 125.3, C11' 134.4, C12' 68.6, C13' 13.9, C14' 16.3, C15' 168.3, C1'' 157.0, C2'' 114.9, C3'' 117.2, C4'' 148.1, C5'' 126.1, C6'' 119.8; NO. **291** CH_3_ 21.4; NO. **295 **C2' 21.1; NO. **296** C2' 21.1; NO. **297** C2' 46.4, C3' 69.9 C4' 46.1, C5' 171. 9,3'-CH_3_ 28.4, OCH_3_ 51.2, C31 107.1; NO. **298** C2' 21.1; NO. **302** C2' 72.3, C3' 75.9, C4' 69.5, C5' 65.8, COCH_3_ 170.9, COCH_3_ 21.4, C241 25.7; NO. **303** 22-OCOCH_3_ 171.3, 22-OCOCH_3_, 21.2; NO. **304** C2' 20.7, C1'' 170.9, C2'' 21.1, OCH_3_ 51.8; NO. **305** C2' 21.1, C1'' 170.8, C2'' 21.2, OCH_3_ 51.8; NO. **306 **C2' 20.7, OCH_3_ 51.4. (a) Measured in C_5_D_5_N; (b) Measured in CDCl_3_; (c) Measured in CD_3_OD(50%) and CDCl_3_(50%); (d) Measured in DMSO-d_6_; (e) Measured in pyridine-d_5_; (f) Measured in CD_3_OD; (g) Measured in (CD_3_)_2_CO; (h) Measured in C_6_D_6_.

## 4. The Bioactivities of *Ganoderma* Triterpenes

### 4.1. Anti-Tumor Activity

Cancer has been acknowledged as a huge threat to human health and most governments are committed to diminish this threat. The urgent task of finding anti-tumor drugs with high efficiency and low toxicity have drawn countless researchers’ efforts directed to the discovery of lead compounds or bioactive ingredients from nature resources such as *Ganoderma*. The GTs were extensively evaluated for cytotoxic activities against a series of tumor cell lines. Compounds **45**, **46**, **164** and **204** showed cytotoxic effects against the tested tumor cell lines. Compound **46** exhibited the most potent cytotoxicity against LLC, T-47D, Sarcoma 180 and Meth-A tumor cells [25]. Compounds **62**, **190** and **212** showed strong cytotoxic activities against human Hela cervical cancer cells [26]. According to Cheng’s report, the ganoderic alcohols showed stronger activities than ganoderic acids which implies that a hydroxy group substituted at 26 may be a very important structural feature for cytotoxic activity, however, the more hydroxyl groups there are, the lower the inhibitory activity will be [26]. Compounds **42** and **85** showed cytotoxicity against p388, Hela, BEL-7402, and SGC-7901 human cancer cell lines, with IC_50_ values in the 8–25 μM range [32]. Compounds **47**–**52** were studied *in vitro* against Meth-A and LLC tumor cell lines [37]. Compound **187** displayed selective inhibitory activity against HL-60 cells, and compound **131** exhibited selective cytotoxic activity against MCF-7 cells. Compounds **7**, **67** and **188** showed the ability to induce hPXR-mediated CYP3A4 expression [47]. Compounds **9**, **23**, **57** and **68** showed significant cytotoxic activity, with IC_50_ values of 18.7, 21.4, 16.2 and 20.1 μg/mL, respectively [48]. Compounds **77**, **163**, **170** and **173** were tested *in vitro* for their cytotoxic activities against 95D and Hela tumor cell lines with IC_50_ values ranging from 14.7 to 38.5 μM [49]. Compound **121** showed significant activity against T-24 cells, while compounds **119**, **123**, showed significant activity against T-24, HT-3, and CaSKi cells, respectively [60]. Compound **297** showed significant cytotoxic activity with an IC_50_ value of 2.5 μg/mL in the Hep-2 cell line [62]. Treatment of human hepatoma HuH-7 cells with compound **205** caused immediate inhibition of DNA synthesis as well as activation of ERK and JNK mitogen-activated protein kinases, and cell apoptosis. Molecular events of apoptosis including degradation of chromosomal DNA, decrease in the level of Bcl-xL, the disruption of mitochondrial membrane, cytosolic release of cytocherome c and activation of caspase-3 were elucidated. The ability of compound **205** to inhibit topoisomerases and to sensitize cancer cells towards apoptosis meets the criteria of a potential anticancer drug [88]. Compounds **30**, **229** and **235** showed significant cytotoxic activities against Hep G2, Hep G2,2,15, and P-388 cell lines [91]. Compound **233** showed cytotoxicity against HL-60 and CA46 cancer cell lines [93]. Biological activity as an anti-tumor promoter was observed for compounds **279**–**282** [101]. Compound **285** showed moderate cytotoxicity against liver cancer and lung cancer cell lines [27]. Compounds **140**, **279**, **281**, **287**, **292** and **312** inhibited the viability and growth of the HL-60 cell lines [103].

### 4.2. Anti-HIV and Anti-HIV-1 Protease Activity

It was reported that compounds **270**, **272**, **291** and **304**–**306** were inhibitory against HIV-1 protease, with IC_50_ values for the most potent compounds ranging from 5 μg∙mL^−^^1^ to 13 μg∙mL^−^^1^ [102]. Moreover, compounds **190** and **210** were found to be active as anti-HIV-1 agents with an inhibitory concentration of 7.8 μg∙mL^−^^1^ for both, and compounds **4**, **11**, **23**, **28**, **171** and **203** were moderately active inhibitors against HIV-1 protease with a 50% inhibitory concentration of 0.17–0.23 mM [18]. While compounds **5**, **53**, **201** and **204** showed significant anti-HIV-1 protease activity with IC_50_ values of 20–90 μM [38]. In addition, compounds **39**, **224** and **255** inhibited human immunodeficiency virus-1 protease with IC_50_ values of 20–24 μM.

### 4.3. Neurotrophic Activity

A series of reports has shown that *Ganoderma* triterpenes exhibit neurotrophic activity. Bioassay results revealed that compounds **12** and **261** have nerve growth factor-like neuronal survival-promoting effects, whereas the two compounds mentioned above and compounds **10**, **159** and **183** showed brain-derived neurotrophic factor-like neuronal survival-promoting activities [73]. Compounds **1** and **278**, exhibiting specific anti-acetylc-holinesterase activity, are being examined as possible drug candidates for the treatment of Alzheimer’s and related neurodegenerative diseases. Compounds **62**, **204**, **210** and some other *Ganoderma* triterpenes exhibited moderate acetylcholinesterase-inhibitory activity, with IC_50_ values ranging from 9.40 to 31.03 μM. These results indicated that these lanostane triterpenes are preferential inhibitors of acetylcholinesterase and may be suitable as drug candidates [16].

### 4.4. Hepatoprotection

It is also reported that compound **11** showed significant hepatoprotective activity. However, increased doses of compound **11** (up to 10 times) did not further reduce GOT/GPT levels in the serum of the mice [107]. Compound **144** has an activity of lowering the levels GPT in mice with liver injury by CCl_4_ and GaNI and exhibits hepatoprotective effects [67].

### 4.5. Antiobesity Activity

In 2010, the inhibitory effect of triterpenes isolated from *G. lucidum* on adipocyte differentiation in 3T3-L1 cells was reported for the first time [17]. According to a report on the subsequent research, compound **249** reduced the triglyceride accumulation significantly by 72% at 80 μM and it effectively suppressed the glycerol-3-phosphate dehydrogenase activity in the cells. It suppressed the gene expressed of PPARγ, C/EBPα, and SREBP-1c in a dose-dependent manner during differentiation. These findings demonstrate that compound **249** contributes to the inhibitory effect on adipocyte differentiation in 3T3-L1 cells [96].

### 4.6. Hypoglycemic Activity

The inhibitory effect on aldose reductase was examined for compound **27** and its methyl ester. The results indicated that the IC_50_ of **27** is 22.8 μM, whereas that of its methyl ester is more than 200 μM, which suggested that a carboxyl group of side chain of compound **27** is essential for potent inhibitory activity because of much lower level of inhibitory activity of its methyl ester. However, the exact reason for the difference in inhibition between compound **27** and its methyl ester remains unclear [29]. Compound **169** was also found to have high α-glucosidase inhibition, with IC_50_ of 119.8 μM [108].

### 4.7. Other Bioactivities

*Ganoderma* has been investigated for other bioactivities. Compounds **45** and **58** were found to exhibit potent inhibitory activity against herpes simplex virus [42]. Compounds **13** and **15** were shown to inhibit histamine release from rat mast cells [21]. In the study on compounds **3** and **156**, it was found they both exhibited inhibitory activities against the HMG-CoA reductase and acyl CoA acyltransferase [35]. Another study demonstrated that compounds **44** and **49** exhibited potent enhancement of ConA-induced mice splenocytes proliferation *in* *vitro* [36]. It was found that compounds **161**, **189** and **316** possess the bioactivity to induce apoptosis in human promyelocytic leukemia HL-60 cells [75]. An investigation on the ability of some* Ganoderma* triterpenes to inhibit 5α-reductase in rat liver microsomes revealed that compounds **64**, **161** and **206** showed the inhibitory activity. Further study suggested that a carboxyl group of the 17β-side chain of compound **206** was essential to elicit the inhibitory activity [89]. The *in vitro* tests showed that compounds **308** and **310** exhibited modest inhibitory activity against rabbit platelet aggregation induced by platelet activating factor (PAF), and compound **310** also displayed weak inhibition against platelet aggregation induced by adenosine diphosphate (ADP) [105]. The C-3 epimer of compound **172** also exhibited significant antimycobacterial activity against mycobacterium tuberculosis H37Ra [46].

## 5. Conclusions

*Ganoderma* triterpenes (GTs) are a class of compounds with various chemical structures and a diverse range of biological activities. Biomedical analysis has shown that triterpenes possess important pharmacological activities and are thought to be potential candidates for drug discovery, but their low abundance, complex procedures of extraction and purification, the difficult preparation of high purity triterpenoids from *G .lucidum* is currently limited at the laboratory scale. Thus, how to enhance the content of triterpenoids and improve the technology of the extraction and purification of triterpenoids from *Ganoderma lucidum* is a problem that needs to be solved. We can expect to enhance GT production through the regulation of GA biosynthesis, thus promoting the industrial development of *G*. *lucidum* and provide an important resource for the development and application of new antineoplastic, anti-HIV, and other drugs.

Based on the above analysis of structural complexity and functional group variety, it is especially important to prove the structure-function relationships to make up for the inadequacy of this aspect. Although extensive research has been done on this herb, there is still a lot of scope for further research, especially on the mechanisms of biological activity of GTs with emphasis on agents with anti-tumor, anti-HIV, neurotrophic properties. *G. lucidum* and *G. sinense* that are recorded in the pharmacopoeia of China in 2010 have been widely applied in China [8]. Their long-standing medicinal history indicates their irreplaceable functions. In further study, researchers may need to pay more attention to the two species, and focus on the active substances such as the triterpenes summarized above. To achieve better quality control, the studies on other species are also important, so that the differences between species can be illustrated clearly. Additionally, more important bioactive constituents should be integrated into the quality control system of *Ganoderma*. Further experiments including *in vitro*, *in vivo* and clinical studies should be encouraged to identify any potential side effects.
